# Synthesis and Properties of Cyclic Imide Extended Diazocines: Tweezer‐Like, Rigid Photoswitches with Large Switching Amplitudes

**DOI:** 10.1002/chem.202500435

**Published:** 2025-04-03

**Authors:** Artjom Businski, Thuy C. Ta, Lara Unterriker, Niklas Gindullis, Jan‐Simon von Glasenapp, Christian Näther, Rainer Herges

**Affiliations:** ^1^ Otto Diels Institute of Organic Chemistry Kiel University Kiel Germany; ^2^ Institute of Organic Chemistry Hannover University Hannover Germany; ^3^ Institute of Inorganic Chemistry Kiel University Kiel Germany

**Keywords:** azo compound, diazocine, imide, late‐stage‐derivatization, photochemistry, photochromism, photoswitch

## Abstract

Diazocines are bridged azobenzenes with improved photophysical properties. One of the main features is the fact that, unlike the azobenzenes, the *Z* form is more stable than the *E* isomer. Another important property is the more rigid structure (tricyclic), which precisely defines the molecular movement. The disadvantage of diazocines compared to azobenzenes is the less well‐developed chemistry, particularly regarding the synthesis of derivatives. In this work, we present an approach to preparing diazocine derivatives via a late‐stage functionalization strategy. Two key compounds are used for this purpose: a bis‐anhydride and a bis‐imide of diazocine. They can be further functionalized with both nucleophiles and electrophiles leading to numerous derivatives of diazocine with a large range of functional groups. Due to the extended *π* systems, the associated larger molecular switching lever, and the defined direction of molecular movement, numerous potential applications in the field of photoswitchable biological systems and photoresponsive materials are conceivable, e.g. photopharmacology, polymer‐based actuators, mechanophores, photoresponsive cages, helicates, MOFs, and COFs.

## Graphical abstract

Photoswitches with a perfect tweezer‐like bending motion and a large amplitude of movement are ideally suited as molecular actuators for applications in photoresponsive materials, biomolecules and photopharmacology. For this purpose, the core of the diazocine photoswitch was extended via cyclic imides. The compounds can be easily synthesized and late‐stage functionalized.

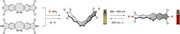

## 1 Introduction

**Figure 1:**
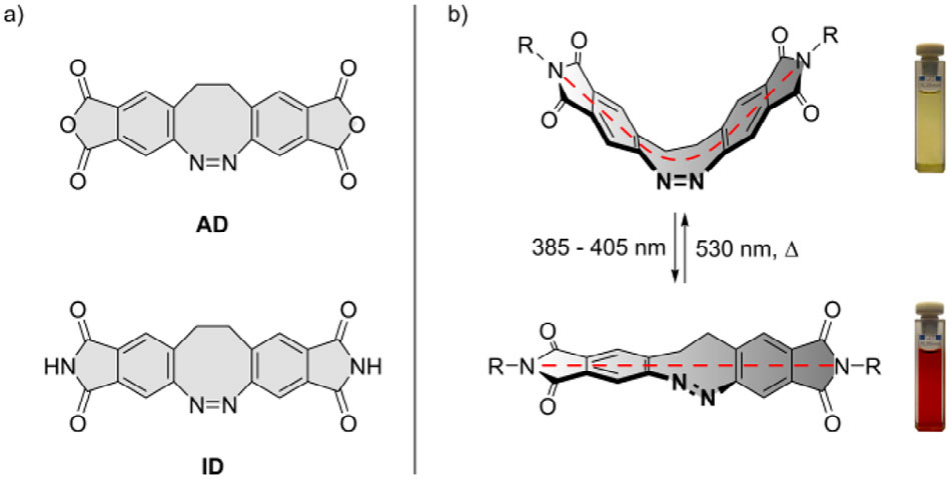
(a) Structures of anhydride diazocine (AD, top) and imide diazocine (ID, bottom). (b) Photoisomerization of substituted imide diazocines (IDs), which is accompanied by a color change from yellow to red. The longitudinal axis (dashed red line) coincides with the axis of the bending motion.

**Figure 2:**
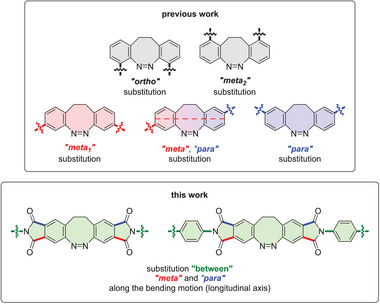
Disubstituted diazocines of previous works^[^ ^21^, ^28^, ^30^, ^39^, ^40^, ^41^, ^42^, ^43^, ^44^, ^45^, ^46^, ^47^, ^48^, ^49^, ^50^ ^]^ (top) as well as the novel substitution pattern of imide diazocines (IDs, bottom) presented in this work.

Azo compounds are arguably the most commonly used photoswitches in chemistry. They have a wide range of applications due to their unique photochemical and photophysical properties that are based on reversible *Z* ⇄ *E* isomerization when exposed to specific wavelengths of light.^[^ ^1^, ^2^ ^]^ Key applications are photonics,^[^ ^3^ ^]^ optoelectronics,^[^ ^4^ ^]^ molecular machines,^[^ ^5^ ^]^ out‐of‐equilibrium systems,^[^ ^6^ ^]^ smart polymers,^[^ ^7^, ^8^, ^9^, ^10^ ^]^ photoresponsive thin films,^[^ ^11^, ^12^ ^]^ energy conversion systems,^[^ ^13^ ^]^ drug delivery,^[^ ^14^, ^15^ ^]^ photoswitchable biomolecules,^[^ ^16^, ^17^ ^]^ photopharmacology,^[^ ^18^ ^]^ and photoresponsive coordination compounds.^[^ ^19^ ^–‐^ ^22^ ^]^ Diazocines are azo compounds that contain an eight‐membered heterocyclic ring including the azo (N═N) group.^[^ ^23^, ^24^, ^25^ ^]^ Due to the ring strain in the central eight‐membered ring, diazocines, unlike azobenzenes, are thermodynamically more stable in their *Z* configuration (angulated boat conformation) than in their *E* form (stretched twist conformation).^[^ ^26^ ^]^ This “inverted stability” has a number of advantages, especially in photopharmacology^[^ ^27^, ^28^, ^29^, ^30^ ^]^ and in stimuli‐responsive materials.^[^ ^31^, ^32^, ^33^, ^34^ ^]^ Further advantages over the simple azobenzenes are higher switching efficiencies, higher quantum yields in the conversion of light into molecular motion (*Z* ⇄ *E* isomerization), and switching wavelengths shifted into the visible range.^[^ ^35^, ^36^ ^]^ The number of applications for diazocines is nevertheless limited, as derivatives are more difficult to access synthetically. Compared to azobenzenes, the chemistry of diazocines is less well‐developed. At first glance, one would assume, that the two benzene units of diazocine can be derivatized via classical, electrophilic aromatic substitutions, as it is in some cases possible in azobenzene.^[^ ^37^, ^38^ ^]^ All attempts in this direction in our and other research groups have so far failed. Substituted diazocines are only accessible if they are built up from the corresponding substituted precursors. This means that the multi‐step synthesis procedure must be optimized independently for each diazocine derivative. To solve this problem, we have developed a modular strategy to functionalize diazocines in a late stage. We prepared two key compounds, the bis‐anhydride (anhydride diazocine, AD) and the bis‐imide (imide diazocine, ID) of diazocine (Figure 1). By reacting AD with amines (nucleophiles) and ID with electrophiles a large number of diazocine derivatives are accessible within one step. Our new synthesis strategy also allows the realization of a new substitution pattern on diazocine and thus a molecular movement not previously realized in other photochromic systems, which enables better force exertion. The extension of the diazocine framework with two cyclic imides has another very important advantage over previous designs. It improves the interaction of the system with the environment. In most applications (actuators, mechanophores, photoswitchable biomolecules, photopharmacology) the small geometry change of the azo group must be transferred to a larger displacement of the substituents of the azobenzene or diazocine. The larger the displacement of the substituents, the more effectively the change in geometry caused by the azo group is transferred to the environment. How large the movement is, depends on where the substituents are located. With the almost perfect bending movement along the longitudinal axis (red dashed line in Figure 1) of the diazocine molecule, it is advantageous if the bonds of the substituents to the switch coincide with the vectors that describe the bend. It is also advantageous if the lever is as long as possible. There are a number of conceivable substitution patterns in the basic diazocine body (Figure 2). None of them fulfill the above condition. The red dashed line intersects the benzene rings between the meta and para positions. By anellation of the benzene rings with a five‐membered ring (cyclic imide) and substituting the N atom, the movement of the substituents is almost exactly in line with the vectors of the bending motion (red dashed line in Figure 1). The effectiveness of the transfer of the bending motion from the azo group to a pair of substituents can be estimated as the change in their distance (Δ) upon isomerization (Figure S95). In the parent diazocine, the force transfer is most effective when the substituents are in meta and para position to the azo group (Δ = 3.0–3.5 Å). In ID **15**, the change in distance Δ is 5.3 Å and in the phenyl substituted ID **9** the distance of the terminal H atoms changes from 14.3 to 23.3 Å (Δ = 9.0 Å) (Figure S95).^[^ ^51^, ^52^, ^53^, ^54^, ^55^, ^56^ ^]^

## 2 Results and Discussion

### 2.1 Synthesis

**Scheme 1:**
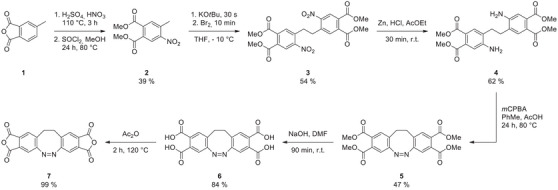
Synthesis of AD **7** over six reaction steps starting from commercially available 4‐methylphthalic anhydride **1**.

**Scheme 2:**
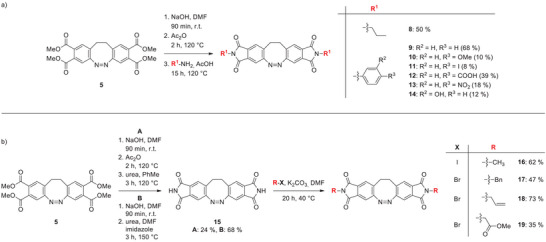
(a) Synthesis of IDs **8–14** over three steps using reaction intermediates. (b) Synthesis of ID **15** via procedure **A** and **B** as well as IDs **16–19**.

Since anhydride groups are versatile and reactive electrophiles and not compatible with the synthesis of the diazocine framework, we decided to perform the anhydride formation in the last step. Towards this end, we used the corresponding dicarboxylic methyl esters as “masked anhydrides” to prepare the diazocine core. Starting from commercially available 4‐methylphthalic anhydride **1**, first a two‐step reaction including nitration and esterification using thionyl chloride in methanol was carried out successfully at a 250‐g scale (Scheme 1).^[^ ^57^ ^]^ Two regioisomers are formed, which are easy to separate by recrystallization. The nitrated dimethyl phthalate **2** underwent oxidative dimerization by successive addition of KO*t*Bu and bromine in large scales of up to 10 g, forming the corresponding dimer **3** in 54% yield.^[^ ^58^, ^59^ ^]^ Subsequently, a reduction of the nitro groups was performed using zinc in concentrated hydrochloric acid and ethyl acetate yielding 62% of the corresponding amine substituted dimer **4**.^[^ ^57^ ^]^ For the formation of the ester functionalized diazocine **5**, an oxidative azo cyclization with the amine **4** and *m*CPBA as the oxidant in toluene and acetic acid was carried out in 47% yield.^[^ ^50^ ^]^ The tetraester substituted diazocine **5** was then hydrolyzed using 30 wt% NaOH solution in DMF, which gave the corresponding tetracarboxylic acid substituted diazocine **6** in 84 % yield.^[^ ^60^ ^]^ Finally, refluxing of the diazocine **6** in pure acetic anhydride provided the desired AD **7** in almost quantitative yield.^[^ ^61^ ^]^ According to NMR, the reaction was complete, and only less than 1% of a by‐product was formed, which could not be identified. However, it is most probably not a diazocine derivative since it is not photoswitchable. Purification of AD **7** by column chromatography using silica gel or Florisil®, recrystallization, or precipitation was unsuccessful, leading to the decomposition of AD **7**. Also, storage of the crude product under ambient conditions for a few days in solution or as a solid leads to decomposition. Due to the instability of AD **7**, the anhydride was not isolated and characterized in pure form. The stable tetraester **5**, therefore, is a more suitable stock for further syntheses. AD **7** was freshly prepared each time and used directly without any further purification. Due to its high reactivity, AD **7** reacts readily with primary amines to form five‐membered cyclic imides (Scheme 2). For practical reasons (see above), however, we started from the tetraester diazocine **5**, in three steps without isolation of intermediates. Tetraester **5** was first hydrolyzed to the tetracarboxylic acid **6**, condensated with acetic anhydride to the anhydride **7**, and then converted with primary aliphatic and aromatic amines in acetic acid to the cyclic IDs **8–14** in yields of 8%–68 %.^[^ ^62^ ^]^ In contrast to the cyclic anhydride **7**, the cyclic imides **8**–**14** could be characterized and isolated as stable compounds. An alternative access to substituted IDs is provided by the reaction of the parent unsubstituted imide **15** with electrophiles (Scheme 2). This inversion of reactivity makes aliphatic derivatives in particular easily accessible. The parent ID **15** was prepared from the ester diazocine **5** by hydrolysis, condensation, and imidization with urea in xylene in 24% yield.^[^ ^63^ ^]^ Alternatively, ID **15** can also be obtained directly from the tetracarboxylic acid **6** by reaction with urea using imidazole as an activator.^[^ ^64^ ^]^ This improved the yield of ID **15** to 68%. The reaction of ID **15** with several halide electrophiles and potassium carbonate as a base in DMF yielded the IDs **16–19** in 35%–73% (Scheme 2).^[^ ^62^ ^]^

### 2.2 Photophysical Properties

**Figure 3:**
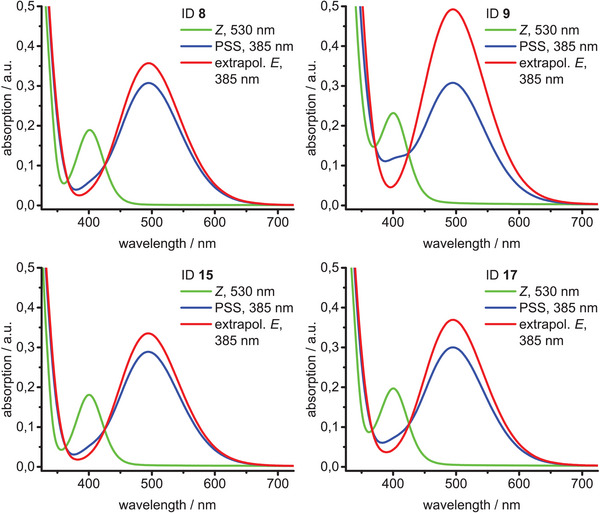
UV/vis spectra (THF, 300 µM, 25°C) of IDs **8** and **9** (top) as well as **15** and **17** (bottom).

Compared with parent diazocine, UV/vis photo characterization of the novel IDs **8–19** revealed a very small hypsochromic shift in absorption maxima between 396–401 nm of the respective *Z* isomers, whereas a small bathochromic shift of absorption maxima between 494–495 nm of the corresponding *E* isomers was observed (Table 1).^[^ ^35^, ^36^ ^]^ The thermal half‐lives of the metastable *E* isomers of the diazocines **5–19**, which are in the range of 13.4–52.0 min are on average about ten times shorter than the half‐life of the parent diazocine.^[^ ^35^, ^36^ ^]^ The conversion rates of the *Z* → *E* isomerizations are dependent on the substituent on the imide N atom. In general, derivatives with aliphatic substituents (**8** and **16–19**) upon irradiation with 385–405 nm (blue light) exhibit high photostationary equilibria in the range of 81%–86%. Lower conversion rates of 36%–62% were observed for diazocines **9–14** bearing substituted phenyl groups. Photoconversion rates for the *E* → *Z* isomerization (back‐isomerization) of compounds **5–19** with 530 nm (green light) are quantitative within the detection limits of our analytical methods. Although the substituents on the imide N atom are relatively distant from the azo group in diazocine (6 bonds), there is a conjugative interaction, that affects the conversion rate and the UV/vis spectra. While the UV absorption bands of the nπ* transitions at about 400 nm (*Z* isomer) and 495 nm (*E* isomer) hardly change, the substitution on the imide nitrogen atom has a noticeable influence on the ππ* band. Obviously, the extension of the *π* system causes a bathochromic shift of the ππ* band and thus a stronger overlap with the nπ* band of the *Z* isomer (compare UV/vis spectra of ID **8** or ID **15** with ID **9** in Figure 3). This decreases the conversion rate of the *Z* → *E* isomerization. The conjugative effect becomes particularly obvious when comparing the phenyl (ID **9**) and benzyl‐substituted (ID **17**) systems (Figure 3). The methylene group in ID **17** obviously interrupts the conjugation, which leads to a higher conversion rate. During the photoisomerization of phenyl‐substituted IDs **9**–**14** in the mM concentration range, we made a remarkable observation. When irradiating the thermodynamically stable *Z* form in THF and many other organic solvents with 385–405 nm a red solid precipitates, which does not change even after prolonged storage at room temperature (Figure S110). The precipitate dissolves in DMSO and could be identified as the *E* isomer. We explain this phenomenon by strong *ππ* interactions of the extended *π* systems of the stretched *E* isomers, which are stabilized by the formation of stacks in the solid state. Such an interaction is not possible in the bent *Z* form, which leads to a better solubility of the *Z* isomer. This effect can be utilized to isolate the phenyl‐substituted IDs **9**–**14**. Precipitation of the *E* isomer after irradiation was not observed with the non‐phenyl substituted IDs **8** and **16–19**.

**Table 1:** Absorption maxima *λ*_max_ of *Z* and *E* isomers, reaction rate constants *k* of thermal relaxation, half‐lives *t*_1/2_ and photoconversion rates Γ of diazocines **5–19**.

compound	5^[^ ^[a]^, ^[b]^ ^]^	6^[^ ^[a]^, ^[b]^ ^]^	7^[^ ^c^ ^]^	8^[^ ^[a]^, ^[b]^ ^]^	9^[^ ^[a]^, ^[d]^ ^]^	10^[^ ^[a]^, ^[b]^ ^]^	11^[^ ^[a]^, ^[b]^ ^]^	12^[^ ^[d]^, ^[e]^ ^]^	13^[^ ^[d]^, ^[e]^ ^]^	14^[^ ^[a]^, ^[b]^ ^]^	15^[^ ^[a]^, ^[b]^ ^]^	16^[^ ^[a]^, ^[b]^ ^]^	17^[^ ^[a]^, ^[b]^ ^]^	18^[^ ^[a]^, ^[b]^ ^]^	19^[^ ^[a]^, ^[b]^ ^]^
*λ*_max_ (*Z*) / nm	405	405	‐	401	400	396	400	397	401	400	400	400	400	400	401
*λ*_max_ (*E*) / nm	493	493	‐	495	495	494	494	495	494	495	494	495	495	495	495
*k* ∙ 10^−4^/ s^−1^	3.95	3.03	‐	4.38	5.38	2.22	6.26	6.71	8.63	5.50	3.69	4.70	4.92	4.59	5.44
*t*_1/2_ / min	29.3	38.1	‐	26.4	21.5	52.0	18.5	17.2	13.4	21.0	31.3	24.6	23.5	25.2	21.3
Γ*_Z_*_→_ *_E_* / %	88^[^ ^f^ ^]^	89^[^ ^f^ ^]^	‐	86^[^ ^f^ ^]^	62^[^ ^f^ ^]^	41^[^ ^g^ ^]^	36^[^ ^g^ ^]^	58^[^ ^f^ ^]^	53^[^ ^f^ ^]^	59^[^ ^g^ ^]^	88^[^ ^f^ ^]^	83^[^ ^f^ ^]^	81^[^ ^f^ ^]^	84^[^ ^f^ ^]^	85^[^ ^f^ ^]^
Γ*_E_*_→_ *_Z_* / %^[^ ^h^ ^]^	>99	>99	‐	>99	>99	>99	>99	>99	>99	>99	>99	>99	>99	>99	>99

^[a]^ UV/vis measurements in THF at 25°C.

^[b]^ NMR measurements in THF‐*d_8_* at 0°C.

^[c]^ Determination of photophysical data could not be carried out due to decomposition of AD **7**.

^[d]^ NMR measurements in DMSO‐*d_6_* at 25°C.

^[e]^ UV/vis measurements in DMSO at 25°C.

^[f]^ Wavelength used for *Z* → *E* conversion (Γ*_Z_*_ →_ *_E_*): 385 nm.

^[g]^ Wavelength used for *Z* → *E* conversion (Γ*_Z_*_→_ *_E_*): 405 nm.

^[h]^ Wavelength used for *E* → *Z* conversion (Γ*_E_*_→_ *_Z_*): 530 nm.

### 2.3 Crystal Structures

**Figure 4:**
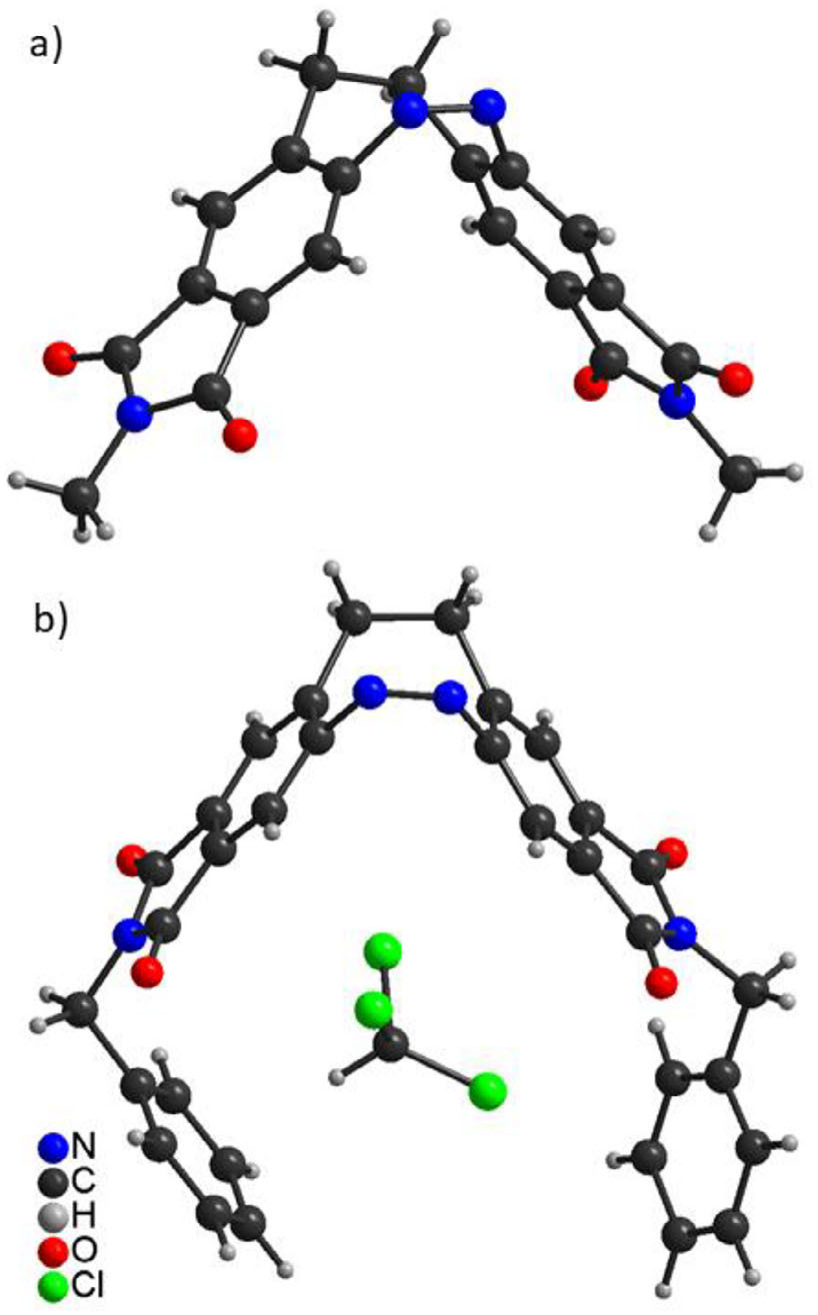
(a) Crystal structure of ID **16** in the *Z* form predicting further possibilities of functionalization along the central bending axis. (b) Crystal structure of ID **17** in the *Z* form showing a tweezer‐like arrangement with encapsulation of chloroform in the semi‐closed cavity.

Generally, most of the diazocines, synthesized in this work crystallize particularly well in their thermodynamically stable *Z* form, which can be attributed to their high symmetry and the efficient packing of the phthalimide subunits. A total of seven crystal structures of the diazocines **5**, **8**, **9**, **10**, **16**, **17**, and **18** were obtained, which provide further information about structural properties and hints for possible applications. The structures of IDs **16** and **17** impressively illustrate the tweezer‐type function (Figure 4). The extension of the benzene ring in parent diazocine by a five‐membered imide unit in **16** extends the “lever” of the bending motion and allows further structural extension along the bending axis. In ID **17** the tweezer arms are additionally extended by benzyl groups on each side. The crystal structure of the *Z* isomer of ID **17** includes a chloroform molecule in a semi‐closed cavity. Crystal structures of *n*‐propyl‐substituted diazocine **8** (Figure S97), phenyl‐substituted diazocine **9** (Figure S98), and *p*‐methoxyphenyl‐substitituted diazocine **10** (Figure S99) also include solvent molecules in their cavity. Unfortunately, no crystal structures from the metastable stretched *E* isomers of aryl substituted IDs **9**–**14** could be obtained because of rapid precipitation as amorphous powders. Theoretical calculations, however, predict an almost perfectly linear stretched geometry of the *E* isomers (Figure S92,94).^[^ ^51^, ^52^, ^53^, ^54^, ^55^, ^56^ ^]^ Considering the geometry change upon *Z* ⇆ *E* isomerization, it is quite self‐evident and intuitive that the imide diazocines should be ideally suited as light‐driven molecular tweezers.

## 3 Conclusion

Photoswitches convert light energy into molecular movement. A useful function arises when the molecular movement is coupled efficiently and in a defined manner with the environment. Due to their rigid, tricyclic structure, diazocines have fewer conformational degrees of freedom than most conventional photoswitches (azobenzenes, diarylethenes, spiropyrans) and therefore perform a defined bending motion during photoisomerization. By extending the diazocine in the direction of the bending axis with two additional cyclic five‐membered imides, a tweezer‐like movement is achieved. By substitution at the imide nitrogen atom, the lever can be further extended and thus tailor‐made systems can be synthesized. The key compound in the preparation is the tetraester substituted diazocine **5**, which is accessible in a four‐step synthesis on a gram scale from commercially available starting materials. From the tetraester **5**, the corresponding AD **7** or ID **15** is prepared, which in turn are easily substituted by reaction with primary amines (with anhydride **7**) or electrophiles (with imide **15**). A total of twelve new imide diazocines **8–19** were synthesized using this late‐stage strategy, including three diazocine precursors **5–7** of the imides. Of these, seven compounds **5**, **8**, **9**, **10**, **16**, **17**, and **18** were characterized by X‐ray structure analysis. The benzyl‐substituted system **17** shows impressively the pincer‐like effect by the inclusion of chloroform (Figure 3). By exposing the thermodynamically stable *Z* configuration to light with a wavelength of 385–405 nm, a conversion to the *E* form of 36%–89% is achieved, whereby the imide diazocines **8** and **16–19** (carbon sp^3^ substituted at the imide N) show a higher switching efficiency. The half‐lives of the metastable *E* isomers are 13.4–52.0 min. The *E* forms of the phenyl‐substituted systems **9**–**14** precipitate as a stable red solid when exposed to blue light (385–405 nm) in most organic solvents. The modular synthesis and favorable photophysical properties make these diazocines promising candidates for a range of applications.

## 4 Experimental Section

### 4.1 General procedure for the synthesis of substituted IDs **8–14** starting from tetraester diazocine **5**

The tetraester diazocine (**5**, 200 mg, 454 µmol) and an aqueous solution of NaOH (30 wt%, 2.00 mL) were placed in DMF (12.0 mL) and stirred at room temperature for 90 min. The reaction mixture was quenched with ice and acidified with HCl (2 M) until pH = 1 was reached. The aqueous phase was extracted twice with DCM (50.0 mL each), whereupon the organic phase was extracted twice with water (100 mL) again. The organic phase consisting of DCM was disposed of. A saturated aqueous solution of NaCl (100 mL) was added to the combined aqueous phase, which was extracted five times with ethyl acetate (100 mL each). The combined organic phase was dried over MgSO_4_, filtered off, and the solvent was removed in vacuo. The resulting residue (a crude product that mainly contains tetracarboxylic acid diazocine **6**) was dissolved in Ac_2_O (50.0 mL) and stirred at 120°C for 2 h. The solvent was removed in vacuo. The resulting residue (a crude product that mainly contains anhydride diazocine **7**) and the respective amine (908 µmol) were dissolved in concentrated AcOH (45.0 mL) and stirred at 120°C for 15 h. The reaction mixture was quenched with ice. Different purification methods depending on the amine used were carried out (details see SI). The product was obtained as a yellow solid.

### 4.2 General procedure for the synthesis of substituted IDs **16–19** starting from parent ID **15**

The parent imide diazocine (**15**, 50.0 mg, 144 µmol), K_2_CO_3_ (80.0 mg, 578 µmol) and the respective electrophiles (methyl iodide, allyl bromide, benzyl bromide, and methyl bromoacetate, 578 µmol each) were placed in DMF (3.00 mL) and stirred at 40°C for 20 h. The reaction mixture was quenched with ice. The precipitate was filtered off and purified by flash column chromatography on silica gel (cyclohexane/ethyl acetate, 9:1 → 1:1). The product was obtained as a yellow solid.

## Supporting Information

The Supporting Information includes the following chapters: experimental methods, synthesis and characterization, NMR spectra, photophysical characterization, molecular modeling, X‐ray single crystal structure analysis, precipitation of the E isomer of imide diazocine **9**. The authors have cited additional references within the Supporting Information.^[^ ^65^ ^]^

## Conflict of Interest

The authors declare no conflicts of interest.

## Supporting information



Supporting Information

## Data Availability

The data that support the findings of this study are available in the supplementary material of this article.
