# Supplementary material for: Synthesis and Properties of Cyclic Imide Extended Diazocines: Tweezer‐Like, Rigid Photoswitches with Large Switching Amplitudes
Source: Chemistry. 2025 Apr 3;31(29):e202500435. doi: 10.1002/chem.202500435 (PMC12099182; doi:10.1002/chem.202500435)
Supplement: Supplementary file 1 — Supporting Information [file CHEM-31-e202500435-s001.pdf]

Synthesis and Properties of Cyclic Imide Extended Diazocines: Tweezer-Like, Rigid Photoswitches With Large Switching Amplitudes

Artjom Businski[1], Thuy C. Ta[1], Lara Unterriker[1], Niklas Gindullis[2], Jan-Simon von Glasenapp[1], Christian Näther[3], Rainer Herges[1]

[1]Otto Diels Institute of Organic Chemistry, Kiel University, Kiel, Germany

[2]Institute of Organic Chemistry, Hannover University, Hannover, Germany

[3]Institute of Inorganic Chemistry, Kiel University, Kiel, Germany

E-mail: rherges@oc.uni-kiel.de

Table of Contents

S 1 Experimental methods 1

S 2 Syntheses and characterization 3
S 2.1 Synthesis of anhydride diazocine **7** 3
S 2.2 Synthesis of imide diazocines **8** – **19** 9

S 3 NMR spectra 22

S 4 Photophysical characterization 39
S 4.1 UV/vis absorption spectra and thermal half-lives 39
S 4.2 Photostationary states 54

S 5 Molecular modeling 69
S 5.1 Theoretical calculations of imide diazocines **9** and **15** 69
S 5.2 Calculations of the motion amplitudes of differently substituted diazocines 71

S 6 X-ray single crystal structure analysis 72

S 7 Precipitation of the *E* isomer of imide diazocine **9** 82

S 8 References 83

S 1 Experimental methods

General information

Unless otherwise stated, the syntheses were carried out at room temperature, under atmospheric pressure and atmospheric air. The water used was softened by an ion exchanger. Chemicals were purchased commercially and used without any changes.

NMR spectroscopy

FT NMR spectrometers: Bruker Avance Neo 500 (^{1}H NMR: 500.1 MHz, ^{13}C NMR: 125.8 MHz) and Bruker Avance 600 (^{1}H NMR: 600.1 MHz, ^{13}C NMR: 150.9 MHz).

Unless otherwise stated, the NMR spectra were recorded at 298 K. Two-dimensional NMR spectra (COSY, HSQC, HMBC) were additionally measured. For the chemical shift (δ in ppm), the signal of the solvent was used as a reference. For the multiplicities of the ^{1}H NMR signals the following abbreviations were used: s (singlet), br. s (broad singlet), d (doublet), t (triplet), m (multiplet) and m_c (multiplet centered). Deuterated solvents were purchased from Eurisotop®.

solvent	degree of deuteration / %	^{1}H NMR signal / ppm[1]	^{13}C NMR signal / ppm[1]
CD_2Cl_2	99.80	5.32	53.84
$CDCl_3$	99.80	7.26	77.16
DMSO-d_6	99.80	2.50	39.52
THF-d_8	99.50	3.58, 1.72	67.21, 25.31

Mass spectrometry

Joel AccuTOF 4G using electron ionization (EI) at an ionization energy of 70 eV and Thermo Scientific Q Exactive Plus using electrospray ionization (ESI).

IR spectroscopy

Perkin Elmer Spectrum100 using a Specac MKII Golden Gate Single Reflection Diamond ATR A531-G system. For the intensities of the absorption bands the following abbreviations were used: s (strong), m (medium), w (weak).

UV/vis spectroscopy

Shimadzu UV-2600i using a Lauda thermostat and Hellma high performance quartz glass cuvettes (optical path length of 10 mm, volume of 3500 µL).

Melting point determination

Büchi M-560 using melting point tubes. Melting points were only determined for crystalline products (except from clathrates, see chapter S 6).

Thin layer chromatography

Macherey-Nagel silica gel coated Xtra SIL G/UV254 ALUGRAM® plates using a CAMAG UV lamp at wavelengths of 254 nm and 366 nm.

Automatic flash column chromatography

Biotage® Isolera™ One 3.0 using Interchim puriFlash® cartridges (PF-30SIHP-F0012, PF-30SIHP-F0025, PF-30SIHP-F0040).

Light sources

Custom-made light sources with wavelengths of 365 nm, 385 nm, 405 nm, 420 nm and 530 nm (Sahlmann Photochemical Solutions).

S 2 Syntheses and characterization

S 2.1 Synthesis of anhydride diazocine 7

2-Nitro-4,5-dimethyl phthalate (2)

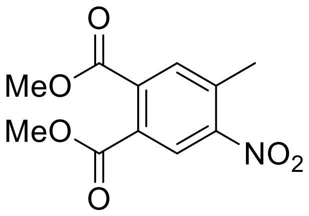

2

Caution: Large quantities of nitrous gases, hydrochloric acid and sulfur dioxide are produced during this reaction at this scale. Special attention must also be paid to the careful addition of the nitrating acid in order to maintain the required reaction temperature.

4-Methylphthalic anhydride (**1**, 250 g, 1.54 mol) was stirred at 80 °C in concentrated sulfuric acid (200 mL) until everything was completely dissolved. A mixture of fuming nitric acid (96.7 mL) and concentrated sulfuric acid (68.3 mL) was slowly added, so that the temperature of the reaction mixture did not exceed 110 °C. After complete addition, nitric acid (65 %, 412 mL) was immediately added. The reaction mixture was stirrred at 110 °C for 3 h and then quenched with ice. The precipitated wet solid was filtered off and dissolved in ethyl acetate using an ultrasonic bath. The aqueous phase formed was separated, the organic phase was washed three times with saturated sodium chloride solution (100 mL each), dried over magnesium sulfate, filtered off, and the solvent was removed in vacuo. The filtrate obtained after separation of the solid after quenching was additionally extracted three times with ethyl acetate (100 mL each), the combined organic phases were dried over magnesium sulfate, filtered off, and the solvent was removed in vacuo. The combined residues were additionally dried in vacuo at 70 °C. The crude product was then dissolved in dry methanol (2000 mL) and thionyl chloride (246 mL) was slowly added. The reaction mixture was stirred at 80 °C for 15 h and then quenched with ice. The precipitate was filtered off, dried in vacuo and recrystallized from ethanol. The product was obtained as a colorless solid.

Yield: 152 g (601 mmol, 39 %). (Lit.[2]: 43 %).

^{1}**H NMR** (600 MHz, $CDCl_3$): δ = 8.40 (s, 1H), 7.61 (br. s, 1H), 3.95 (s, 3H), 3.94 (s, 3H), 2.67 (br. s, 3H) ppm.

^{13}**C NMR** (151 MHz, $CDCl_3$): δ = 167.1, 165.3, 149.8, 137.6, 136.9, 133.2, 129.7, 126.0, 53.3, 53.2, 20.5 ppm.

MS (EI): *m/z* (%) = 253.06 (14) $[M]^{\cdot+}$, 236.06 (79), 222.04 (100), 205.04 (17), 176.05 (42), 117.03 (11), 90.05 (24).

HRMS (EI): $^{12}C_{11}{}^{1}H_{11}{}^{14}N_{1}{}^{16}O_{6}$ calc.: 253.05864, found: 253.05874.

IR (ATR): $\tilde{\nu}$ = 3049 (w), 2958 (w), 2166 (w), 1738 (m), 1716 (s), 1620 (m), 1575 (w), 1527 (m), 1435 (m), 1376 (m), 1353 (m), 1307 (s), 1259 (s), 1202 (s), 1136 (m), 1111 (m), 1037 (w), 1011 (m), 963 (m), 919 (m), 906 (m), 845 (m), 827 (m), 788 (m), 772 (m), 754 (m), 709 (m), 690 (m), 646 (w), 613 (w), 555 (m), 525 (w), 459 (w), 418 (w), 406 (w) cm^{-1}.

m.p.: 87 °C.

1,2-Bis(2-nitro-4,5-dimethyl phthalate)ethane (3)

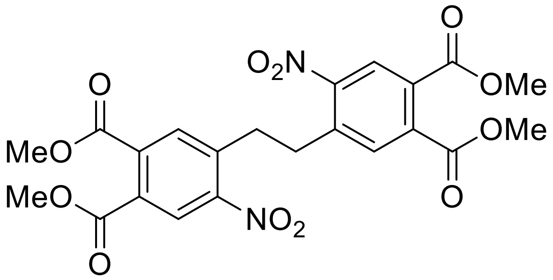

3

Under nitrogen atmosphere dimethyl-4-methyl-5-nitro phthalate (**2**, 10.0 g, 39.5 mmol) was dissolved in dry tetrahydrofuran (330 mL) and cooled to - 10 °C. Potassium *tert*-butoxide (5.76 g, 51.3 mmol) was added in one portion. The reaction mixture was stirred for 30 s, whereupon bromine (2.02 mL, 39.5 mmol) was immediatley added. After complete addition, the reaction mixture was stirred at - 10 °C for 10 min and then quenched with ice. The precipitate was filtered off, washed with the smallest possible amount of ice-cold ethyl acetate and dried in vacuo. The crude product was purified by flash column chromatography on silica gel (cyclohexane/ethyl acetate, 9:1 → 1:1 + 5 % methanol). The product was obtained as a pale yellow solid.

Yield: 5.37 g (10.6 mmol, 54 %).

^{1}H NMR (600 MHz, $CDCl_3$): δ = 8.41 (s, 2H), 7.70 (s, 2H), 3.97 (s, 6H), 3.96 (s, 6H), 3.31 (s, 4H) ppm.

^{13}C NMR (151 MHz, $CDCl_3$): δ = 166.7, 165.1, 149.8, 139.0, 137.4, 132.8, 130.7, 126.3, 53.5, 53.4, 34.2 ppm.

MS (ESI): *m/z* (%) = 505.11 (< 1) $[M+H]^+$, 432.20 (4), 274.11 (24), 257.08 (100), 229.11 (4).

HRMS (ESI): $C_{22}H_{21}N_2O_{12}$ calc.: 505.10890, found: 505.10776.

IR (ATR): $\tilde{\nu}$ = 3013 (w), 2956 (w), 2752 (w), 2166 (w), 1728 (s), 1616 (m), 1570 (m), 1544 (m), 1437 (m), 1390 (w), 1359 (m), 1301 (s), 1269 (s), 1233 (s), 1198 (s), 1181 (m), 1139 (s), 1110 (m), 1015 (m), 971 (m), 933 (m), 906 (s), 846 (m), 821 (m), 799 (m), 782 (s), 744 (w), 702 (w), 635 (m), 571 (m), 526 (w), 507 (w), 496 (w), 439 (w) 411 (m) cm^{-1}.

1,2-Bis(2-amino-4,5-dimethyl phthalate)ethane (4)

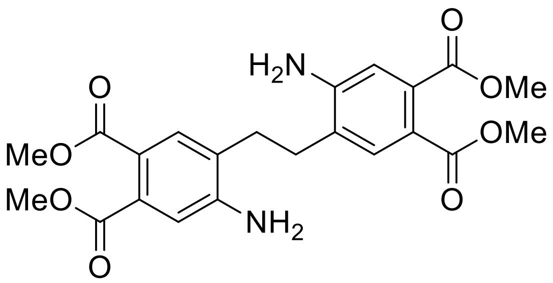

4

1,2-Bis(2-nitro-4,5-dimethyl phthalate)ethane (**3**, 3.00 g, 5.95 mmol) was suspended in a mixture consisting of ethyl acetate (150 mL) and hydrochloric acid (37 %, 18.6 mL) and stirred at room temperature. Zinc powder (4.67 g, 71.4 mmol) was added in small portions. After complete addition, the reaction mixture was stirred at room temperature for 2 h. The precipitate was filtered off and mixed with ethyl acetate and sodium hydroxide solution (25 wt%) under ice cooling until the precipitate was completely dissolved. The phases were separated, the aqueous phase was extracted three times with ethyl acetate (50.0 mL each), the combined organic phases were dried over magnesium sulfate, filtered off, and the solvent was removed in vacuo. The crude

product was purified by flash column chromatography on silica gel (cyclohexane/ethyl acetate, 3:1 → 0:1). The product was obtained as a pale yellow solid.

Yield: 1.64 g (3.69 mmol, 62 %).

^{1}H NMR (500 MHz, DMSO-d_6): δ = 7.52 (s, 2H), 6.71 (s, 2H), 6.00 (s, 4H), 3.75 (s, 6H), 3.70 (s, 6H), 2.69 (s, 4H) ppm.

^{13}C NMR (126 MHz, DMSO-d_6): δ = 169.3, 166.4, 150.2, 133.8, 131.1, 125.2, 114.9, 112.7, 52.2, 51.7, 28.6 ppm.

MS (EI): *m/z* (%) = 444.14 (4) $[M]^{\cdot+}$, 412.11 (22), 365.06 (3), 326.12 (10), 294.09 (5), 250.10 (2), 222.07 (100), 194.08 (25), 176.03 (9), 148.04 (2), 133.05 (16), 105.05 (39).

HRMS (EI): $^{12}C_{22}{}^{1}H_{24}{}^{14}N_{2}{}^{16}O_{8}$ calc.: 444.15326, found: 444.15323.

IR (ATR): $\tilde{\nu}$ = 3460 (w), 3369 (m), 3262 (w), 2947 (w), 2253 (w), 2167 (w), 2011 (w), 1732 (m), 1694 (s), 1652 (m), 1622 (m), 1602 (m), 1568 (m), 1515 (w), 1433 (m), 1325 (m), 1276 (s), 1248 (s), 1185 (s), 1119 (s), 1044 (s), 965 (m), 909 (m), 882 (m), 852 (w), 798 (m), 783 (m), 764 (m), 674 (w), 558 (m), 476 (m), 450 (m), 411 (s) cm^{-1}.

2,3,8,9-Tetramethylcarboxylate-11,12-dihydrodibenzo[*c,g*][1,2]diazocine (5)

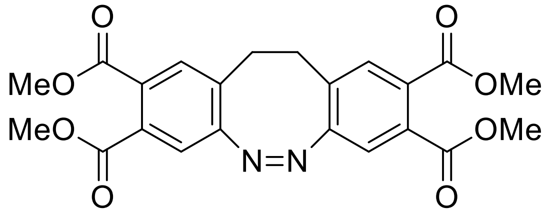

5

1,2-Bis(2-amino-4,5-dimethyl phthalate)ethane (**4**, 1.44 g, 3.24 mmol) was suspended in a mixture consisting of toluene and concentrated acetic acid (3:1, 80 mL) and stirred until a temperature of 80 °C was reached. Subsequently, under stirring at 80 °C, a solution of *meta*-chloroperoxybenzoic acid (1.45 g, 8.42 mmol) in concentrated acetic acid (60.0 mL) was added dropwise over a period of 24 h using a syringe pump. The solvent was removed in vacuo and the residue was dissolved in ethyl acetate (80.0 mL). The organic phase was washed three times with a saturated sodium bicarbonate solution (50.0 mL each), dried over magnesium sulfate, filtered off, and the solvent was removed in vacuo. The crude product was purified by flash column

chromatography on silica gel (cyclohexane/ethyl acetate, 3:1 → 1:1). The rack with the filled test tubes was placed in the refrigerator until a yellow precipitate formed, which was filtered off and dried in vacuo. The filtrate was combined together with the remaining product fraction and the solvent was removed in vacuo. The residue was recrystallized stepwise from cyclohexane/ethyl acetate (3:1) until no more yellow solid was formed. The filtered solids were combined and dried in vacuo. The product was obtained as a yellow solid.

Yield: 669 mg (1.52 mmol, 47 %).

^{1}H NMR (600 MHz, $CDCl_3$): δ = 7.38 (s, 2H), 7.27 (s, 2H), 3.87 (s, 6H), 3.85 (s, 6H), 3.08 – 2.84 (m_c, 4H) ppm.

^{13}C NMR (151 MHz, $CDCl_3$): δ = 167.3, 166.7, 156.5, 131.4, 131.1, 130.98, 130.8, 120.3, 52.94, 52.90, 31.4 ppm.

MS (EI): *m/z* (%) = 440.12 (8) $[M]^{\cdot+}$, 381.09 (89), 353.10 (6), 334.97 (26), 321.07 (7), 284.97 (16), 263.07 (12), 235.07 (3), 176.06 (19), 168.99 (100), 146.98 (30), 118.99 (28).

HRMS (EI): $^{12}C_{22}{}^{1}H_{20}{}^{14}N_2{}^{16}O_8$ calc.: 440.12196, found: 440.12176.

IR (ATR): $\tilde{\nu}$ = 2953 (w), 1715 (s), 1607 (w), 1558 (w), 1489 (w), 1433 (m), 1384 (w), 1296 (s), 1254 (s), 1199 (s), 1120 (s), 1035 (m), 996 (w), 960 (m), 903 (m), 830 (m), 790 (m), 765 (m), 702 (w), 669 (m), 634 (w), 545 (w), 520 (w), 506 (w), 486 (w), 412 (m) cm^{-1}.

m.p.: 173 °C.

2,3,8,9-Tetracarboxylic acid-11,12-dihydrodibenzo[*c,g*][1,2]diazocine (6)

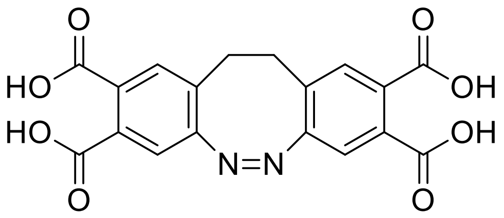

6

2,3,8,9-Tetramethylcarboxylate-11,12-dihydrodibenzo[*c,g*][1,2]diazocine (**5**, 100 mg, 227 µmol) was dissolved in dimethylformamide (6.00 mL) at room temperature. Subsequently, a solution of sodium hydroxide (30 wt%, 1.00 mL) was added and the reaction mixture was stirred at room temperature for 90 min. The reaction mixture was quenched with ice and acidified with hydrochloric acid (2 M) until pH = 1 was reached. The aqueous phase was extracted twice with dichloromethane (25.0 mL each), whereupon the organic phase was extracted twice with water (50.0 mL each) again. The organic phase consisting of dichloromethane was disposed. A saturated sodium chloride solution (50.0 mL) was added to the combinded aqueous phase, which was extracted five times with ethyl acetate (50.0 mL each). The combinded organic phase was dried over magnesium sulfate, filtered off, and the solvent was removed in vacuo. The waxy residue was placed in the refrigerator until a yellow precipitate formed. A small amount of dichloromethane was added and sonicated until a suspension has formed. The precipitate was filtered off and dried in vacuo. The product was obtained as a yellow solid.

Yield: 73.4 mg (191 µmol, 84 %).

^{1}H NMR (500 MHz, THF-*d$_8$*): δ = 11.6 (br. s, 4H), 7.46 (s, 2H), 7.26 (s, 2H), 3.02 – 2.95 (m_c, 4H) ppm.

^{13}C NMR (126 MHz, THF-*d$_8$*): δ = 168.1, 167.8, 157.5, 133.2, 133.1, 131.9, 131.4, 120.9, 32.1 ppm.

MS (ESI): *m/z* (%) = 383.05 (100) $[M-H]^-$, 253.99 (2), 191.02 (4).

HRMS (ESI): $C_{18}H_{11}N_2O_8$ calc.: 383.05209, found: 383.05165.

IR (ATR): $\tilde{\nu}$ = 2957 (m), 1726 (s), 1685 (s), 1606 (w), 1560 (m), 1489 (w), 1458 (w), 1425 (m), 1393 (m), 1366 (m), 1344 (m), 1244 (s), 1201 (s), 1147 (m), 1123 (s), 1058 (m), 1031 (s), 921 (s), 853 (m), 802 (m), 772 (m), 708 (m), 630 (m), 578 (w), 542 (w), 506 (w), 462 (w), 439 (w), 428 (w), 412 (m) cm^{-1}.

2,3,8,9-Bissuccinic anhydride-11,12-dihydrodibenzo[*c,g*][1,2]diazocine (7)

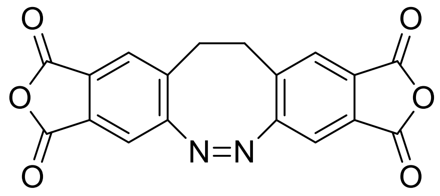

7

2,3,8,9-Tetracarboxylic acid-11,12-dihydrodibenzo[*c,g*][1,2]diazocine (**6**, 50.0 mg, 130 µmol) was dissolved in acetic anhydride (25.0 mL) and stirred at 120 °C for 2 h. The solvent was removed in vacuo. The crude product was obtained as a yellow solid and used without further purification.

S 2.2 Synthesis of imide diazocines 8 – 19

General procedure for the synthesis of substituted imide diazocines 8 – 14 starting from tetraester diazocine 5

2,3,8,9-Tetramethylcarboxylate-11,12-dihydrodibenzo[*c,g*][1,2]diazocine (**5**, 200 mg, 454 µmol) was dissolved in dimethylformamide (12.0 mL) at room temperature. Subsequently, a solution of sodium hydroxide (30 wt%, 2.00 mL) was added and the reaction mixture was stirred at room temperature for 90 min. The reaction mixture was quenched with ice and acidified with hydrochloric acid (2 M) until pH = 1 was reached. The aqueous phase was extracted twice with dichloromethane (50.0 mL each), whereupon the organic phase was extracted twice with water (100 mL each) again. The organic phase consisting of dichloromethane was disposed. A saturated sodium chloride solution (100 mL) was added to the combinded aqueous phase, which was extracted five times with ethyl acetate (100 mL each). The combinded organic phase was dried over magnesium sulfate, filtered off, and the solvent was removed in vacuo. The resulting residue (crude product that mainly contains tetracarboxylic acid diazocine **6**) was dissolved in acetic anhydride (50.0 mL) and stirred at 120 °C for 2 h. The solvent was removed in vacuo. The resulting residue (crude product that mainly contains anhydride diazocine **7**) and the corresponding amine (908 µmol) were

dissolved in concentrated acetic acid (45.0 mL) and stirred at 120 °C for 20 h. The reaction mixture was quenched with ice.

Purification method A:

The aqueous phase was extracted three times with dichloromethane (50.0 mL each), the combinded organic phase was washed with saturated sodium bicarbonate solution (50.0 mL), dried over magnesium sulfate, filtered off, and the solvent was removed in vacuo. The crude product was purified by flash column chromatography on silica gel (cyclohexane/ethyl acetate, 9:1 → 1:1). The product was obtained as a yellow solid.

Purification method B:

The precipitate was filtered off and purified by flash column chromatography on silica gel (dichloromethane/methanol, 1:0 → 9:1). The product was obtained as a yellow solid.

Purification method C:

The precipitate was filtered off and purified by flash column chromatography on silica gel (cyclohexane/ethyl acetate, 3:1 → 1:1). The product was obtained as a yellow solid.

Purification method D:

The precipitate was filtered off, treated with a small amount of ethanol and sonicated for 15 min. The solid was filtered off and dried in vacuo. The product was obtained as a yellow solid.

***N,N'*-2,3,8,9-Bispropylsuccinimide-11,12-dihydrodibenzo[*c,g*][1,2]diazocine (8)**

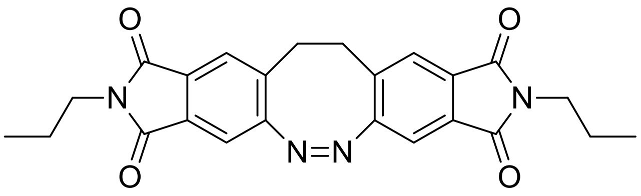

8

Propylamine (53.7 mg, 908 µmol), purification method A.

Yield: 97.7 mg (227 µmol, 50 %).

^{1}H NMR (600 MHz, CD_2Cl_2): δ = 7.46 (s, 2H), 7.25 (s, 2H), 3.54 – 3.52 (m, 4H), 3.13 – 3.00 (m_c, 4H), 1.60 (sextet, J = 7.4 Hz, 4H), 0.87 (t, J = 7.4 Hz, 6H) ppm.

^{13}C NMR (151 MHz, CD_2Cl_2): δ = 167.7, 167.4, 159.8, 134.6, 132.0, 131.5, 125.3, 113.7, 40.1, 32.0, 22.2, 11.4 ppm.

MS (EI): *m/z* (%) = 430.18 (7) $[M]^{\cdot+}$, 401.14 (4), 373.13 (100), 331.09 (17), 317.12 (13), 260.08 (13), 247.07 (3), 204.08 (7), 176.07 (15), 102.04 (5).

HRMS (EI): $^{12}C_{24}{}^{1}H_{22}{}^{14}N_4{}^{16}O_4$ calc.: 430.16410, found: 430.16390.

IR (ATR): $\tilde{\nu}$ = 3032 (w), 2967 (w), 2875 (w), 2324 (w), 2169 (w), 2049 (w), 1986 (w), 1769 (m), 1702 (s), 1619 (m), 1435 (m), 1394 (s), 1365 (s), 1345 (m), 1309 (m), 1178 (m), 1141 (w), 1073 (s), 1025 (m), 930 (m), 888 (m), 859 (m), 782 (m), 741 (s), 678 (w), 636 (w), 610 (m), 577 (m), 546 (w), 511 (w), 478 (m), 436 (w), 420 (m), 403 (m) cm^{-1}.

N,N'-2,3,8,9-Bisphenylsuccinimide-11,12-dihydrodibenzo[*c,g*][1,2]diazocine (9)

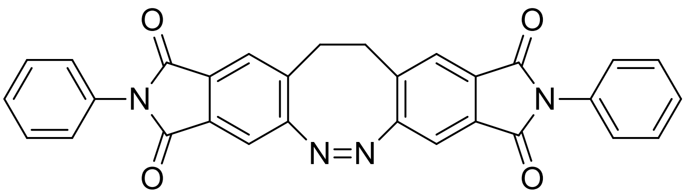

9

Aniline (84.6 mg, 908 µmol), purification method B.

Yield: 154 mg (309 µmol, 68 %).

^{1}H NMR (600 MHz, CD_2Cl_2): δ = 7.61 (s, 2H), 7.50 – 7.47 (m, 4H), 7.42 – 7.39 (m, 4H), 7.34 – 7.32 (m, 4H), 3.21 – 3.09 (m_c, 4H) ppm.

^{13}C NMR (151 MHz, CD_2Cl_2): δ = 166.5, 166.3, 160.3, 135.4, 132.0, 131.6, 131.1, 129.5, 128.7, 127.0, 125.9, 114.2, 32.0 ppm.

MS (EI): *m/z* (%) = 498.13 (40) $[M]^{\cdot+}$, 470.12 (100), 426.13 (58), 381.13 (26), 351.09 (34), 323.09 (31), 305.10 (18), 278.09 (40), 251.08 (12), 230.06 (7), 203.07 (19), 176.06 (42), 150.04 (13), 126.04 (6), 91.04 (11).

HRMS (EI): $^{12}C_{30}{}^{1}H_{18}{}^{14}N_{4}{}^{16}O_{4}$ calc.: 498.13280, found: 498.13285.

IR (ATR): $\tilde{\nu}$ = 1775 (m), 1711 (s), 1618 (w), 1593 (w), 1491 (m), 1455 (w), 1430 (w), 1369 (s), 1255 (w), 1235 (w), 1170 (w), 1112 (m), 1100 (m), 1070 (m), 932 (m), 909 (w), 897 (w), 862 (w), 804 (w), 791 (w), 748 (m), 689 (m), 644 (w), 620 (m), 579 (w), 545 (w), 509 (m), 480 (m), 420 (w) cm^{-1}.

N,N'-**2,3,8,9-Bis-(4-methoxyphenyl)succinimide-11,12-dihydrodibenzo[*c,g*][1,2]-diazocine (10)**

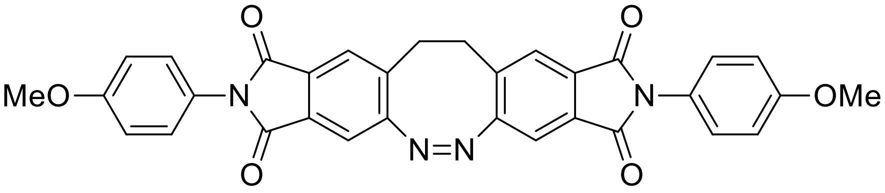

10

4-Methoxyaniline (112 mg, 908 µmol), purification method C.

Yield: 25.4 mg (45.4 µmol, 10 %).

^{1}H NMR (600 MHz, CD_2Cl_2): δ = 7.59 (s, 2H), 7.37 (s, 2H), 7.23 – 7.22 (m, 4H), 6.99 – 6.98 (m, 4H), 3.83 (s, 6H), 3.20 – 3.07 (m_c, 4H) ppm.

^{13}C NMR (151 MHz, CD_2Cl_2): δ = 166.8, 166.6, 160.3, 159.9, 135.3, 131.7, 131.2, 128.3, 125.8, 124.5, 114.7, 114.1, 55.9, 32.0 ppm.

MS (EI): *m/z* (%) = 558.18 (100) $[M]^{\cdot+}$, 530.17 (25), 515.15 (43), 487.16 (8), 471.15 (6), 380.13 (9), 353.12 (22), 338.10 (8), 309.12 (4), 279.09 (9), 230.07 (5), 204.07 (7), 180.99 (12), 168.99 (16), 150.05 (4), 130.99 (26), 118.99 (34).

HRMS (EI): $^{12}C_{32}{}^{1}H_{22}{}^{14}N_{4}{}^{16}O_{4}$ calc.: 558.15393, found: 558.15365.

IR (ATR): $\tilde{\nu}$ = 2938 (w), 2325 (w), 2164 (w), 2037 (w), 1979 (w), 1777 (m), 1712 (s), 1607 (m), 1588 (m), 1510 (s), 1463 (m), 1442 (m), 1380 (s), 1303 (m), 1244 (s), 1170 (m), 1117 (m), 1102 (s), 1027 (s), 965 (w), 907 (m),869 (m), 842 (m), 787 (s), 741 (s), 720 (s), 639 (m), 615 (m), 597 (m), 572 (m), 527 (m), 488 (m), 459 (m), 421 (m) cm^{-1}.

N,N'-2,3,8,9-Bis-(4-iodophenyl)succinimide-11,12-dihydrodibenzo[*c,g*][1,2]-diazocine (11)

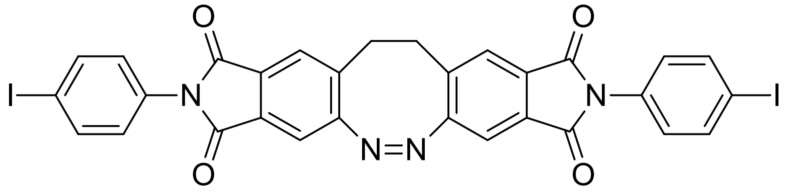

11

4-Iodoaniline (199 mg, 908 µmol), purification method C.

Yield: 27.2 mg (36.3 µmol, 8 %).

^{1}H NMR (600 MHz, CD_2Cl_2): δ = 7.82 – 7.80 (m, 4H), 7.60 (s, 2H), 7.39 (s, 2H), 7.14 – 7.12 (m, 4H), 3.20 – 3.08 (m_c, 4H) ppm.

^{13}C NMR (151 MHz, CD_2Cl_2): δ = 166.1, 165.9, 160.4, 138.6, 135.6, 131.8, 131.5, 130.9, 128.4, 126.0, 114.3, 93.6, 32.0 ppm.

MS (EI): *m/z* (%) = 749.97 (82) $[M]^{\cdot+}$, 721.96 (100), 677.96 (22), 551.05 (32), 477.01 (26), 449.01 (28), 405.02 (12), 377.00 (15), 278.11 (30), 204.07 (16), 176.07 (41), 127.92 (17), 90.04 (27).

HRMS (EI): $^{12}C_{30}{}^{1}H_{16}{}^{127}I_2{}^{14}N_4{}^{16}O_4$ calc.: 749.92609, found: 749.92630.

IR (ATR): $\tilde{\nu}$ = 3479 (w), 3101 (w), 2924 (w), 2324 (w), 2195 (w), 2164 (w), 1778 (m), 1715 (s), 1620 (w), 1485 (s), 1370 (s), 1237 (m), 1175 (w), 1105 (m), 1085 (m), 1057 (m), 1005 (m), 925 (m), 862 (m), 828 (m), 813 (m), 786 (s), 734 (s), 696 (m), 652 (m), 638 (m), 615 (m), 571 (m), 505 (s), 481 (m), 445 (w) cm^{-1}.

N,N'-2,3,8,9-Bis-(4-carboxylic acidphenyl)succinimide-11,12-dihydrodibenzo[*c,g*][1,2]-diazocine (12)

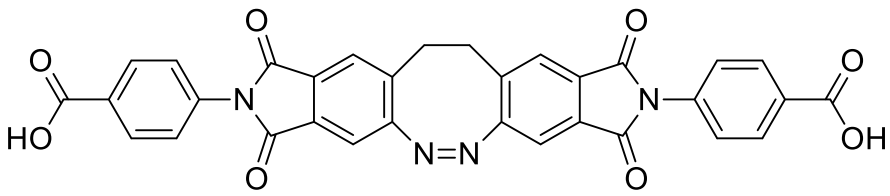

12

4-Aminobenzoic acid (125 mg, 908 µmol), purification method D.

Yield: 103 mg (177 µmol, 39 %).

^{1}H NMR (600 MHz, DMSO-*d*$_6$ + TFA-*d*): *δ* = 8.05 – 8.04 (m, 4H), 7.82 (s, 2H), 7.61 (s, 2H), 7.53 – 7.52 (m, 4H), 3.26 – 3.04 (m$_c$, 4H) ppm.

TFA-*d* was used to avoid superposition of the water signal at 3.30 ppm with the signal at 3.26 – 3.04 ppm. As a result, the signal of the carboxylic acid proton is not detectable.

^{13}C NMR (151 MHz, DMSO-*d*$_6$ + TFA-*d*): *δ* = 166.7, 165.9, 165.7, 159.5, 135.7, 135.2, 130.9, 130.4, 130.0, 129.9, 126.9, 125.8, 114.0, 30.8 ppm.

MS (EI): *m/z* (%) = 586.14 (36) [M]$^{\cdot+}$, 542.14 (37), 498.15 (23), 454.16 (4), 394.10 (5), 350.11 (4), 267.09 (100), 251.09 (73), 203.07 (8), 168.99 (11), 131.00 (15), 118.99 (18), 104.05 (43).

HRMS (EI): $^{12}C_{32}{}^{1}H_{18}{}^{14}N_4{}^{16}O_8$ calc.: 586.11246, found: 586.11261.

IR (ATR): $\tilde{\nu}$ = 2954 (w), 2662 (w), 1781 (m), 1724 (s), 1689 (s), 1607 (m), 1512 (m), 1423 (m), 1348 (s), 1286 (s), 1185 (m), 1125 (m), 1067 (m), 1016 (m), 930 (m), 859 (m), 788 (m), 765 (s), 739 (s), 714 (m), 692 (m), 637 (w), 616 (m), 544 (m), 507 (m), 479 (w) 458 (w), 431 (m) cm^{-1}.

***N,N'*-2,3,8,9-Bis-(4-nitrophenyl)succinimide-11,12-dihydrodibenzo[*c,g*][1,2]-diazocine (13)**

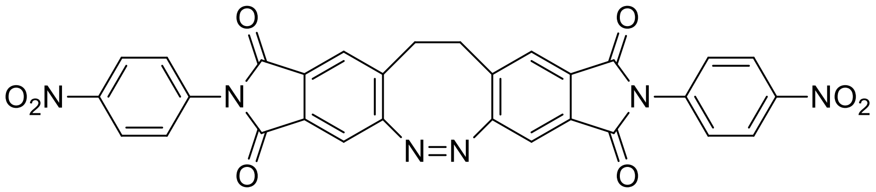

13

4-Nitroaniline (125 mg, 908 µmol), purification method D.

Yield: 48.1 mg (81.7 µmol, 18 %).

^{1}H NMR (600 MHz, DMSO-*d*$_6$ + TFA-*d*): *δ* = 8.37 – 8.34 (m, 4H), 7.61 (s, 2H), 7.72 – 7.70 (m, 4H), 7.64 (s, 2H), 3.29 – 3.02 (m$_c$, 4H) ppm.

TFA-*d* was used to avoid superposition of the water signal at 3.30 ppm with the signal at 3.29 – 3.02 ppm.

^{13}C NMR (151 MHz, DMSO-d_6 + TFA-*d*): δ = 165.6, 165.5, 159.6, 146.3, 137.7, 135.5, 130.8, 130.4, 127.6, 126.0, 124.3, 114.1, 30.9 ppm.

MS (EI): *m/z* (%) = 588.16 (37) $[M]^{\cdot+}$, 560.15 (100), 530.15 (62), 516.16 (13), 470.16 (50), 422.12 (7), 396.11 (48), 368.12 (35), 322.12 (13), 293.11 (9), 278.12 (29), 230.08 (8), 204.08 (33), 176.08 (71), 150.06 (18), 91.05 (16).

HRMS (EI): $^{12}C_{30}{}^{1}H_{16}{}^{14}N_4{}^{16}O_8$ calc.: 588.10296, found: 588.10266.

IR (ATR): $\tilde{\nu}$ = 2965 (w), 1782 (m), 1717 (s), 1595 (m), 1518 (m), 1496 (m), 1379 (m), 1338 (s), 1118 (m), 1102 (m), 1013 (w), 955 (w), 885 (w), 843 (m), 798 (m), 743 (s), 683 (m), 663 (w), 649 (w), 630 (w), 611 (m), 578 (w), 553 (w), 518 (w), 502 (m), 431 (m) cm^{-1}.

N,N'-2,3,8,9-Bis-(3-hydroxyphenyl)succinimide-11,12-dihydrodibenzo[*c,g*][1,2]-diazocine (14)

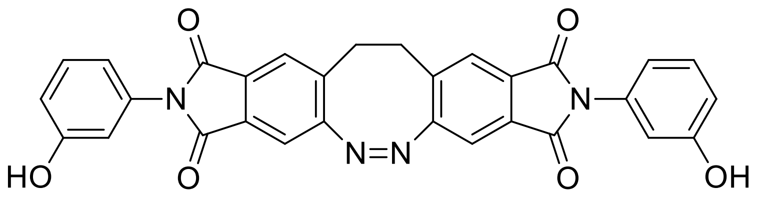

14

3-Hydroxyaniline (99.1 mg, 908 µmol), purification method C.

Yield: 28.9 mg (54.5 µmol, 12 %).

^{1}H NMR (600 MHz, DMSO-d_6 + TFA-*d*): δ = 7.77 (s, 2H), 7.56 (s, 2H), 7.26 – 7.23 (m, 2H), 6.81 – 6.79 (m, 2H), 6.78 – 6.76 (m, 4H), 3.26 – 2.99 (m_c, 4H) ppm.

TFA-*d* was used to avoid superposition of the water signal at 3.30 ppm with the signal at 3.26 – 2.99 ppm. As a result, the signal of the hydroxyl proton is not detectable.

^{13}C NMR (151 MHz, DMSO-d_6 + TFA-*d*): δ = 166.2, 166.0, 159.4, 157.6, 135.0, 132.7, 130.9, 130.5, 129.6, 125.6, 117.8, 115.2, 114.3, 113.8, 30.8 ppm.

MS (EI): *m/z* (%) = 530.17 (80) $[M]^{·+}$, 502.16 (100), 474.16 (7), 458.17 (44), 413.16 (16), 367.12 (51), 339.12 (49), 322.13 (20), 295.12 (36), 267.11 (15), 230.09 (12), 204.08 (31), 176.08 (70), 150.06 (23), 109.06 (20), 92.03 (69).

HRMS (EI): $^{12}C_{30}{}^{1}H_{18}{}^{14}N_{4}{}^{16}O_{6}$ calc.: 530.12263, found: 530.12275.

IR (ATR): $\tilde{\nu}$ = 3462 (w), 2925 (w), 2845 (w), 2325 (w), 2252 (w), 2194 (w), 2163 (w), 1773 (m), 1704 (s), 1604 (m), 1508 (m), 1458 (m), 1383 (s), 1272 (m), 1214 (m), 1156 (m), 1104 (m), 1072 (m), 1033 (w), 1002 (w), 949 (w), 922 (w), 862 (m), 798 (m), 779 (m), 741 (m), 713 (w), 685 (m), 619 (m), 572 (m), 548 (m), 471 (m), 438 (w), 421 (w) cm^{-1}.

2,3,8,9-Bissuccinimide-11,12-dihydrodibenzo[*c,g*][1,2]diazocine (15)

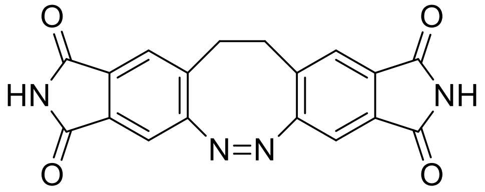

15

Procedure A:

2,3,8,9-Tetramethylcarboxylate-11,12-dihydrodibenzo[*c,g*][1,2]diazocine (**5**, 100 mg, 227 µmol) was dissolved in dimethylformamide (6.00 mL) at room temperature. Subsequently, a solution of sodium hydroxide (30 wt%, 1.00 mL) was added and the reaction mixture was stirred at room temperature for 90 min. The reaction mixture was quenched with ice and acidified with hydrochloric acid (2 M) until pH = 1 was reached. The aqueous phase was extracted twice with dichloromethane (25.0 mL each), whereupon the organic phase was extracted twice with water (50.0 mL each) again. The organic phase consisting of dichloromethane was disposed. A saturated sodium chloride solution (50.0 mL) was added to the combinded aqueous phase, which was extracted five times with ethyl acetate (50.0 mL each). The combinded organic phase was dried over magnesium sulfate, filtered off, and the solvent was removed in vacuo. The resulting residue (crude product that mainly contains tetracarboxylic acid diazocine **6**) was dissolved in acetic anhydride (25.0 mL) and stirred at 120 °C for 2 h. The solvent was removed in vacuo. The resulting residue (crude product that mainly

contains anhydride diazocine **7**) was treated with urea (82.0 mg, 1.37 mmol) in dry toluene (10.0 mL) and stirred at 120 °C for 3 h. The solvent was removed in vacuo and the crude product was purified by flash column chromatography on silica gel (cyclohexane/ethyl acetate, 3:1 → 1:1). The product was obtained as a yellow solid.

Yield: 18.9 mg (54.5 µmol, 24 %).

Procedure B:

2,3,8,9-Tetramethylcarboxylate-11,12-dihydrodibenzo[*c,g*][1,2]diazocine (**5**, 100 mg, 227 µmol) was dissolved in dimethylformamide (6.00 mL) at room temperature. Subsequently, a solution of sodium hydroxide (30 wt%, 1.00 mL) was added and the reaction mixture was stirred at room temperature for 90 min. The reaction mixture was quenched with ice and acidified with hydrochloric acid (2 M) until pH = 1 was reached. The aqueous phase was extracted twice with dichloromethane (25.0 mL each), whereupon the organic phase was extracted with water (50.0 mL each) again. The organic phase consisting of dichloromethane was disposed. A saturated sodium chloride solution (50.0 mL) was added to the combinded aqueous phase, which was extracted five times with ethyl acetate (50.0 mL each). The combinded organic phase was dried over magnesium sulfate, filtered off, and the solvent was removed in vacuo. The resulting residue (crude product that mainly contains tetracarboxylic acid diazocine **6**), urea (55.0 mg, 908 µmol) and imidazole (46.0 mg, 681 µmol) were dissolved in dimethylformamid (10.0 mL) and stirred at 150 °C for 3 h. The reaction mixture was treated with hydrochloric acid (2 M, 20.0 mL), filtered off, and the aqueous phase was extracted five times with ethyl acetate (50.0 mL each). The combined organic phase was dried over magnesium sulfate, filtered off, and the solvent was removed in vacuo. The crude product was purified by flash column chromatography on silica gel (cyclohexane/ethyl acetate, 3:1 → 1:1). The product was obtained as a yellow solid.

Yield: 53.3 mg (154 µmol, 68 %).

^{1}H NMR (600 MHz, DMSO-d_6): δ = 11.3 (br. s, 2H), 7.60 (s, 2H), 7.39 (br. s, 2H), 3.19 – 3.11 (m, 2H), 3.01 – 2.92 (m, 2H) ppm.

^{13}C NMR (151 MHz, DMSO-d_6): δ = 168.6, 168.4, 159.0, 134.5, 132.0, 131.6, 124.9, 113.1, 30.6 ppm.

MS (EI): *m/z* (%) = 346.09 (15) $[M]^{\cdot+}$, 318.08 (12), 290.08 (10), 275.07 (49), 247.07 (67), 204.07 (42), 176.07 (100), 102.03 (17).

HRMS (EI): $^{12}C_{18}{}^{1}H_{10}{}^{14}N_4{}^{16}O_4$ calc.: 346.07020, found: 346.07026.

IR (ATR): $\tilde{\nu}$ = 3325 (m), 1776 (m), 1704 (s), 1596 (m), 1507 (m), 1456 (m), 1380 (s), 1313 (m), 1267 (s), 1217 (m), 1109 (s), 1094 (s), 1001 (m), 968 (w), 935 (m), 888 (w), 866 (w), 846 (m), 779 (s), 744 (s), 708 (m), 684 (m), 617 (s), 537 (m), 472 (m), 460 (m), 407 (m) cm^{-1}.

General procedure for the synthesis of substituted imide diazocines 16 – 19 starting from parent imide diazocine 15

2,3,8,9-Bissuccinimide-11,12-dihydrodibenzo[*c,g*][1,2]diazocine (**15**, 50.0 mg, 144 µmol), potassium carbonate (80.0 mg, 578 µmol) and the corresponding halide (578 µmol) were placed in dimethylformamide (3.00 mL) and stirred at 40 °C for 20 h. The reaction mixture was quenched with ice. The precipitate was filtered off and purified by flash column chromatography on silica gel (cyclohexane/ethyl acetate, 9:1 → 1:1). The product was obtained as a yellow solid.

N,N'-2,3,8,9-Bismethylsuccinimide-11,12-dihydrodibenzo[*c,g*][1,2]diazocine (16)

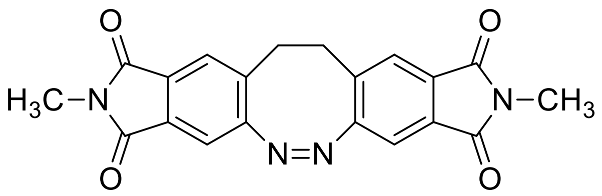

16

Methyl iodide (50.0 mg, 578 µmol).

Yield: 33.4 mg (89.3 µmol, 62 %).

^{1}H NMR (600 MHz, CD_2Cl_2): δ = 7.47 (s, 2H), 7.26 (s, 2H), 3.13 – 3.10 (m, 2H), 3.05 (s, 6H), 3.03 – 3.00 (m, 2H) ppm.

^{13}C NMR (151 MHz, CD_2Cl_2): δ = 167.6, 167.4, 159.8, 134.6, 132.1, 131.6, 125.3, 113.8, 32.0, 24.2 ppm.

MS (EI): *m/z* (%) = 374.10 (26) $[M]^{·+}$, 346.09 (44), 318.08 (17), 302.10 (60), 289.07 (62), 261.08 (63), 233.07 (10), 217.09 (28), 204.06 (55), 176.06 (100), 116.03 (10), 102.03 (19).

HRMS (EI): $^{12}C_{20}{}^{1}H_{14}{}^{14}N_4{}^{16}O_4$ calc.: 374.10150, found: 374.10135.

IR (ATR): $\tilde{\nu}$ = 2950 (w), 2844 (w), 1772 (m), 1704 (s), 1618 (w), 1429 (s), 1372 (s), 1275 (w), 1259 (m), 1140 (w), 1066 (m), 1033 (w), 998 (m), 979 (m), 930 (w), 916 (w), 903 (w), 837 (w), 764 (w), 742 (s), 606 (m), 492 (w) cm^{-1}.

m.p.: The melting point could not be determined due to decomposition above 220 °C.

N,N'-2,3,8,9-Bisbenzylsuccinimide-11,12-dihydrodibenzo[*c,g*][1,2]-diazocine (17)

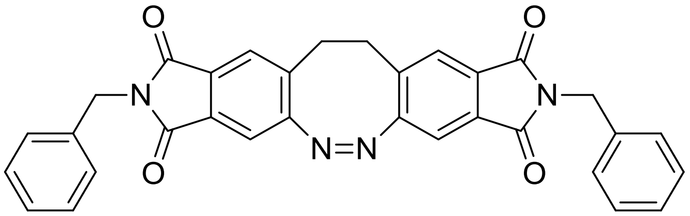

17

Benzyl bromide (99.0 mg, 578 µmol).

Yield: 35.6 mg (67.7 µmol, 47 %).

^{1}H NMR (600 MHz, CD_2Cl_2): δ = 7.45 (s, 2H), 7.32 – 7.30 (m, 4H), 7.28 – 7.23 (m, 8H), 4.72 (s, 4H), 3.11 – 2.98 (m, 4H) ppm.

^{13}C NMR (151 MHz, CD_2Cl_2): δ = 167.2, 167.0, 159.9, 136.8, 134.8, 132.0, 131.5, 129.0, 128.8, 128.2, 125.4, 113.8, 42.0, 31.0 ppm.

MS (EI): *m/z* (%) = 526.19 (12) $[M]^{·+}$, 498.18 (15), 480.17 (17), 470.18 (12), 410.12 (3), 365.12 (17), 337.12 (7), 261.09 (8), 205.07 (7), 176.07 (12), 104.05 (23), 91.06 (100).

HRMS (EI): $^{12}C_{32}{}^{1}H_{22}{}^{14}N_4{}^{16}O_4$ calc.: 526.16410, found: 526.16396.

IR (ATR): $\tilde{\nu}$ = 3033 (w), 2926 (w), 2854 (w), 2253 (w), 2193 (w), 1771 (m), 1708 (s), 1620 (w), 1495 (w), 1456 (w), 1429 (m), 1385 (s), 1338 (m), 1311 (m), 1288 (m), 1239 (m), 1106 (m), 1073 (m), 960 (w), 902 (m), 823 (w), 768 (m), 741 (s), 701 (s), 621 (m), 608 (m), 578 (m), 511 (m), 502 (m), 477 (m), 429 (m), 404 (m) cm^{-1}.

N,N'-2,3,8,9-Bisallylsuccinimide-11,12-dihydrodibenzo[*c,g*][1,2]diazocine (18)

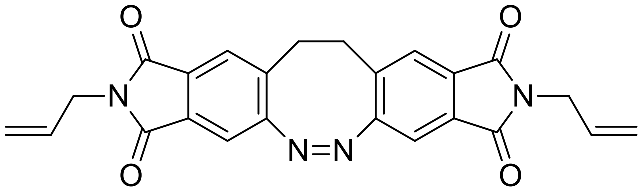

18

Allyl bromide (70.0 mg, 578 µmol).

Yield: 44.8 g (105 µmol, 73 %).

^{1}H NMR (600 MHz, CD_2Cl_2): δ = 7.48 (s, 2H), 7.27 (s, 2H), 5.85 – 5.77 (m, 2H), 5.17 – 5.11 (m, 4H), 4.19 – 4.17 (m, 4H), 3.14 – 3.00 (m, 4H) ppm.

^{13}C NMR (151 MHz, CD_2Cl_2): δ = 167.1, 166.9, 159.9, 134.8, 132.01, 131.96, 131.5, 125.4, 117.7, 113.9, 40.5, 32.0 ppm.

MS (EI): *m/z* (%) = 426.13 (42) $[M]^{\cdot+}$, 398.12 (40), 380.11 (50), 354.13 (42), 315.09 (100), 287.09 (60), 243.10 (24), 204.06 (39), 176.06 (85), 130.99 (19), 118.99 (23).

HRMS (EI): $^{12}C_{24}{}^{1}H_{18}{}^{14}N_4{}^{16}O_4$ calc.: 426.13280, found: 426.13265.

IR (ATR): $\tilde{\nu}$ = 3466 (w), 3031 (w), 2923 (w), 2252 (w), 2195 (w), 2164 (w), 2050 (w), 1983 (w), 1771 (m), 1704 (s), 1645 (m), 1621 (m), 1428 (m), 1386 (s), 1337 (m), 1318 (m), 1174 (w), 1118 (m), 1082 (m), 1033 (w), 994 (m), 963 (w), 927 (m), 899 (m), 844 (w), 773 (m), 749 (s), 646 (m), 609 (m), 577 (m), 546 (w), 490 (w), 460 (m), 445 (w), 437 (m), 421 (w) cm^{-1}.

m.p.: 205 °C.

***N,N'*-2,3,8,9-Bismethylacetatesuccinimide-11,12-dihydrodibenzo[*c,g*][1,2]-diazocine (19)**

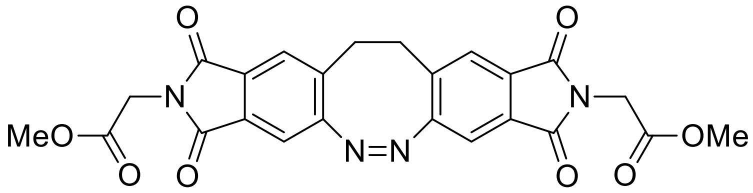

19

Methyl bromoacetate (88.0 mg, 578 µmol).

Yield: 24.7 mg (50.4 µmol, 35 %).

^{1}H NMR (600 MHz, CD_2Cl_2): *δ* = 7.53 (s, 2H), 7.33 (s, 2H), 4.34 (s, 4H), 3.71 (s, 6H), 3.16 – 3.03 (m, 4H) ppm.

^{13}C NMR (151 MHz, CD_2Cl_2): *δ* = 168.0, 166.7, 166.4, 160.0, 135.0, 131.9, 131.4, 125.8, 114.3, 53.0, 39.2, 32.0 ppm.

MS (EI): *m/z* (%) = 490.11 (3) $[M]^{\cdot+}$, 431.10 (9), 403.09 (100), 373.09 (4), 347.08 (9), 319.08 (4), 288.07 (3), 260.07 (19), 204.07 (6), 176.06 (13), 116.03 (1).

HRMS (EI): $^{12}C_{24}{}^{1}H_{18}{}^{14}N_4{}^{16}O_8$ calc.: 490.11246, found: 490.11222.

IR (ATR): $\tilde{\nu}$ = 3037 (w), 2959 (w), 2325 (w), 2194 (w), 2163 (w), 1980 (w), 1744 (m), 1710 (s), 1619 (m), 1394 (s), 1315 (m), 1266 (m), 1209 (m), 1181 (m), 1119 (m), 1073 (m), 1024 (m), 964 (m), 921 (m), 855 (w), 778 (m), 743 (m), 610 (m), 577 (m), 515 (w), 471 (m), 420 (w) cm^{-1}.

S 3 NMR spectra

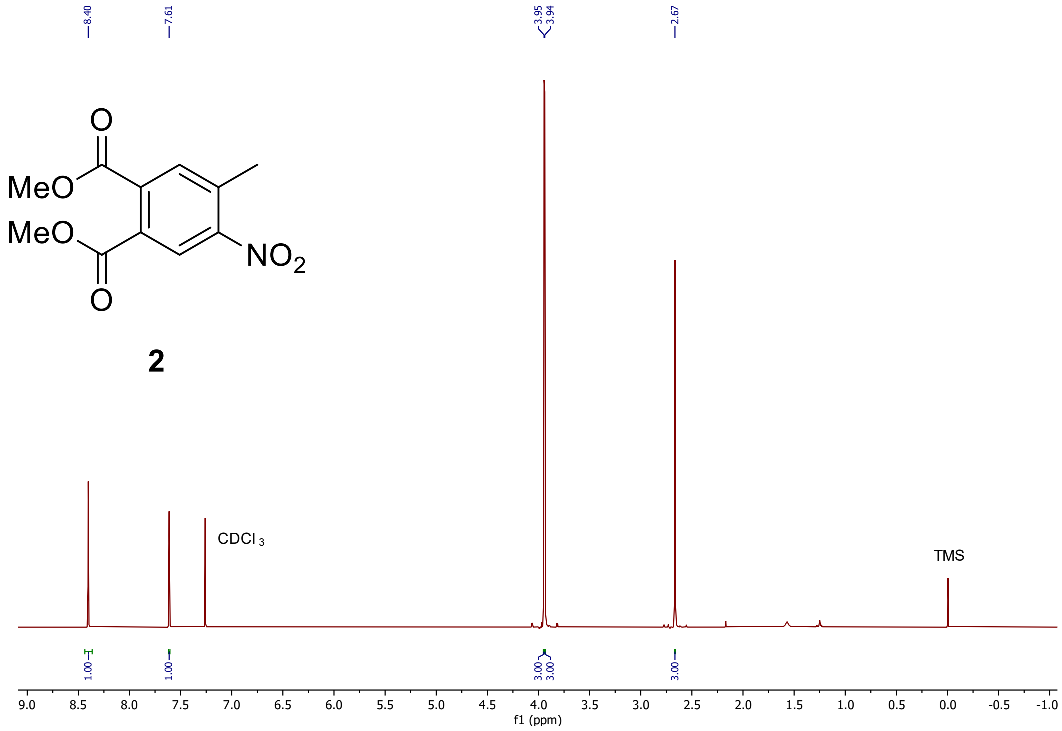

Figure S1. ^{1}H NMR spectrum (600 MHz, $CDCl_3$, 298 K) of compound **2**.

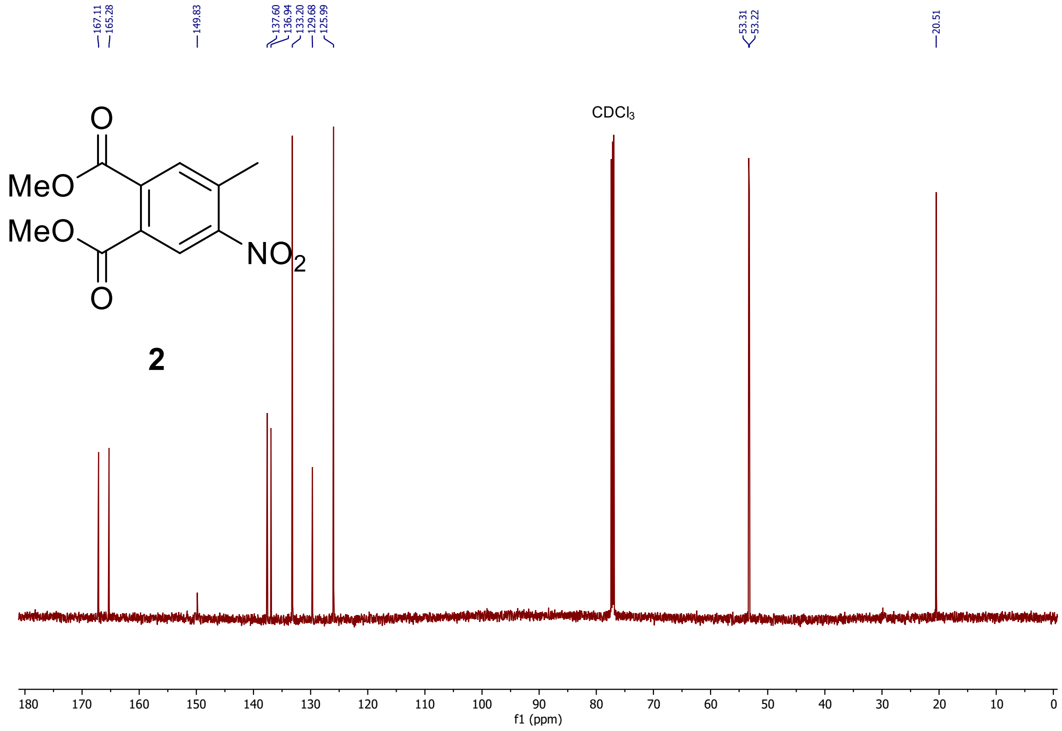

Figure S2. ^{13}C NMR spectrum (151 MHz, $CDCl_3$, 298 K) of compound **2**.

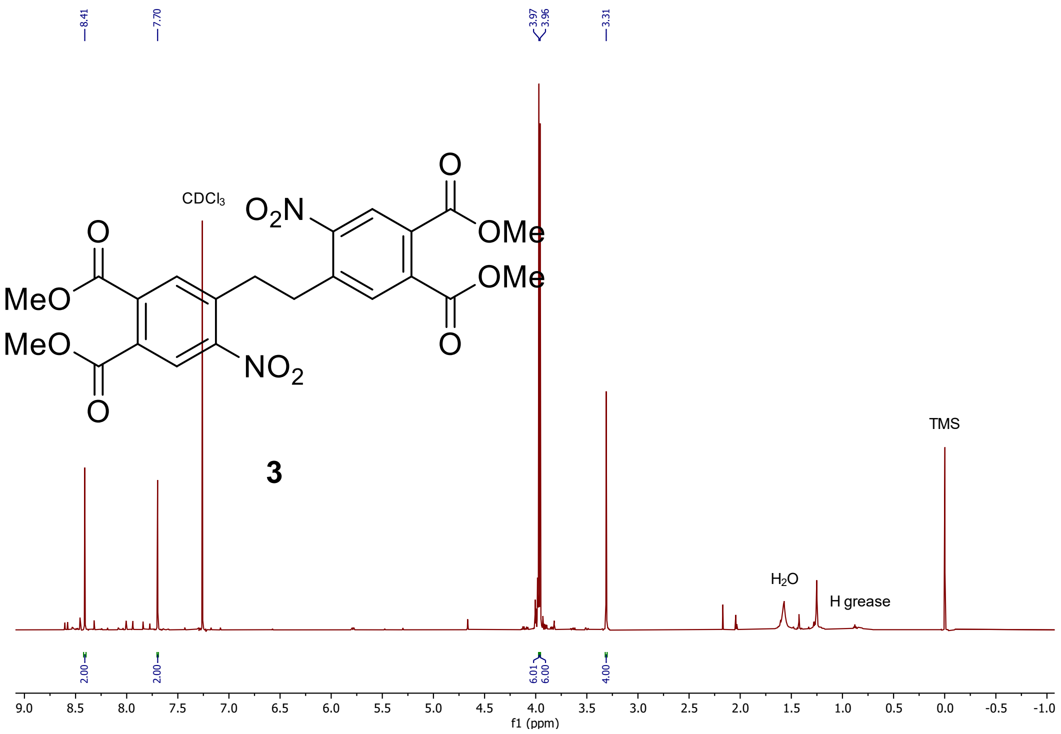

Figure S3. ^{1}H NMR spectrum (600 MHz, $CDCl_3$, 298 K) of compound **3**.

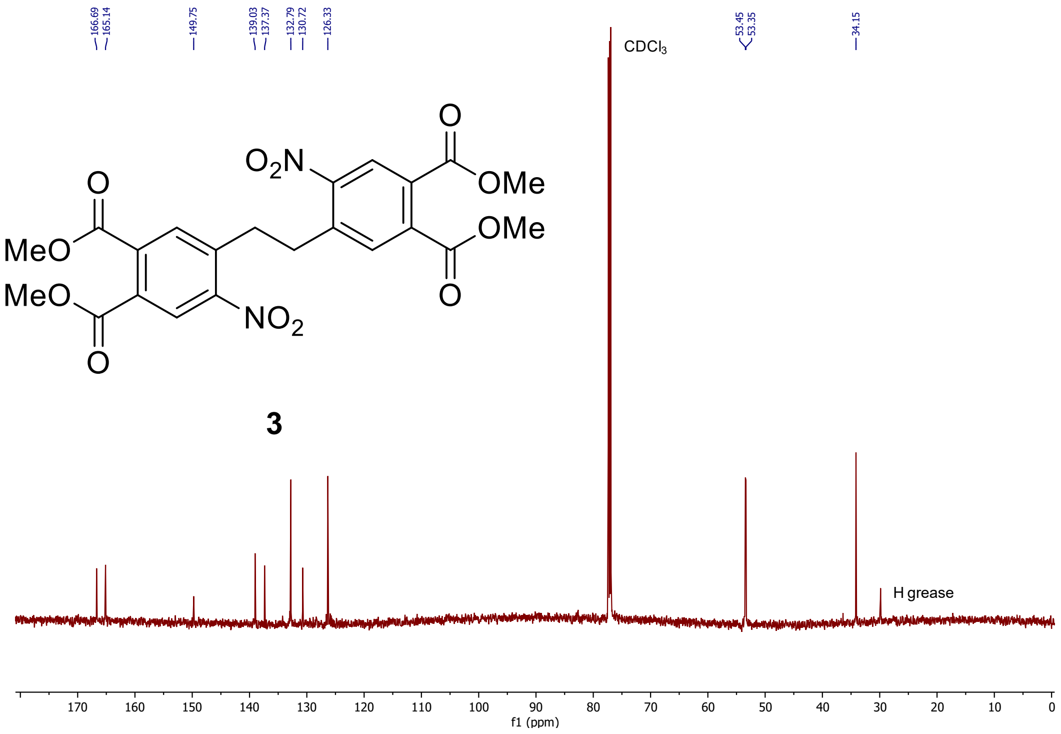

Figure S4. ^{13}C NMR spectrum (151 MHz, $CDCl_3$, 298 K) of compound **3**.

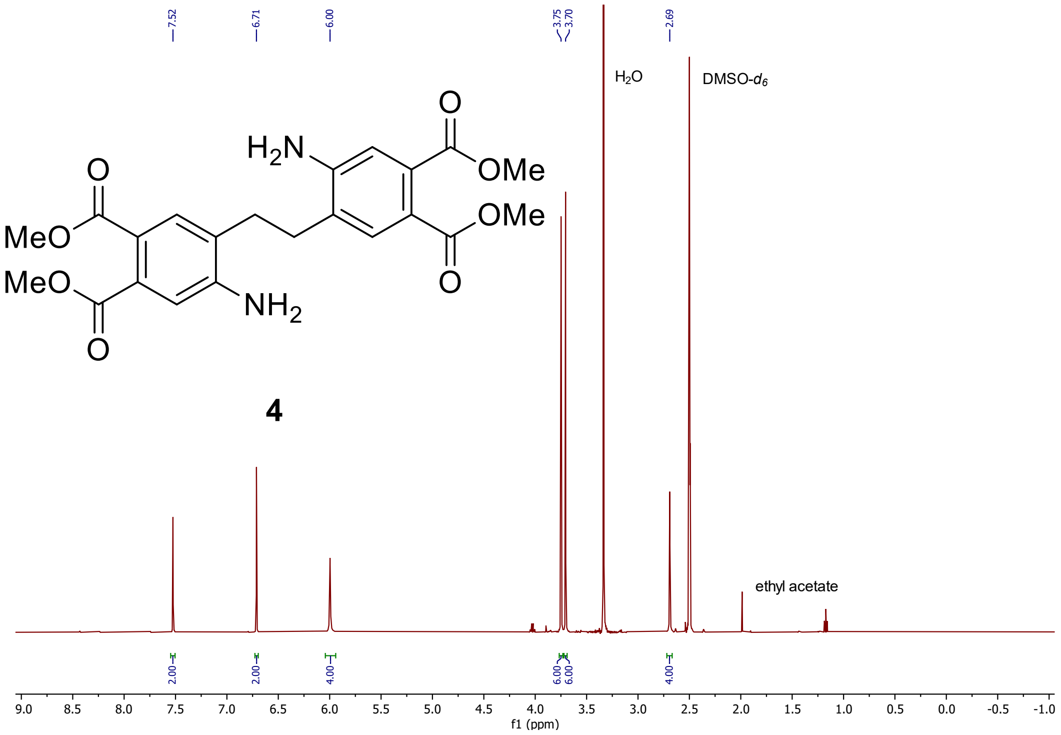

Figure S5. ^{1}H NMR spectrum (500 MHz, DMSO-d_6, 298 K) of compound **4**. Additional ethyl acetate could not be removed after several hours of drying in vacuo.

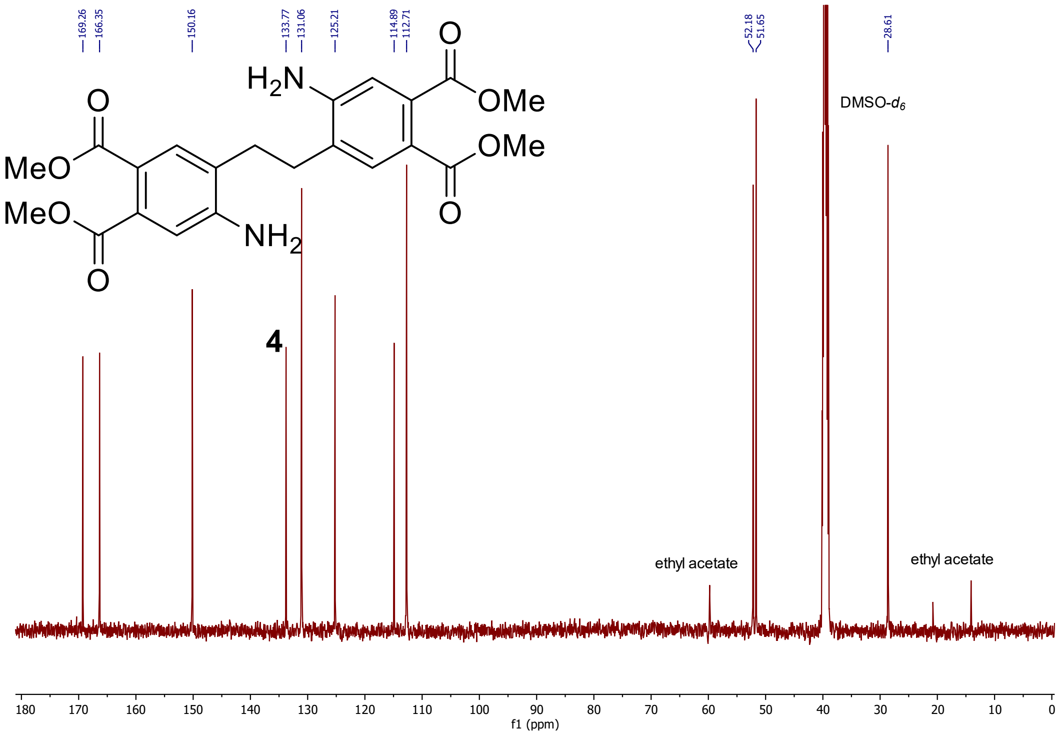

Figure S6. ^{13}C NMR spectrum (126 MHz, DMSO-d_6, 298 K) of compound **4**. Additional ethyl acetate could not be removed after several hours of drying in vacuo.

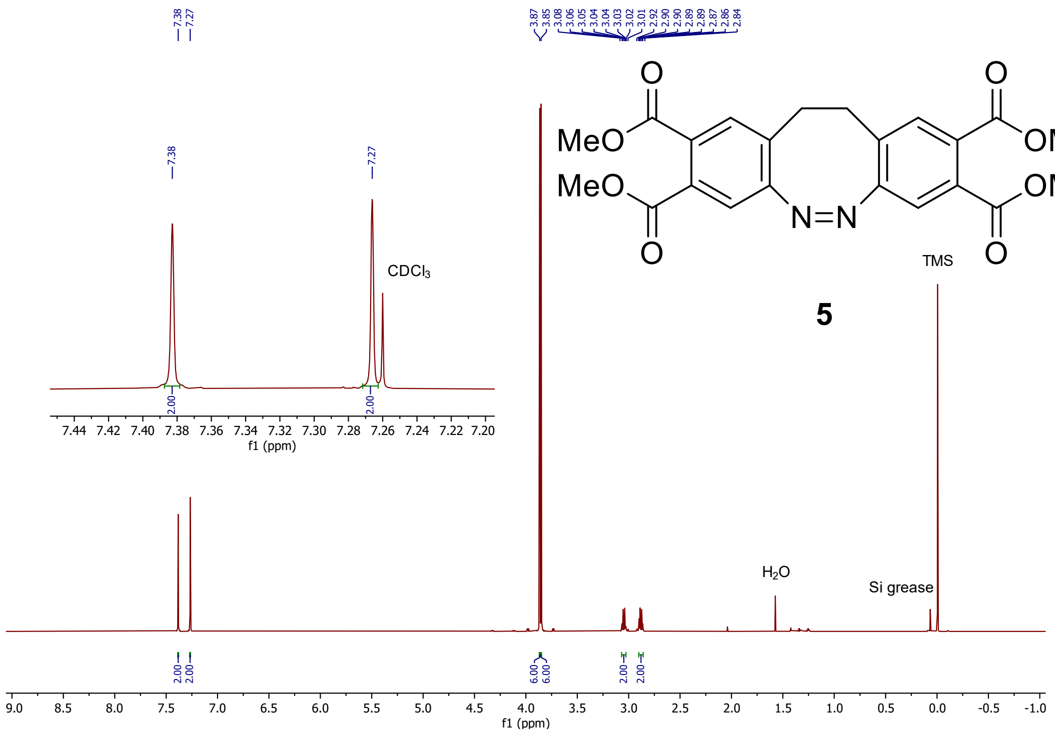

Figure S7. ^{1}H NMR spectrum (600 MHz, $CDCl_3$, 298 K) of compound **5**.

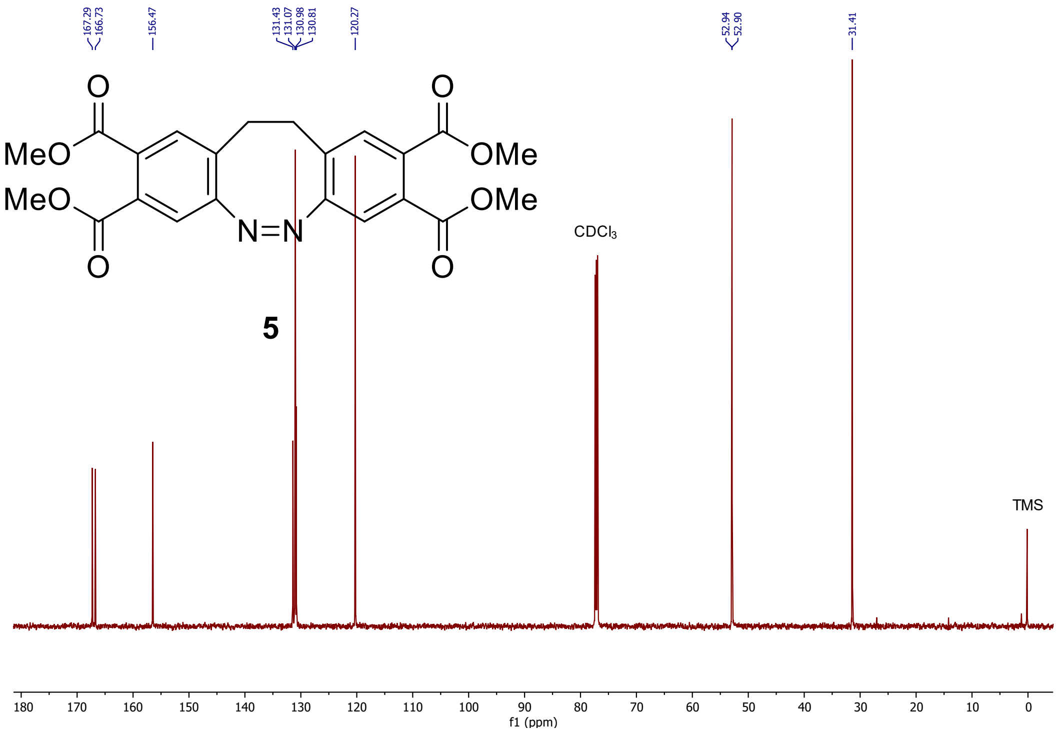

Figure S8. ^{13}C NMR spectrum (151 MHz, $CDCl_3$, 298 K) of compound **5**.

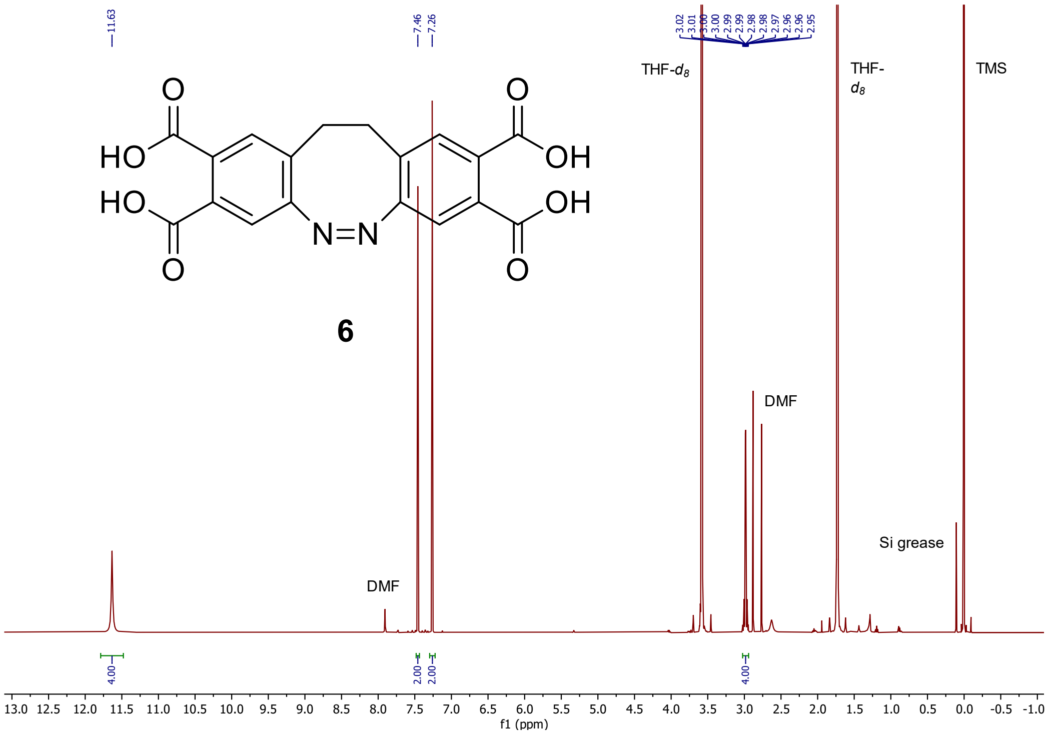

Figure S9. ^{1}H NMR spectrum (600 MHz, THF-d_8, 298 K) of compound **6**. Additional DMF could not be removed after codestillation with *n*-heptane.

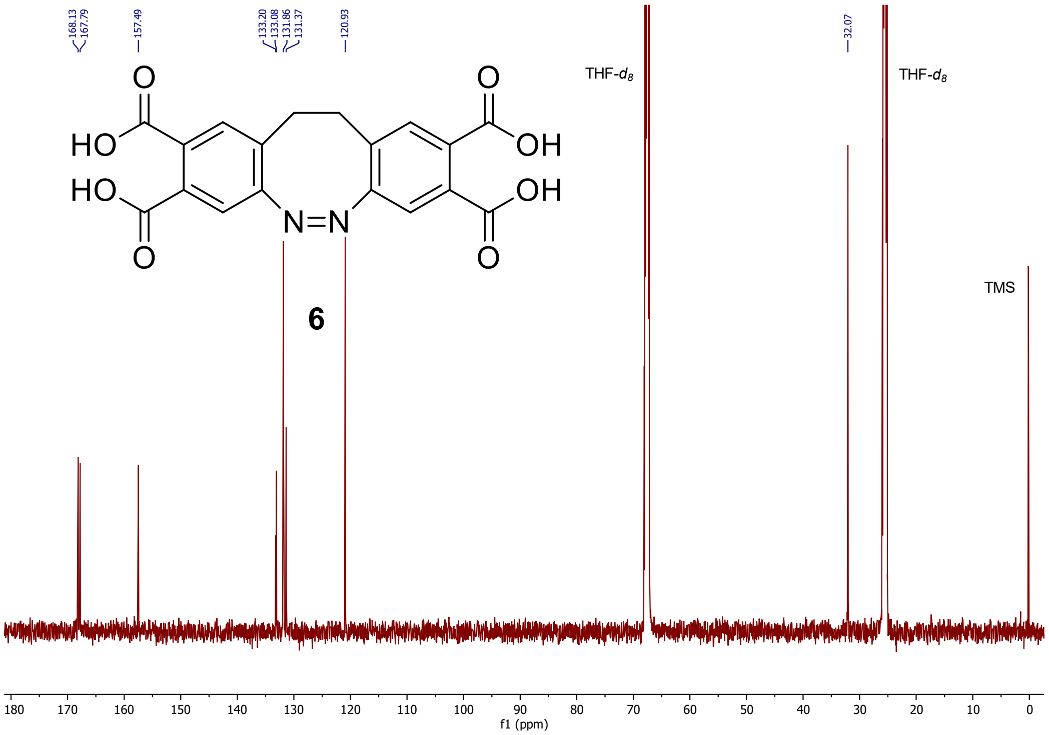

Figure S10. ^{13}C NMR spectrum (151 MHz, THF-d_8, 298 K) of compound **6**.

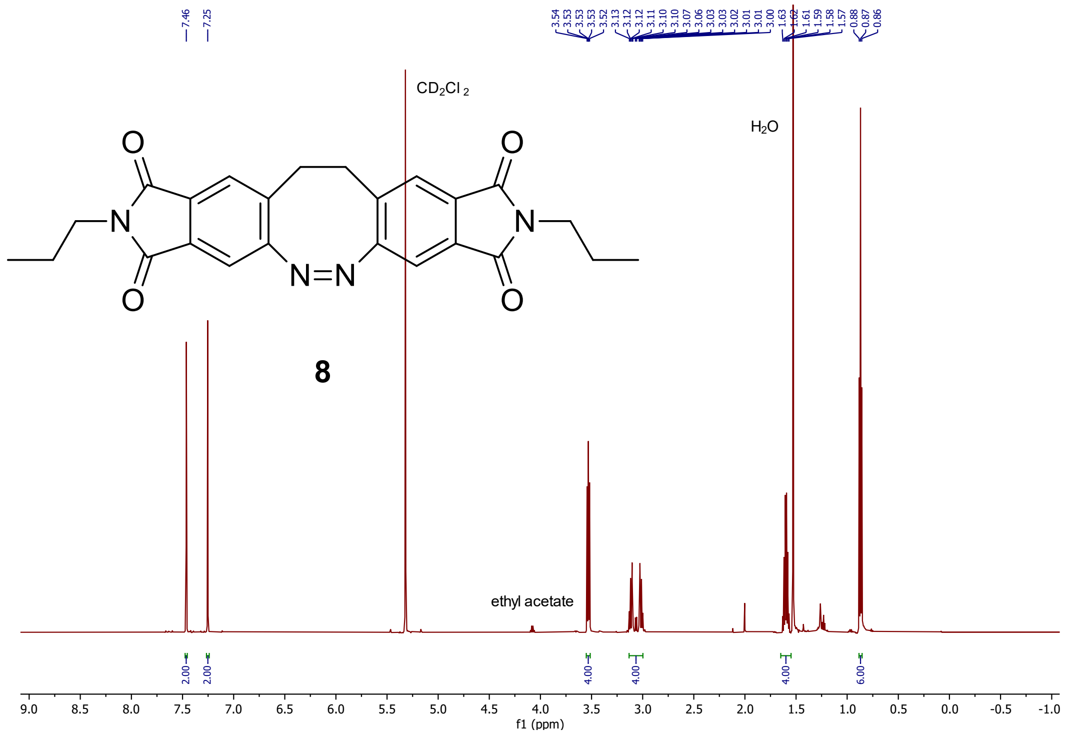

Figure S11. ^{1}H NMR spectrum (600 MHz, CD_2Cl_2, 298 K) of compound **8**. Additional ethyl acetate could not be removed after several hours of drying in vacuo.

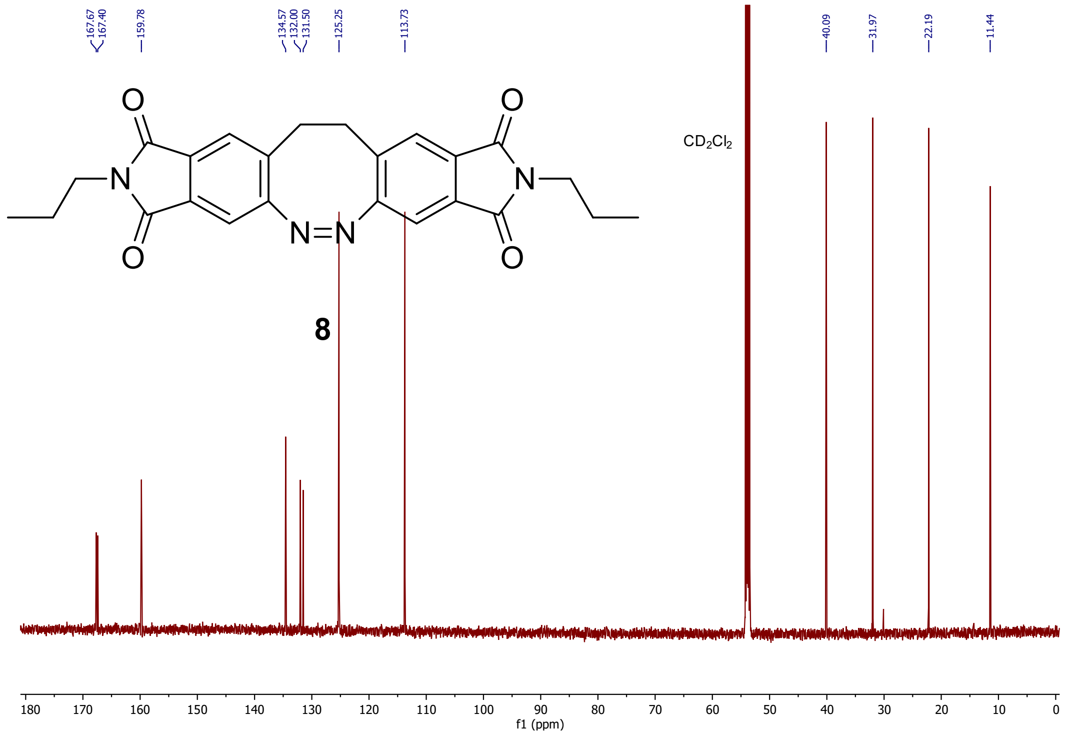

Figure S12. ^{13}C NMR spectrum (151 MHz, CD_2Cl_2, 298 K) of compound **8**.

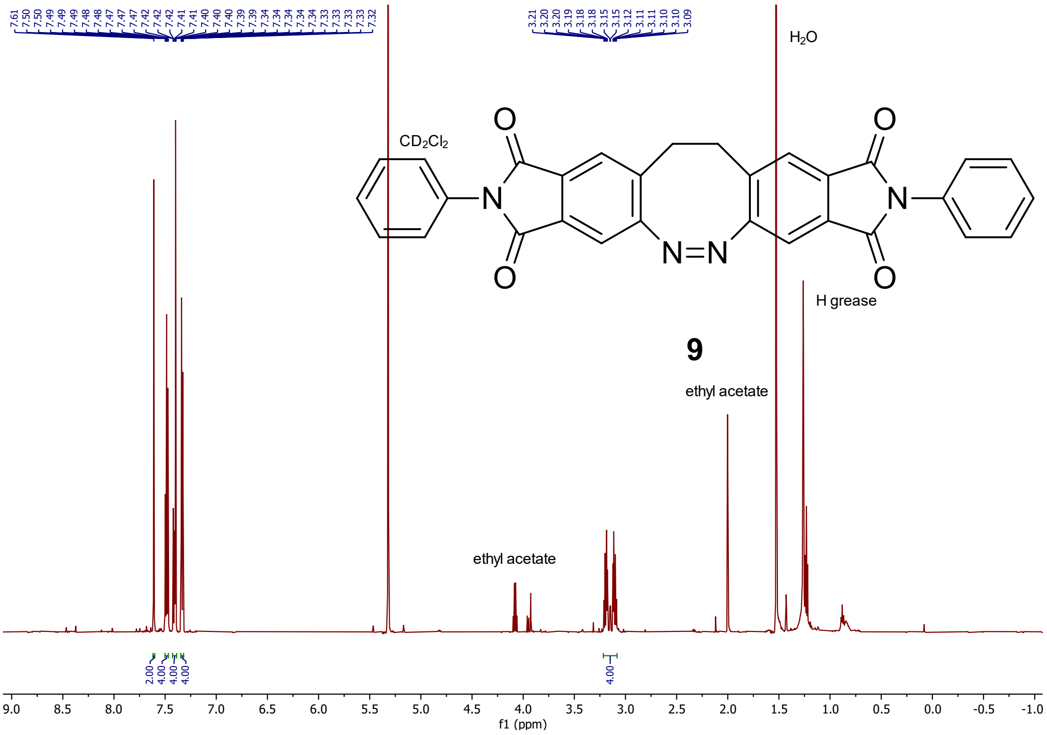

Figure S13. ^{1}H NMR spectrum (600 MHz, CD_2Cl_2, 298 K) of compound **9**. Additional ethyl acetate could not be removed after several hours of drying in vacuo.

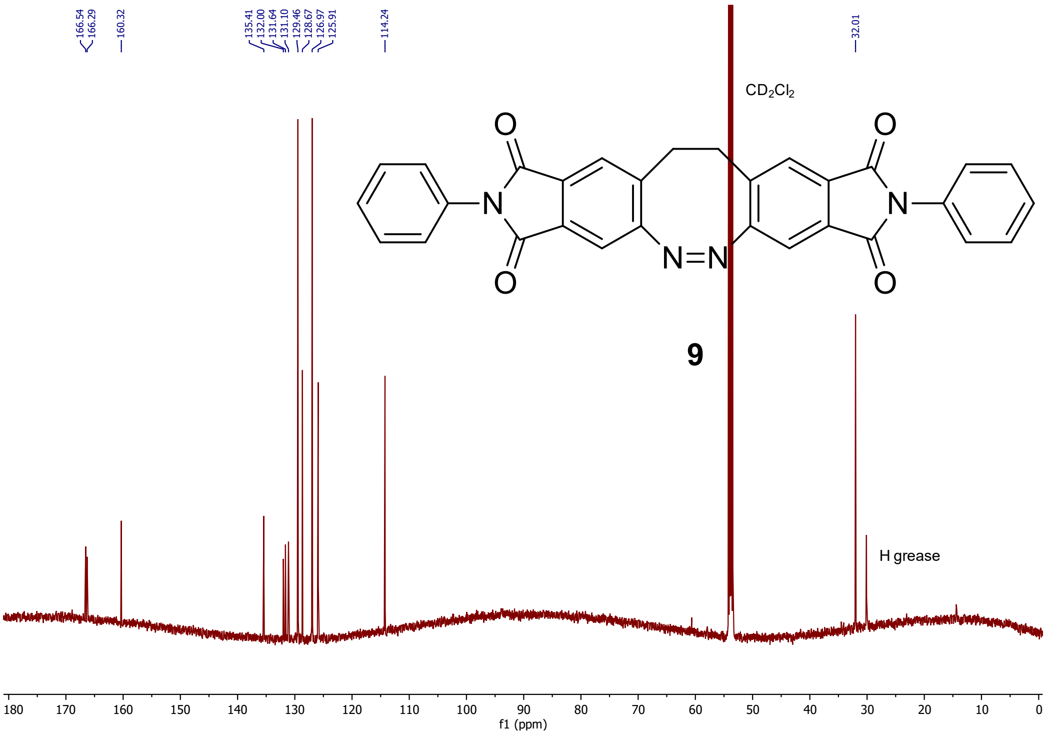

Figure S14. ^{13}C NMR spectrum (151 MHz, CD_2Cl_2, 298 K) of compound **9**.

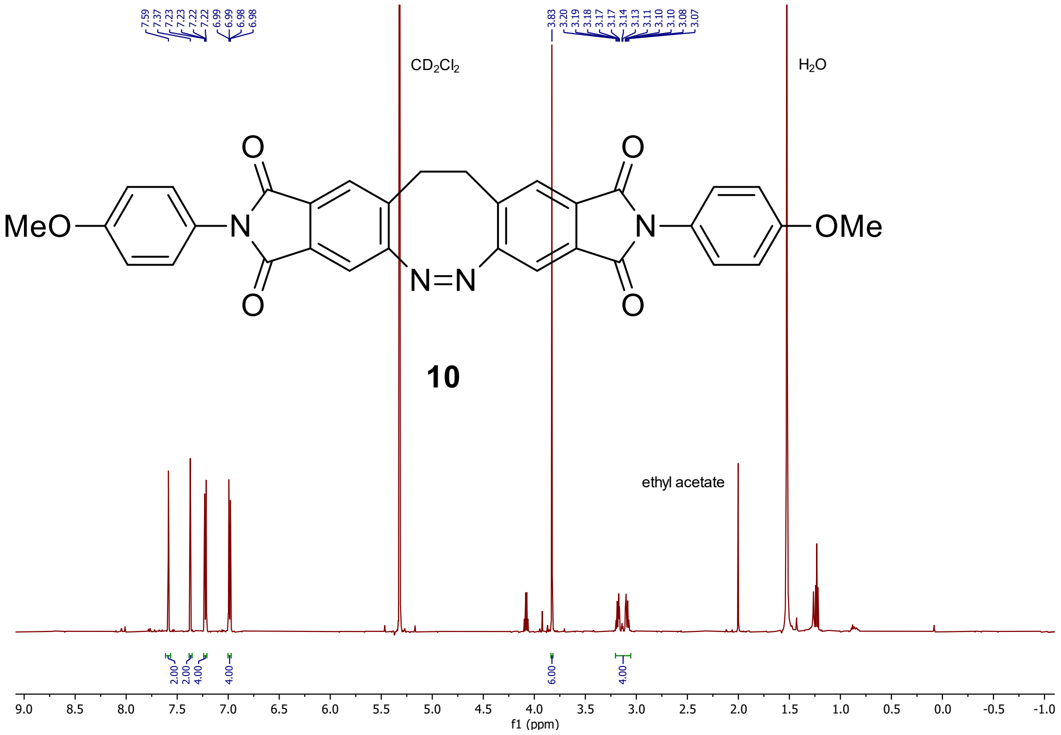

Figure S15. ^{1}H NMR spectrum (600 MHz, CD_2Cl_2, 298 K) of compound **10**. Additional ethyl acetate could not be removed after several hours of drying in vacuo.

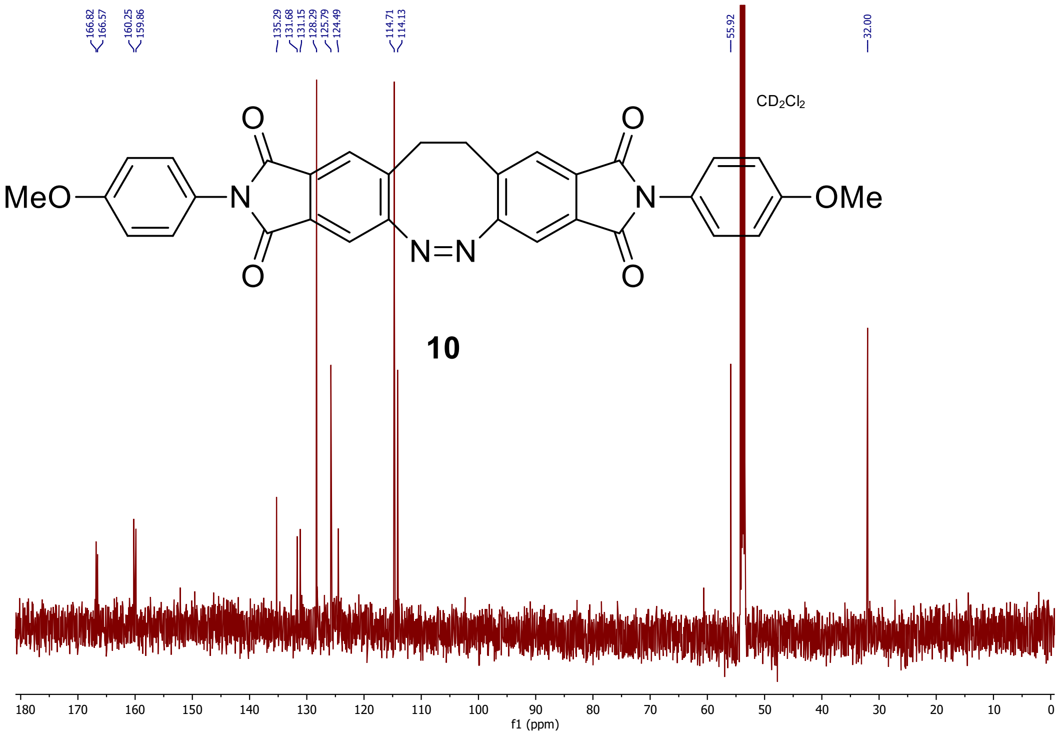

Figure S16. ^{13}C NMR spectrum (151 MHz, CD_2Cl_2, 298 K) of compound **10**.

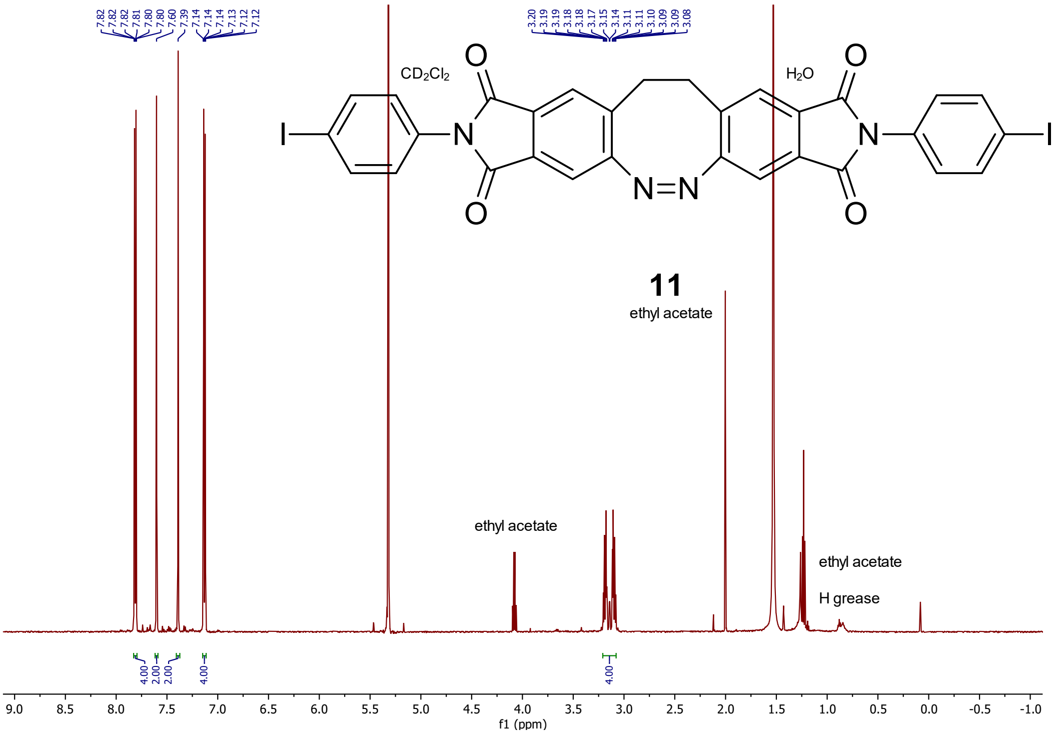

Figure S17. ^{1}H NMR spectrum (600 MHz, CD_2Cl_2, 298 K) of compound **11**. Additional ethyl acetate could not be removed after several hours of drying in vacuo.

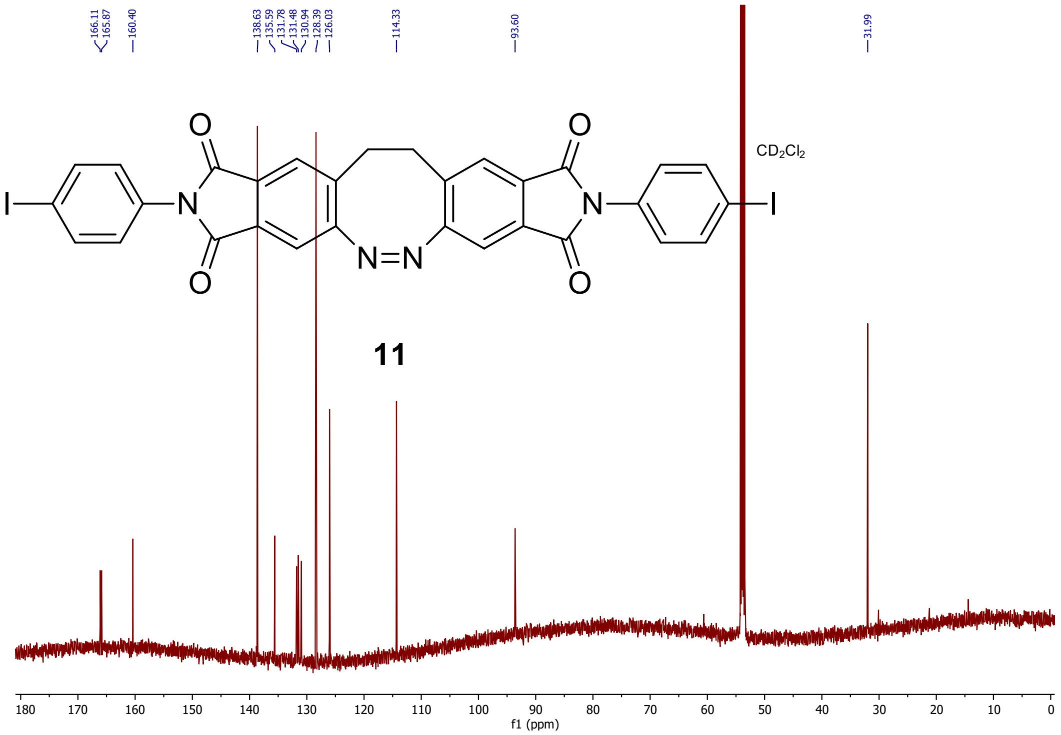

Figure S18. ^{13}C NMR spectrum (151 MHz, CD_2Cl_2, 298 K) of compound **11**.

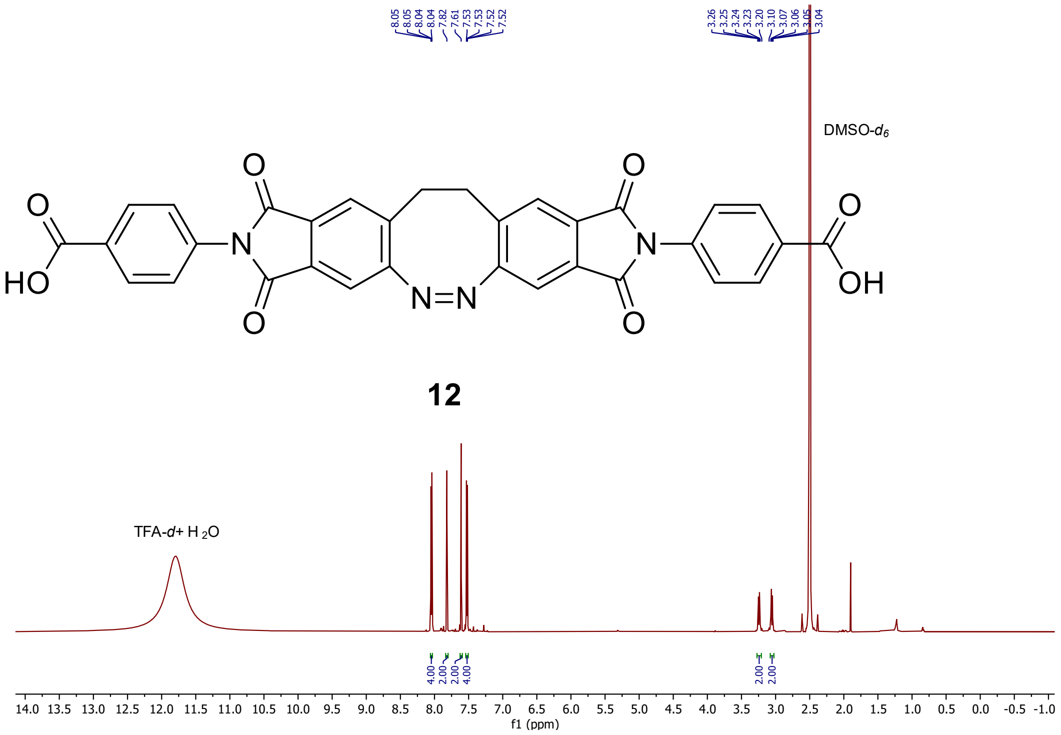

Figure S19. ^{1}H NMR spectrum (600 MHz, DMSO-d_6 + TFA-d, 298 K) of compound **12**.

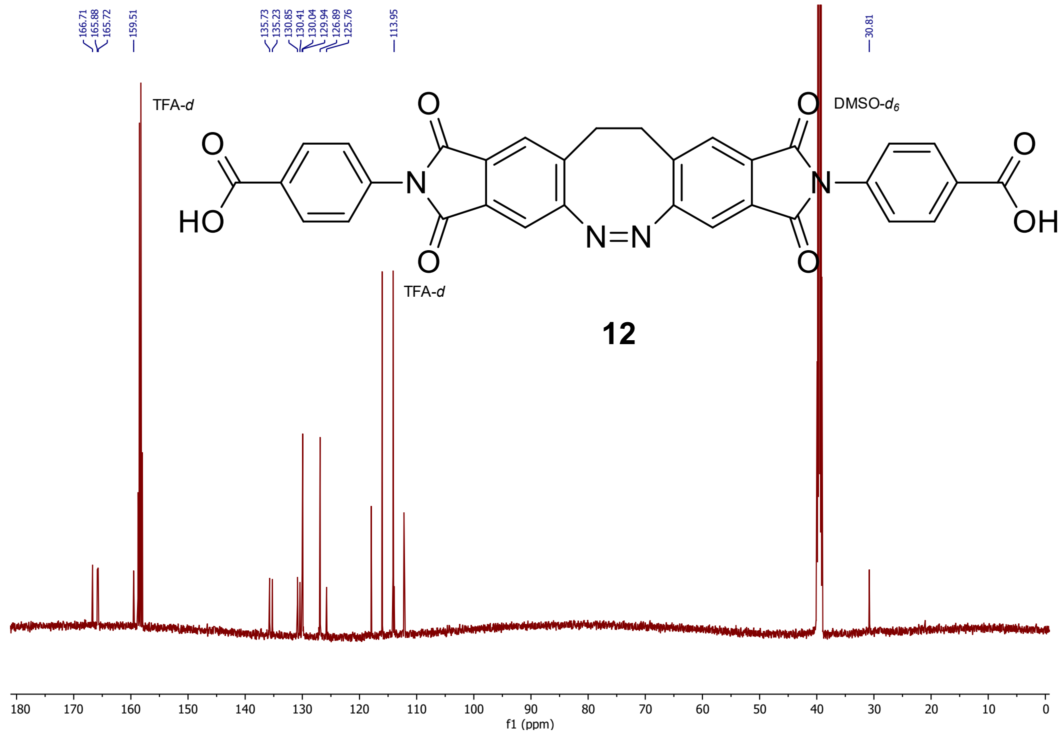

Figure S20. ^{13}C NMR spectrum (151 MHz, DMSO-d_6 + TFA-d, 298 K) of compound **12**.

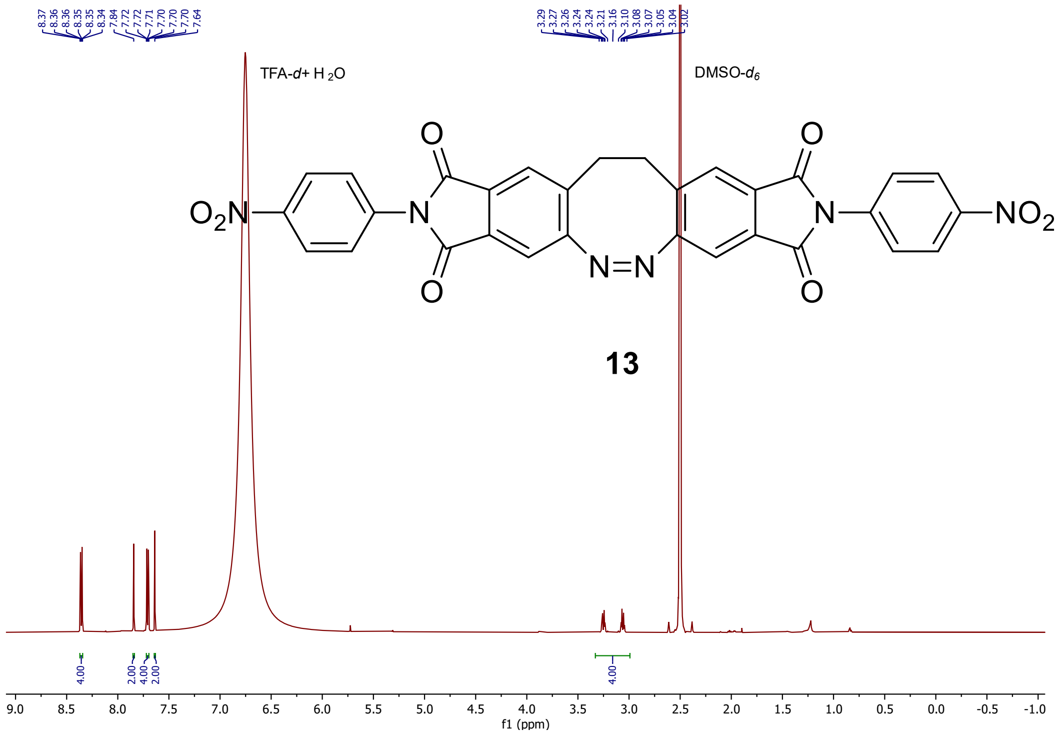

Figure S21. ^{1}H NMR spectrum (600 MHz, DMSO-*d6* + TFA-*d*, 298 K) of compound **13**.

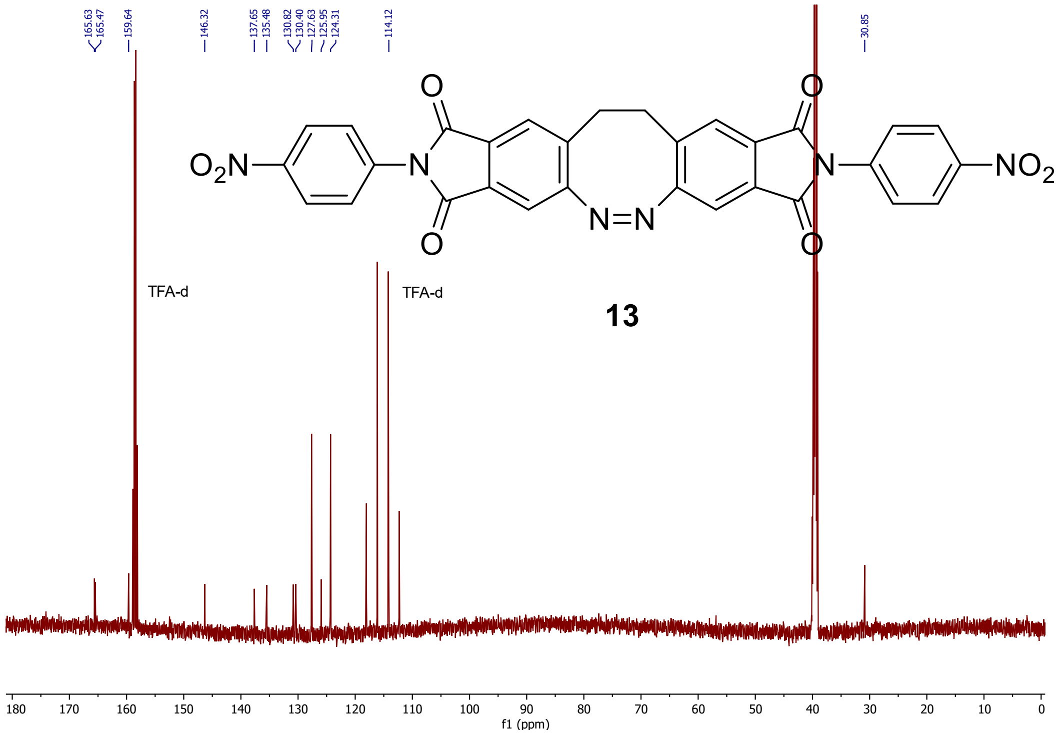

Figure S22. ^{13}C NMR spectrum (151 MHz, DMSO-*d6* + TFA-*d*, 298 K) of compound **13**.

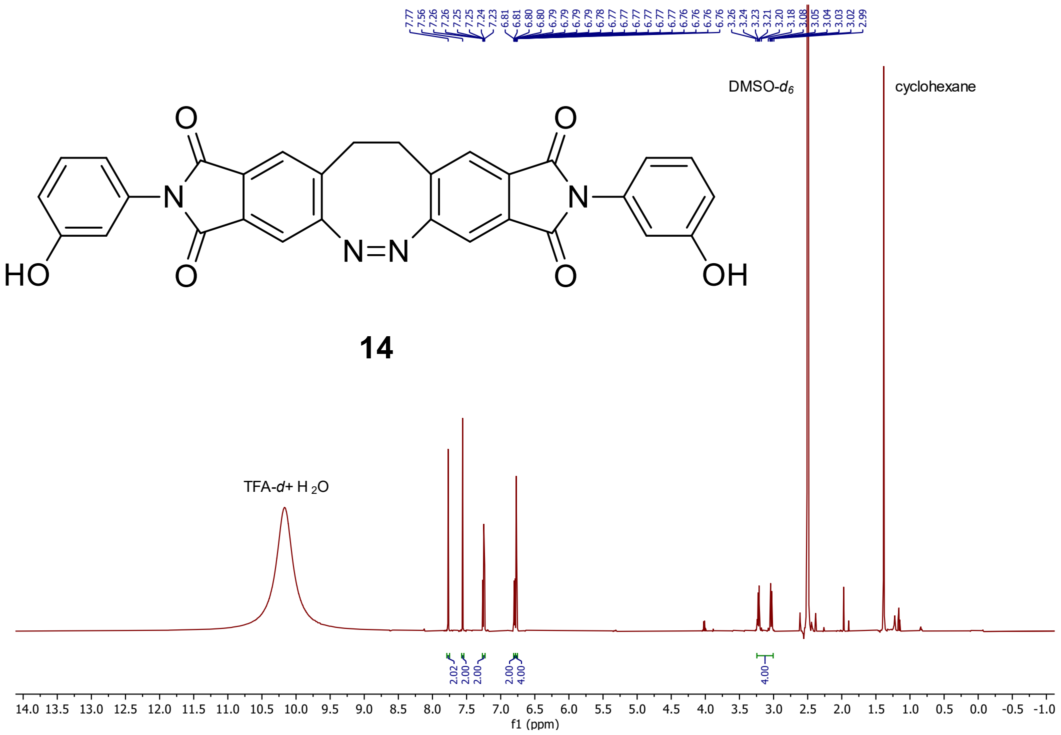

Figure S23. ^{1}H NMR spectrum (600 MHz, DMSO-d_6 + TFA-d, 298 K) of compound **14**. Additional cyclohexane could not be removed after several hours of drying in vacuo.

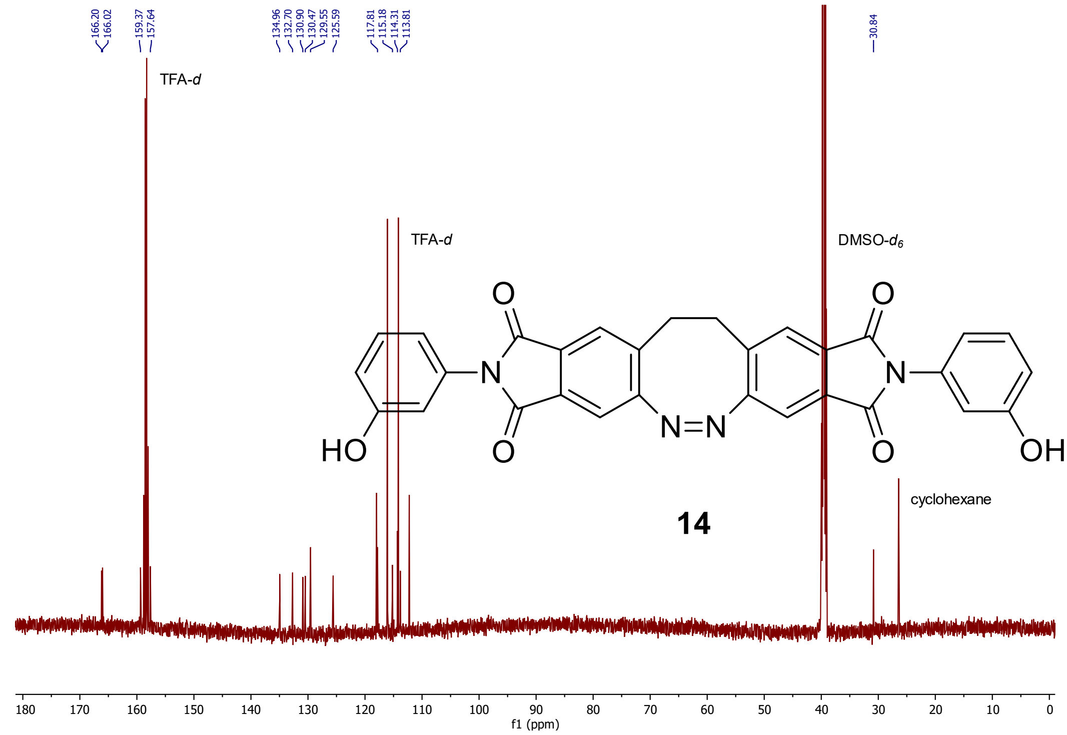

Figure S24. ^{13}C NMR spectrum (151 MHz, DMSO-d_6 + TFA-d, 298 K) of compound **14**. Additional cyclohexane could not be removed after several hours of drying in vacuo.

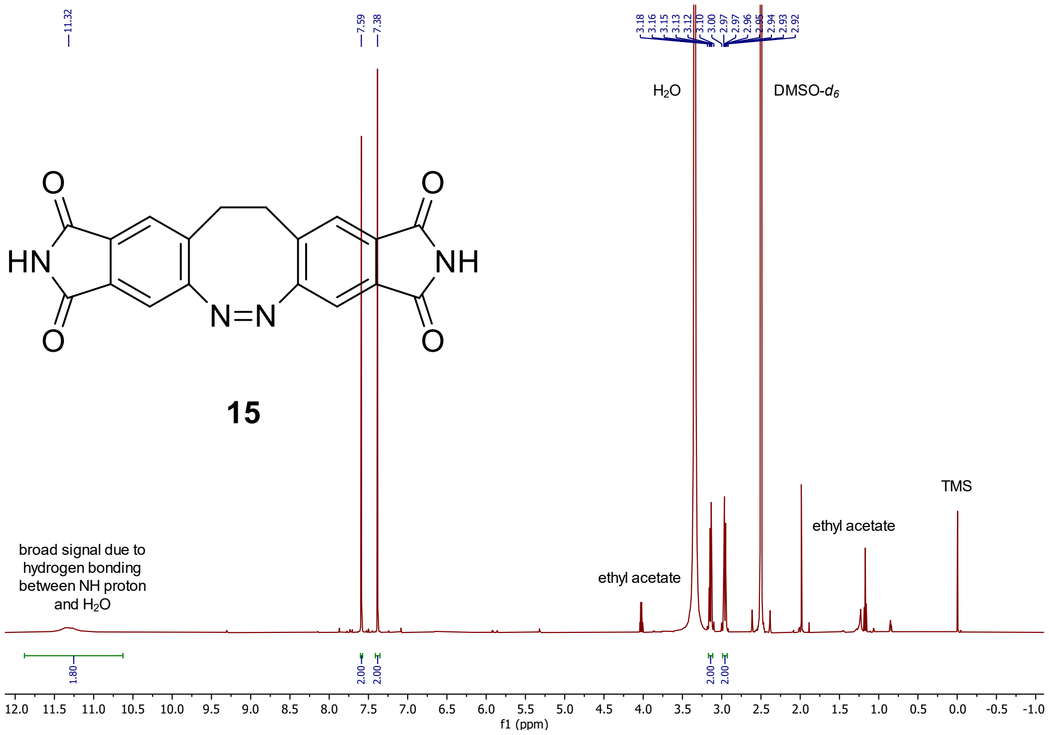

Figure S25. ^{1}H NMR spectrum (600 MHz, DMSO-d_6, 298 K) of compound **15**. Additional ethyl acetate could not be removed after several hours of drying in vacuo.

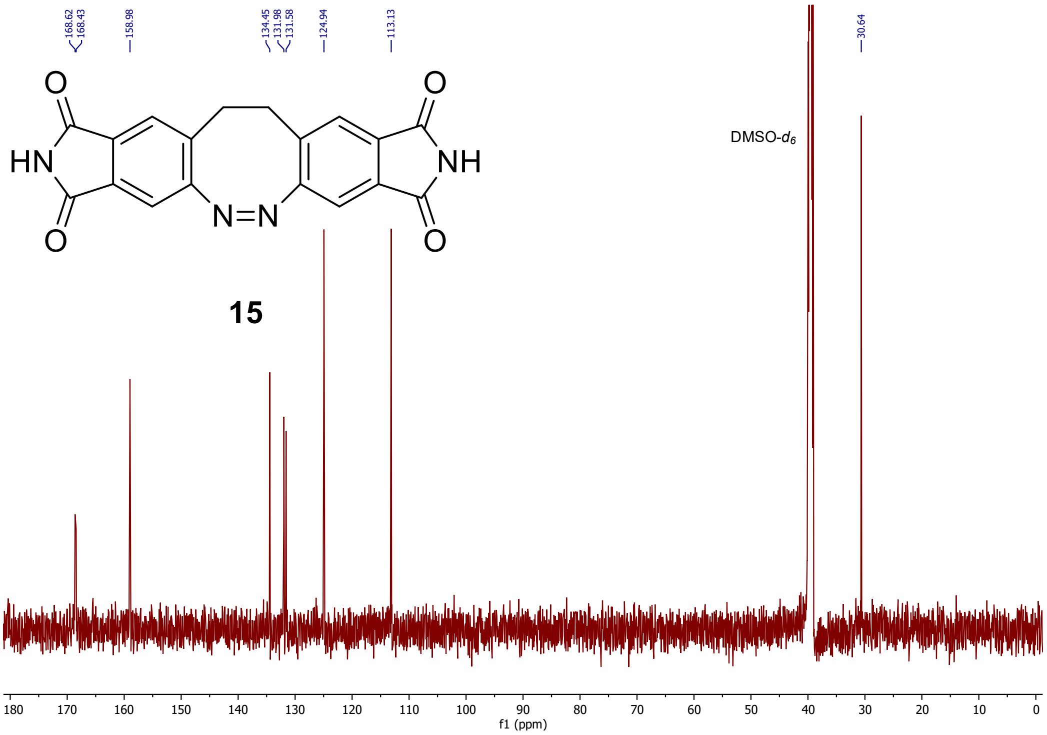

Figure S26. ^{13}C NMR spectrum (151 MHz, DMSO-d_6, 298 K) of compound **15**.

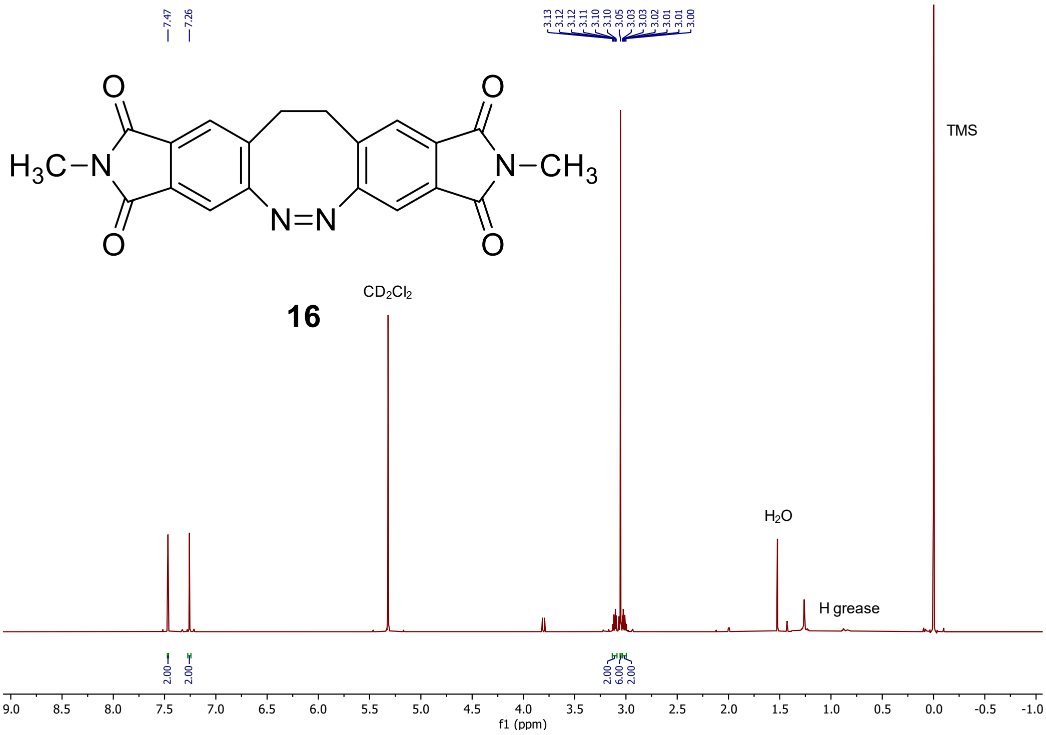

Figure S27. ^{1}H NMR spectrum (600 MHz, CD_2Cl_2, 298 K) of compound **16**.

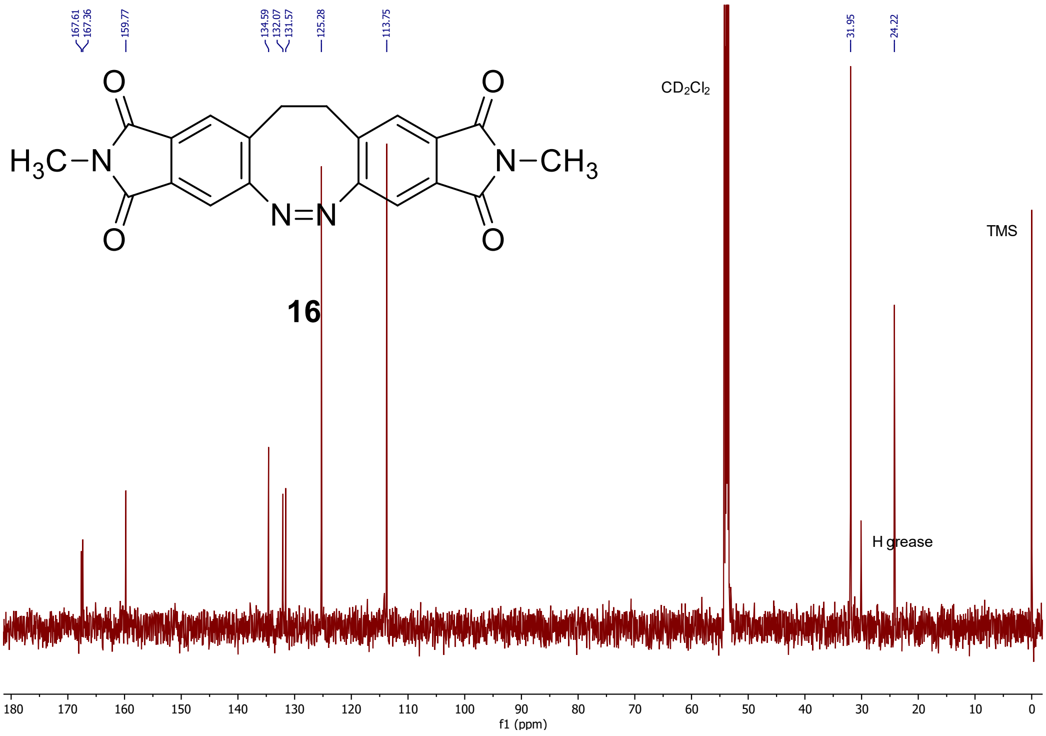

Figure S28. ^{13}C NMR spectrum (151 MHz, CD_2Cl_2, 298 K) of compound **16**.

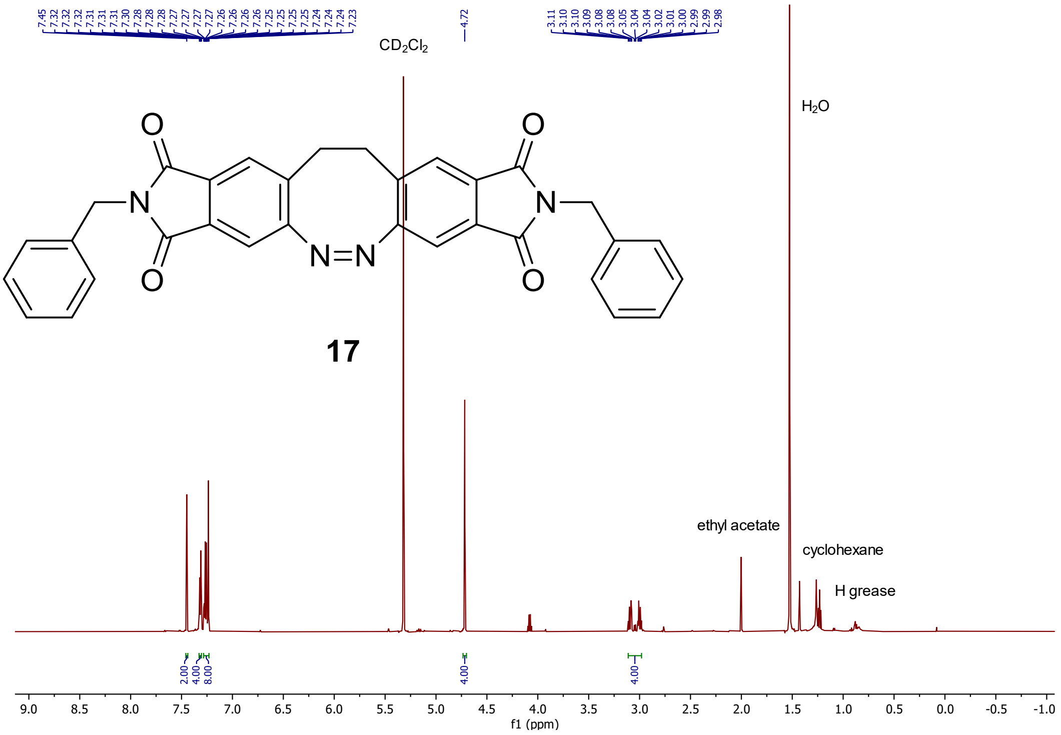

Figure S29. ^{1}H NMR spectrum (600 MHz, CD_2Cl_2, 298 K) of compound **17**. Additional ethyl acetate could not be removed after several hours of drying in vacuo.

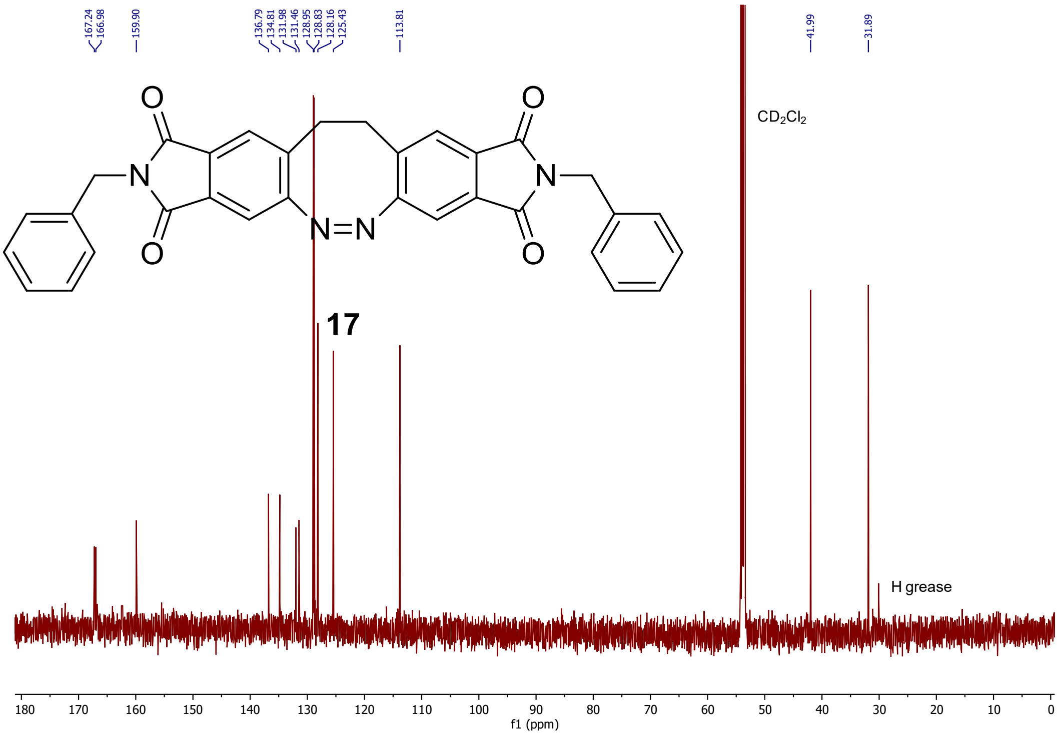

Figure S30. ^{13}C NMR spectrum (151 MHz, CD_2Cl_2, 298 K) of compound **17**.

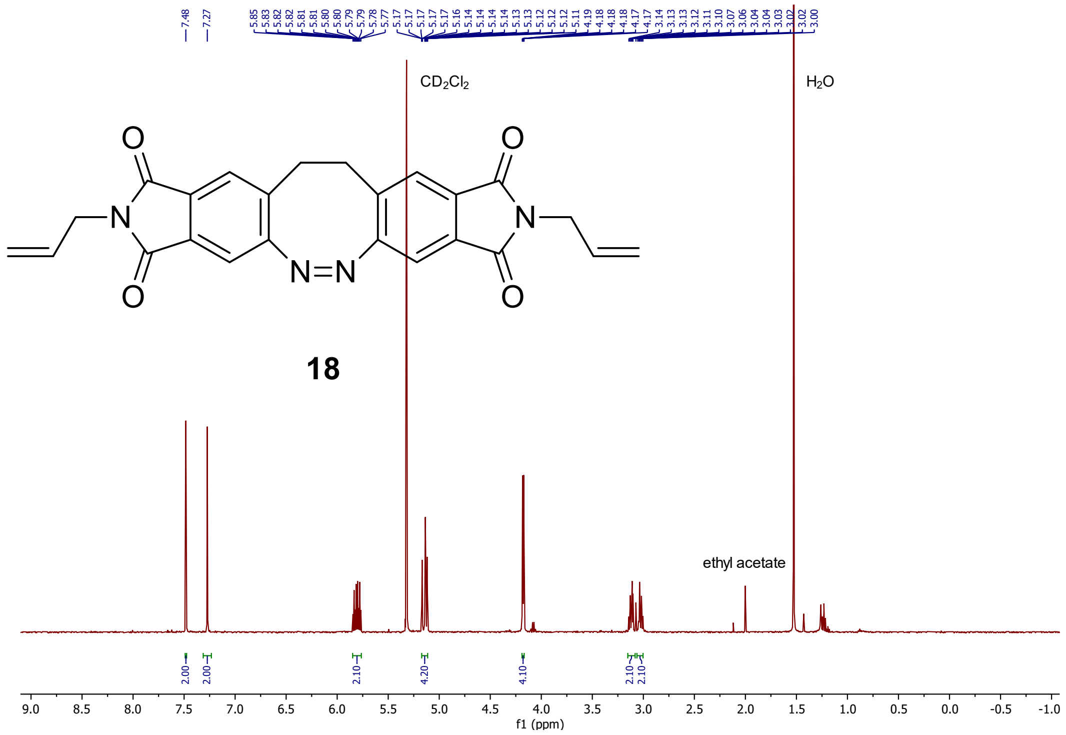

Figure S31. ^{1}H NMR spectrum (600 MHz, CD_2Cl_2, 298 K) of compound **18**. Additional ethyl acetate could not be removed after several hours of drying in vacuo.

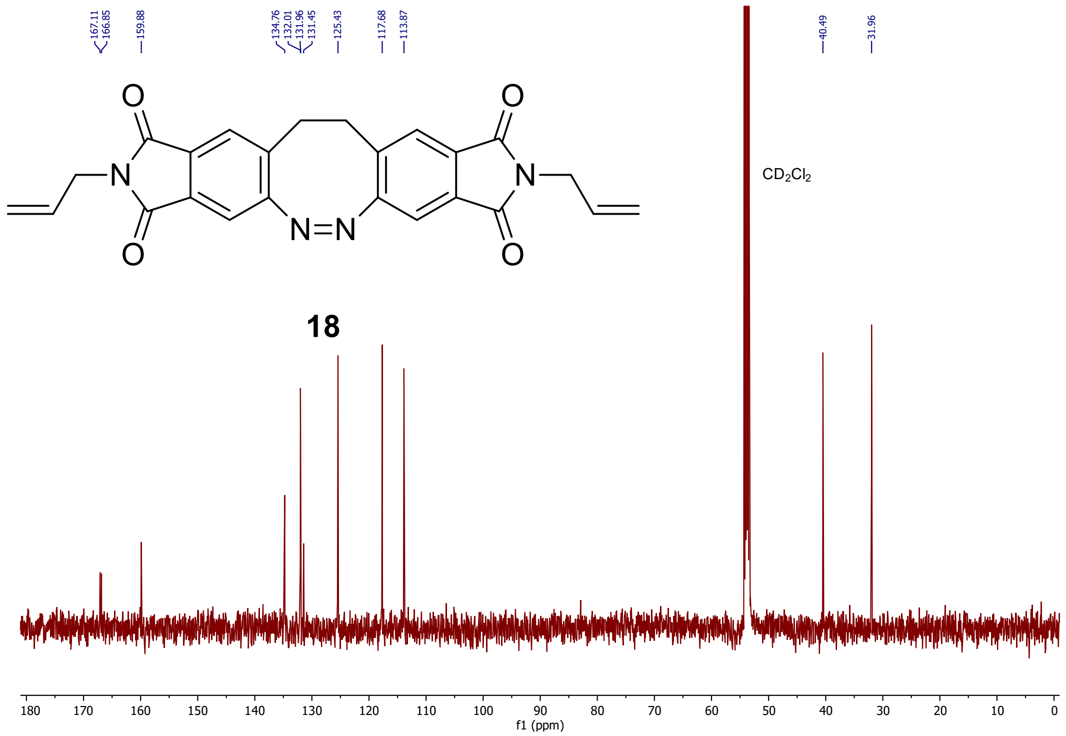

Figure S32. ^{13}C NMR spectrum (151 MHz, CD_2Cl_2, 298 K) of compound **18**.

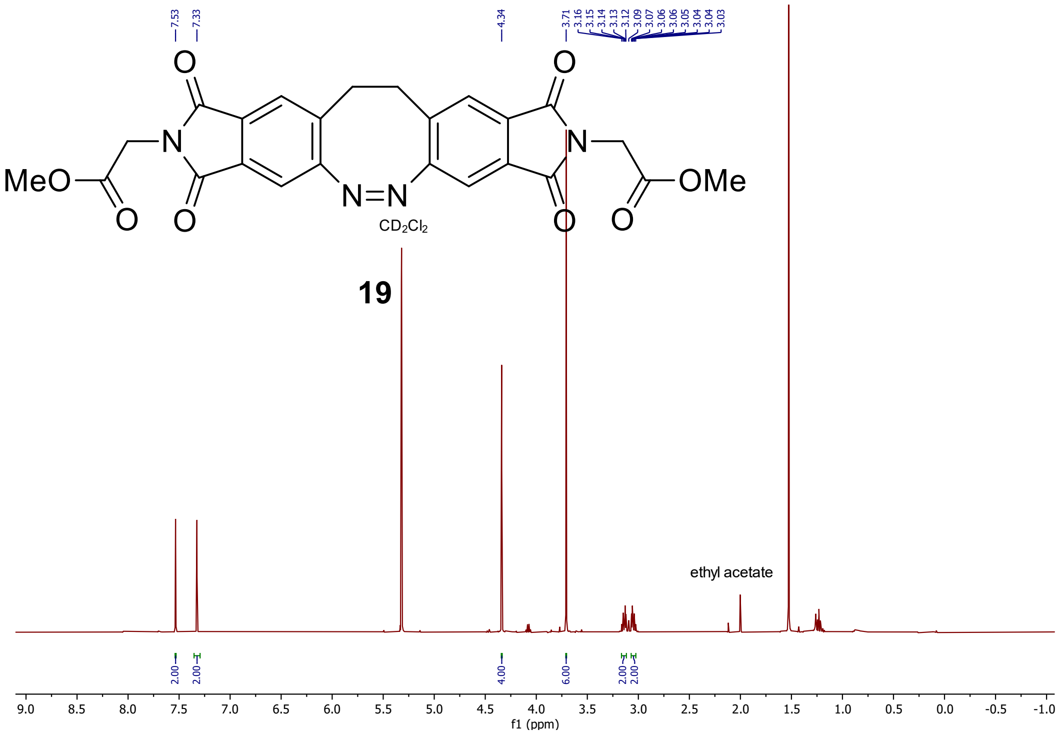

Figure S33. ^{1}H NMR spectrum (600 MHz, CD_2Cl_2, 298 K) of compound **19**. Additional ethyl acetate could not be removed after several hours of drying in vacuo.

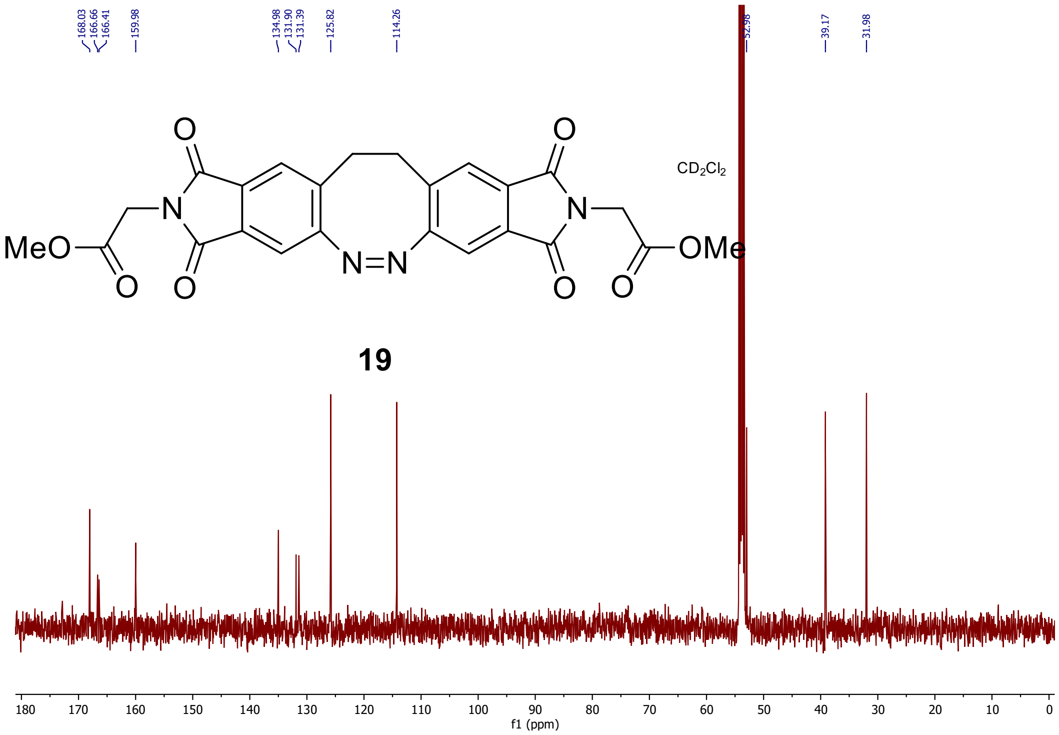

Figure S34. ^{13}C NMR spectrum (151 MHz, CD_2Cl_2, 298 K) of compound **19**.

S 4 Photophysical characterization

S 4.1 UV/vis absorption spectra and thermal half-lives

To determine the optimum wavelengths for excitation to the photostationary state, 300 µM solutions in THF (compounds **5**, **6**, **8** – **11** and **14** – **19**) or DMSO (compounds **12** and **13**) were irradiated with 365 nm, 385 nm, 405 nm and 420 nm (blue light) for 30 s at 298 K respectively. Re-isomerization to the thermodynamically stable *Z* isomer was carried out by irradiation with 530 nm (green light) for 30 s at 298 K. The UV/vis spectra after each irradiation were measured at 298 K within a measuring range of 250 nm – 750 nm. Due to its instability, compound **7** could not be isolated in pure form and therefore was not characterized by UV/vis spectroscopy.

The half-lives $t_{1/2}$ of the thermal re-isomerization of the photoswitches **5**, **6** and **8** – **19** were determined by plotting ln(A_t/A_0) vs. time in assumption of first-order reactions. The slope of the linear fit gives the time constant *k*, which is used for the calculation of the half-life $t_{1/2}$ according to equation (1):

$$t_{1/2} = \frac{\ln(2)}{k} \qquad (1)$$

A total of 30 measurements were recorded every 180 s (120 s for compounds **5** and **6**) at 298 K after excitation to the photostationary state for 30 s at 298 K with the previously determined optimum wavelengths. In order to avoid errors caused by unwanted photoswitching with the measuring beam of the UV spectrometer only a small interval of ± 5 nm at the respective λ_{max} (*E* isomer) of each photoswitch was recorded within each measurement. Consequently, 30 absorption data points at λ_{max} of each compound were used to determine the half-life $t_{1/2}$.

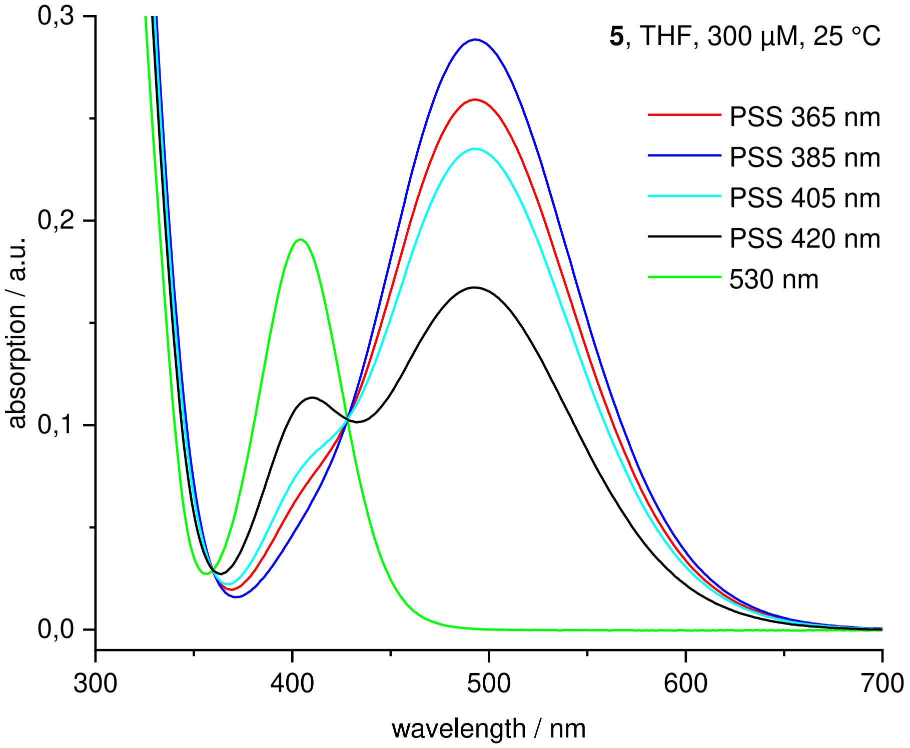

Figure S35: UV/vis spectra (THF, 300 µM, 298 K) of compound **5** after irradiation with 365 – 530 nm for 30 s at 298 K (λ_{max} (*Z*) = 405 nm, λ_{max} (*E*) = 493 nm).

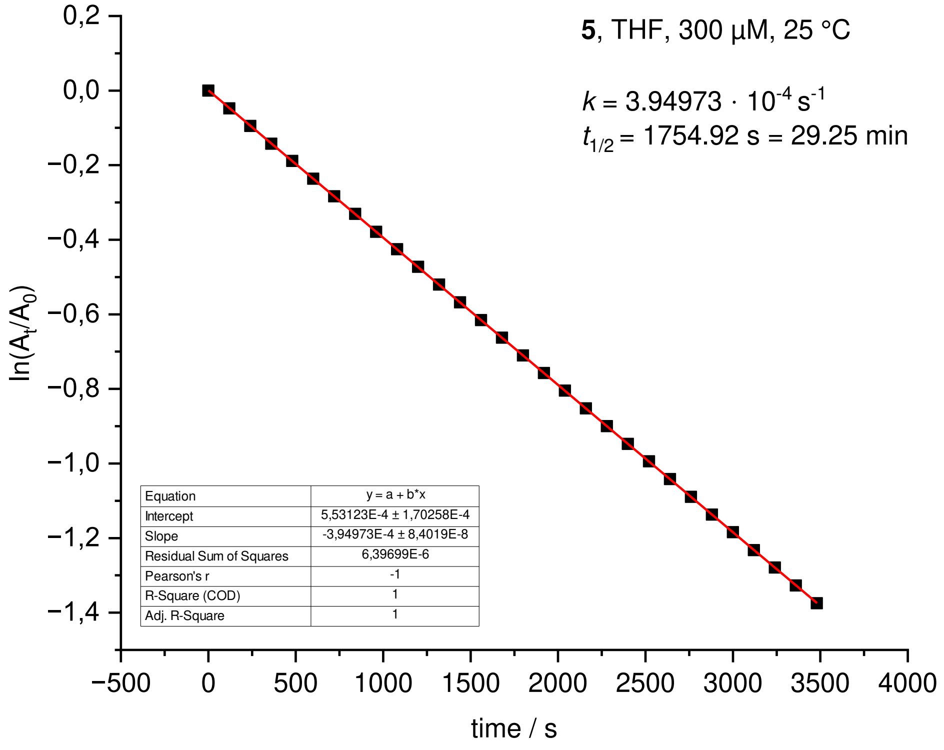

Figure S36: Determination of the thermal half-life $t_{1/2}$ of compound **5** (THF, 300 µM, 298 K) after irradiation with 385 nm (k = 3.94973 · 10^{-4} s^{-1}, $t_{1/2}$ = 29.25 min).

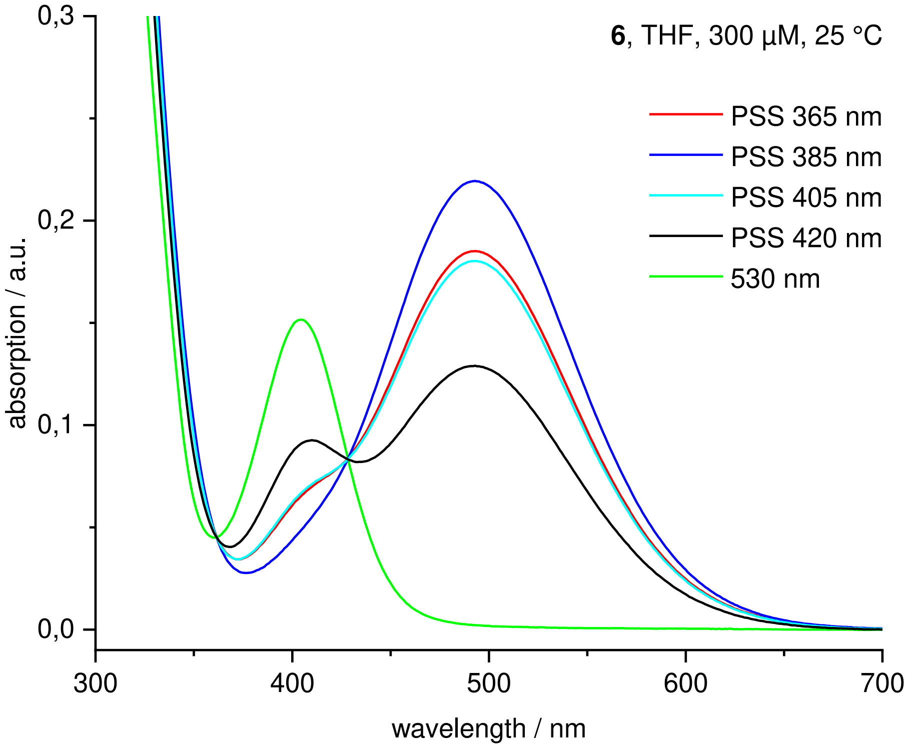

Figure S37: UV/vis spectra (THF, 300 µM, 298 K) of compound **6** after irradiation with 365 – 530 nm for 30 s at 298 K (λ_{max} (Z) = 405 nm, λ_{max} (E) = 493 nm).

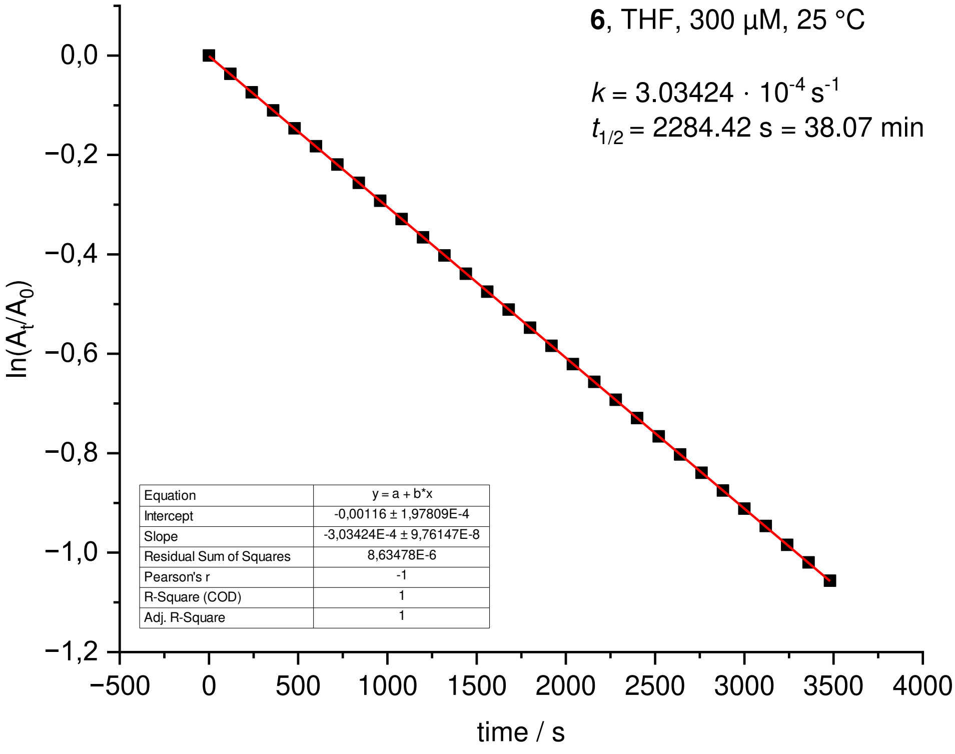

Figure S38: Determination of the thermal half-life $t_{1/2}$ of compound **6** (THF, 300 µM, 298 K) after irradiation with 385 nm (k = 3.03424 · 10^{-4} s^{-1}, $t_{1/2}$ = 38.07 min).

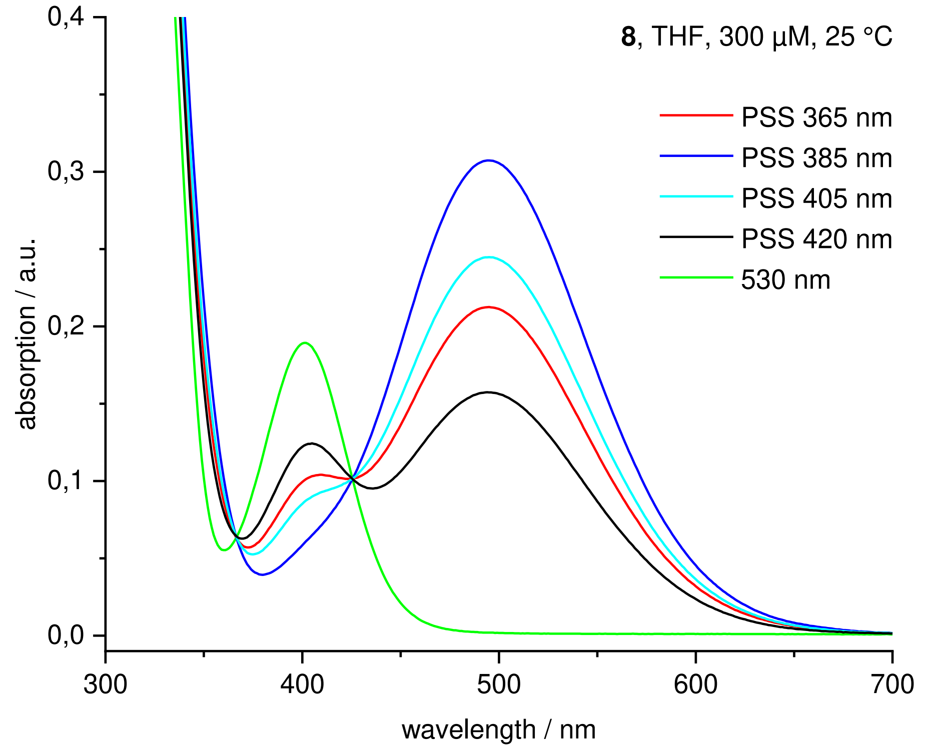

Figure S39: UV/vis spectra (THF, 300 µM, 298 K) of compound **8** after irradiation with 365 – 530 nm for 30 s at 298 K (λ_{max} (*Z*) = 401 nm, λ_{max} (*E*) = 495 nm).

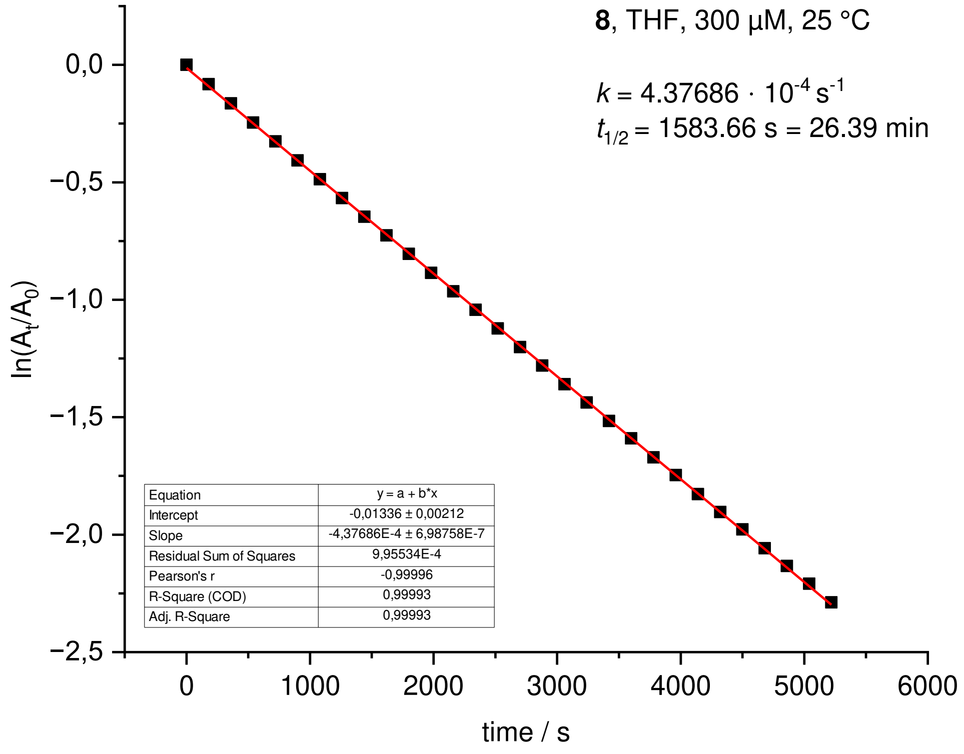

Figure S40: Determination of the thermal half-life $t_{1/2}$ of compound **8** (THF, 300 µM, 298 K) after irradiation with 385 nm (k = 4.37686 · 10^{-4} s^{-1}, $t_{1/2}$ = 26.39 min).

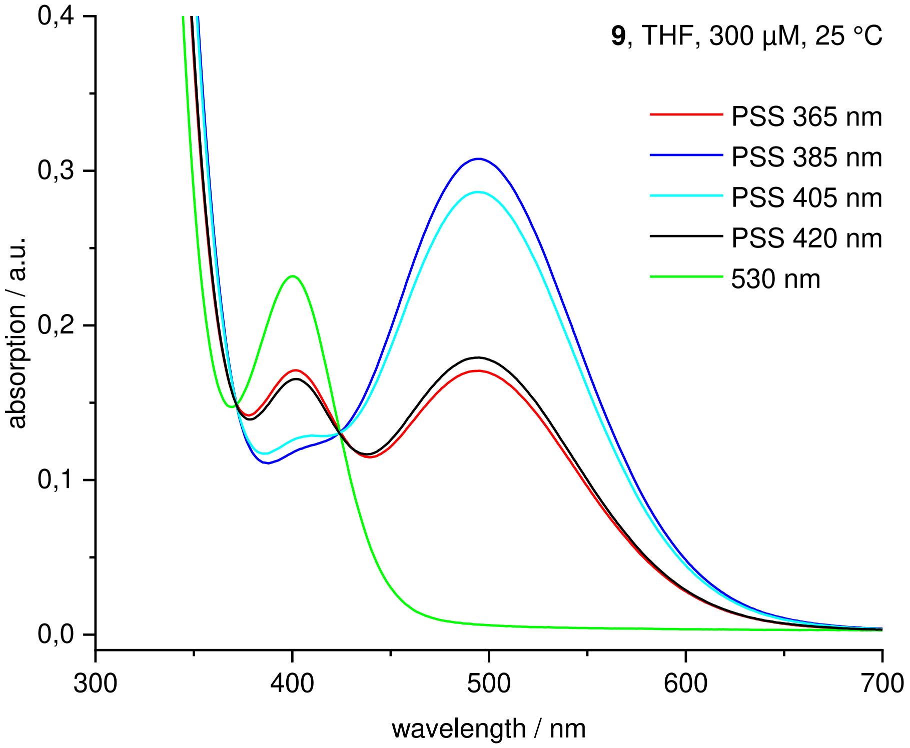

Figure S41: UV/vis spectra (THF, 300 µM, 298 K) of compound **9** after irradiation with 365 – 530 nm for 30 s at 298 K (λ_{max} (*Z*) = 400 nm, λ_{max} (*E*) = 495 nm).

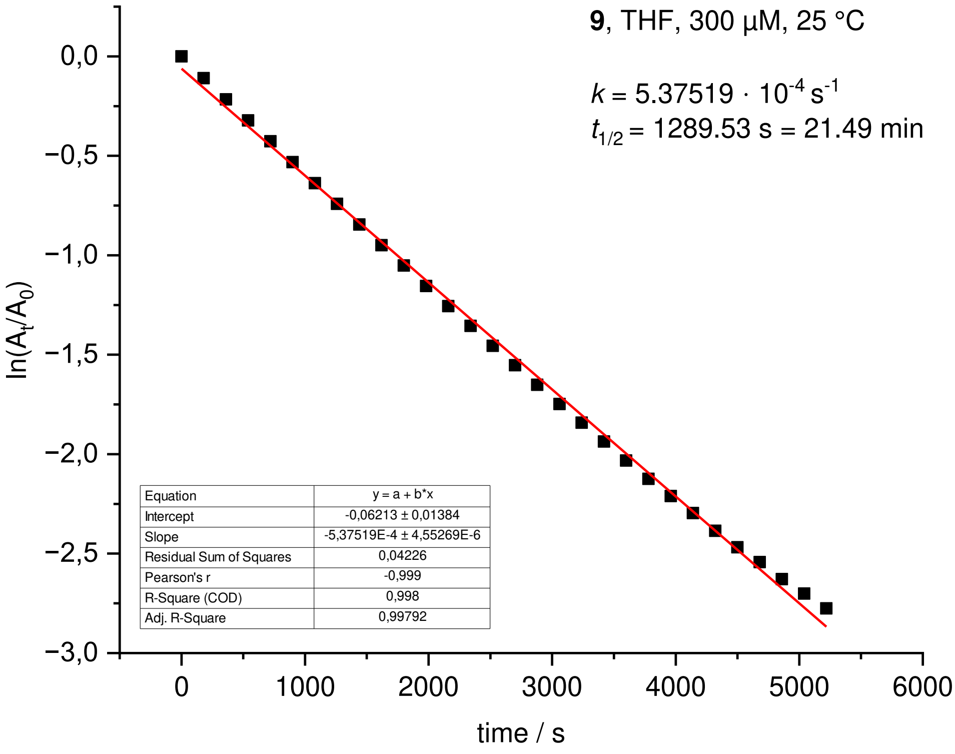

Figure S42: Determination of the thermal half-life $t_{1/2}$ of compound **9** (THF, 300 µM, 298 K) after irradiation with 385 nm ($k = 5.37519 \cdot 10^{-4}\ s^{-1}$, $t_{1/2}$ = 21.49 min).

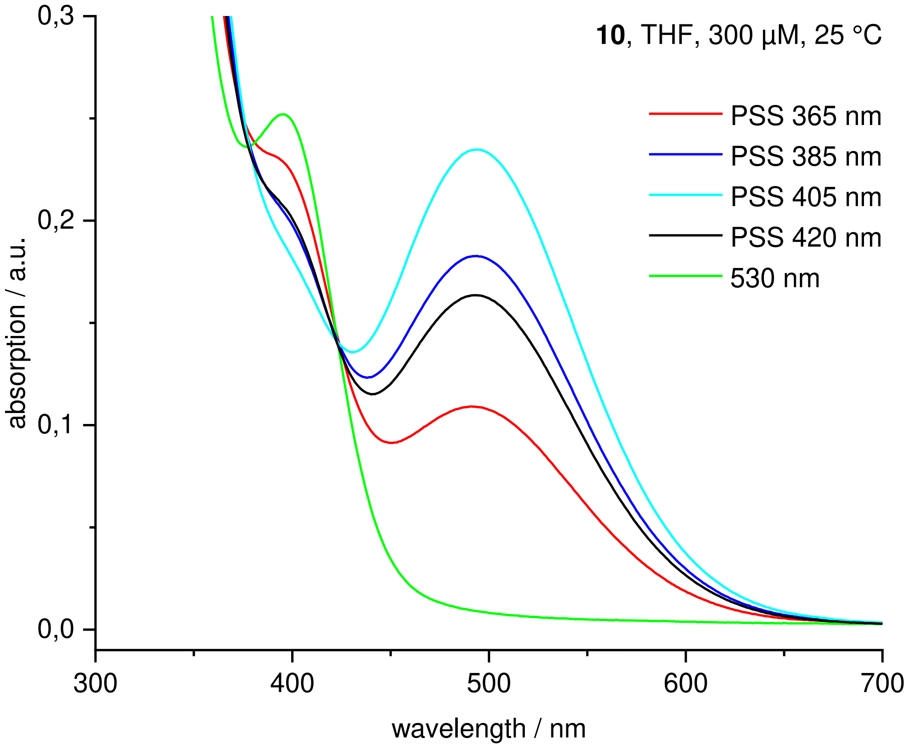

Figure S43: UV/vis spectra (THF, 300 µM, 298 K) of compound **10** after irradiation with 365 – 530 nm for 30 s at 298 K (λ_{max} (*Z*) = 396 nm, λ_{max} (*E*) = 494 nm).

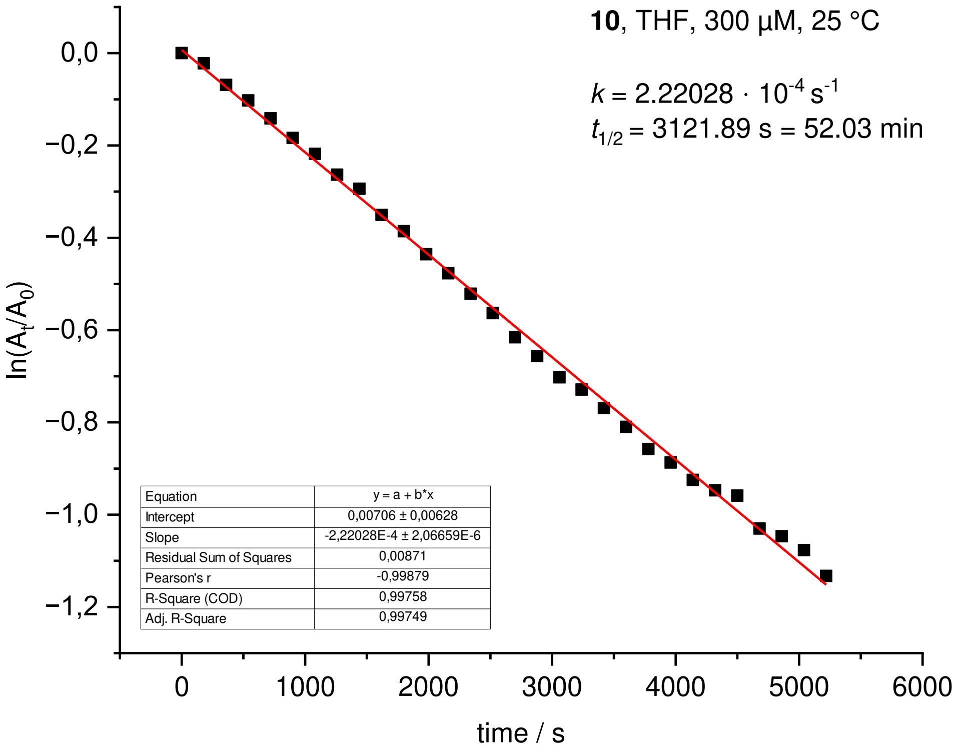

Figure S44: Determination of the thermal half-life $t_{1/2}$ of compound **10** (THF, 300 µM, 298 K) after irradiation with 385 nm ($k = 2.22028 \cdot 10^{-4}\ s^{-1}$, $t_{1/2}$ = 52.03 min).

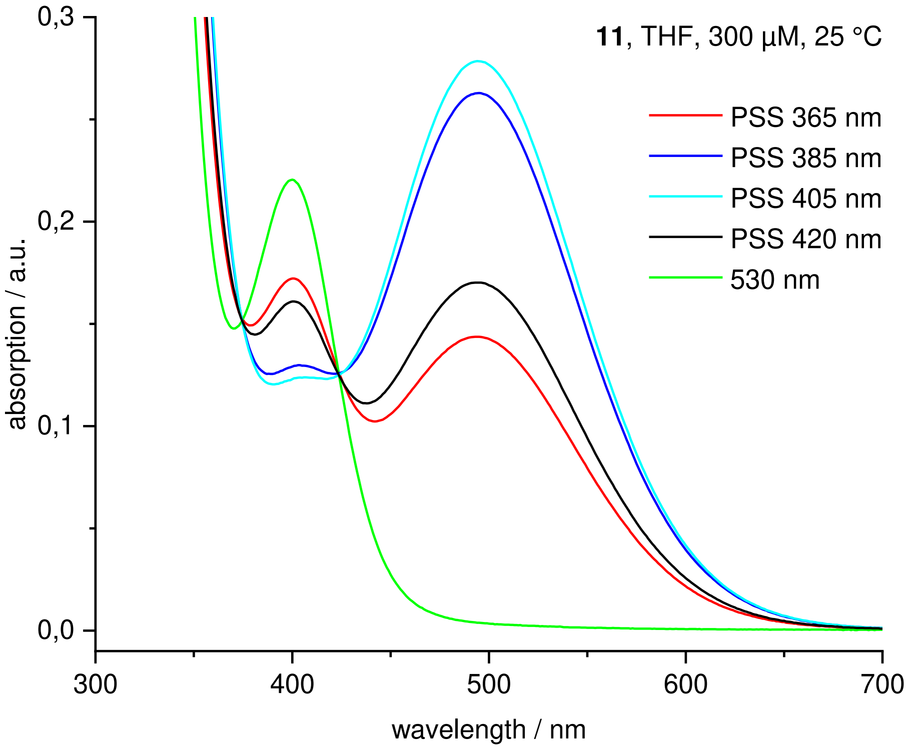

Figure S45: UV/vis spectra (THF, 300 µM, 298 K) of compound **11** after irradiation with 365 – 530 nm for 30 s at 298 K (λ_{max} (*Z*) = 400 nm, λ_{max} (*E*) = 494 nm).

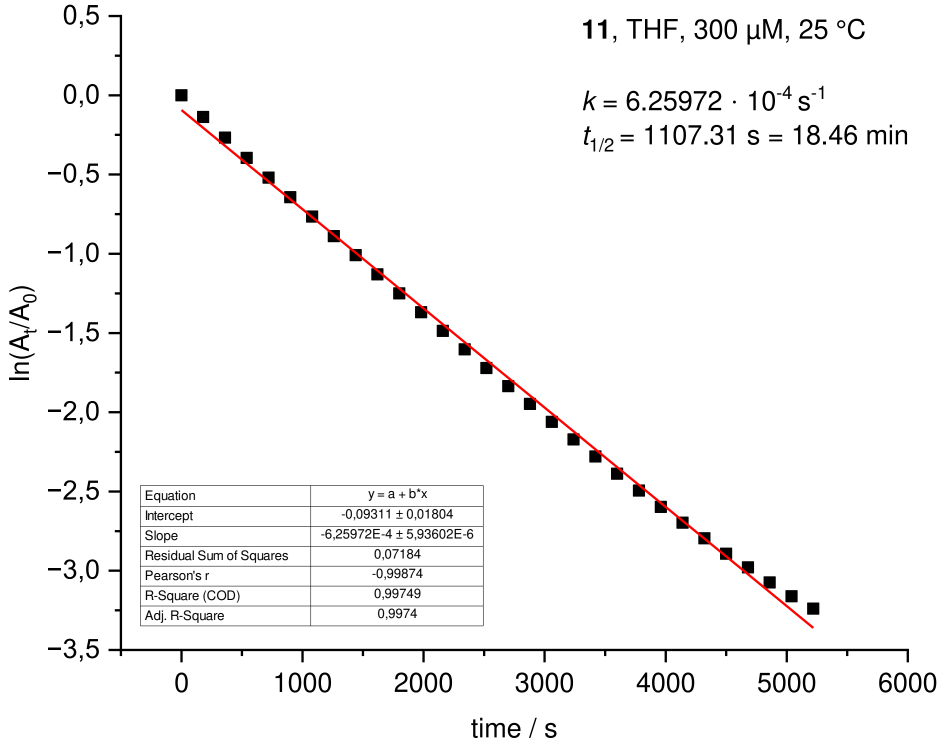

Figure S46: Determination of the thermal half-life $t_{1/2}$ of compound **11** (THF, 300 µM, 298 K) after irradiation with 405 nm (k = 6.25972 · 10^{-4} s^{-1}, $t_{1/2}$ = 18.46 min).

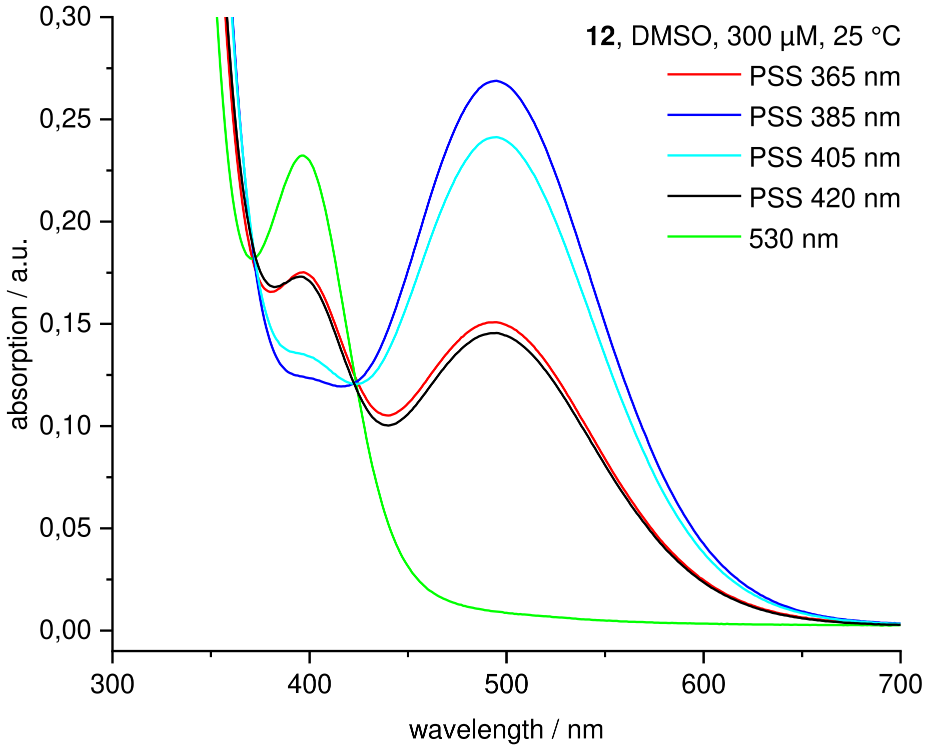

Figure S47: UV/vis spectra (DMSO, 300 µM, 298 K) of compound **12** after irradiation with 365 – 530 nm for 30 s at 298 K (λ_{max} (*Z*) = 397 nm, λ_{max} (*E*) = 495 nm).

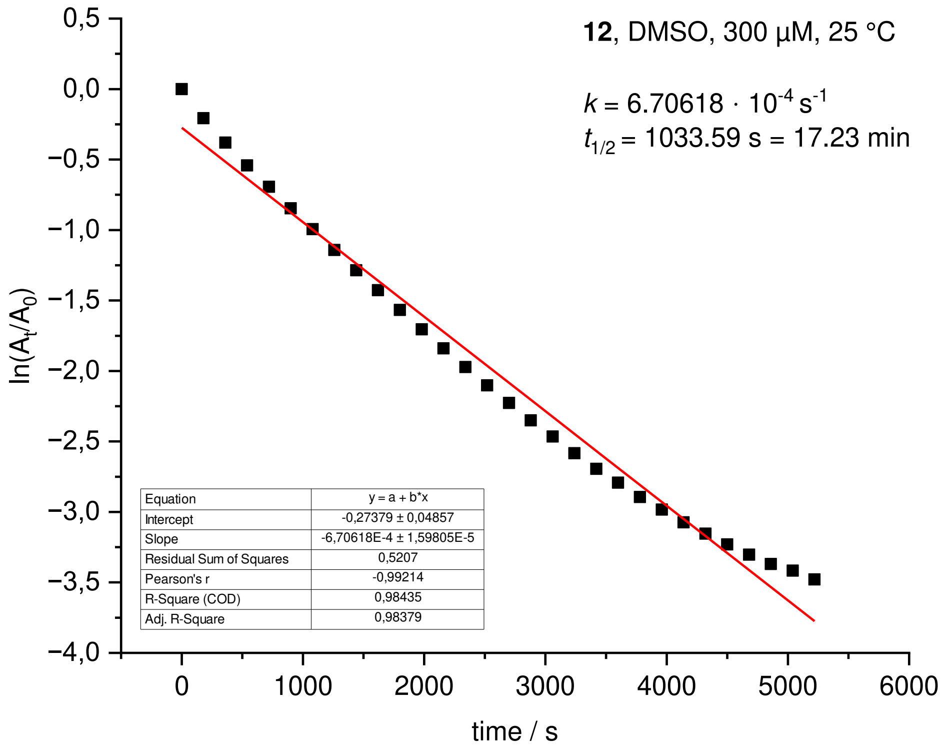

Figure S48: Determination of the thermal half-life $t_{1/2}$ of compound **12** (DMSO, 300 µM, 298 K) after irradiation with 385 nm (k = 6.70618 · 10^{-4} s^{-1}, $t_{1/2}$ = 17.23 min).

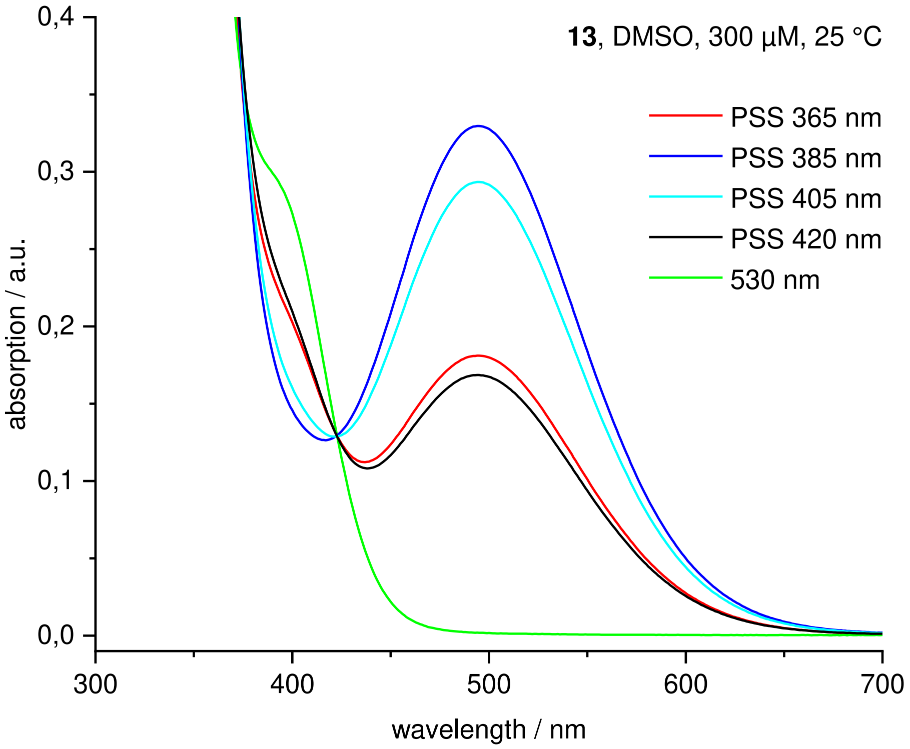

Figure S49: UV/vis spectra (DMSO, 300 µM, 298 K) of compound **13** after irradiation with 365 – 530 nm for 30 s at 298 K (λ_{max} (*Z*) = 401 nm, λ_{max} (*E*) = 494 nm).

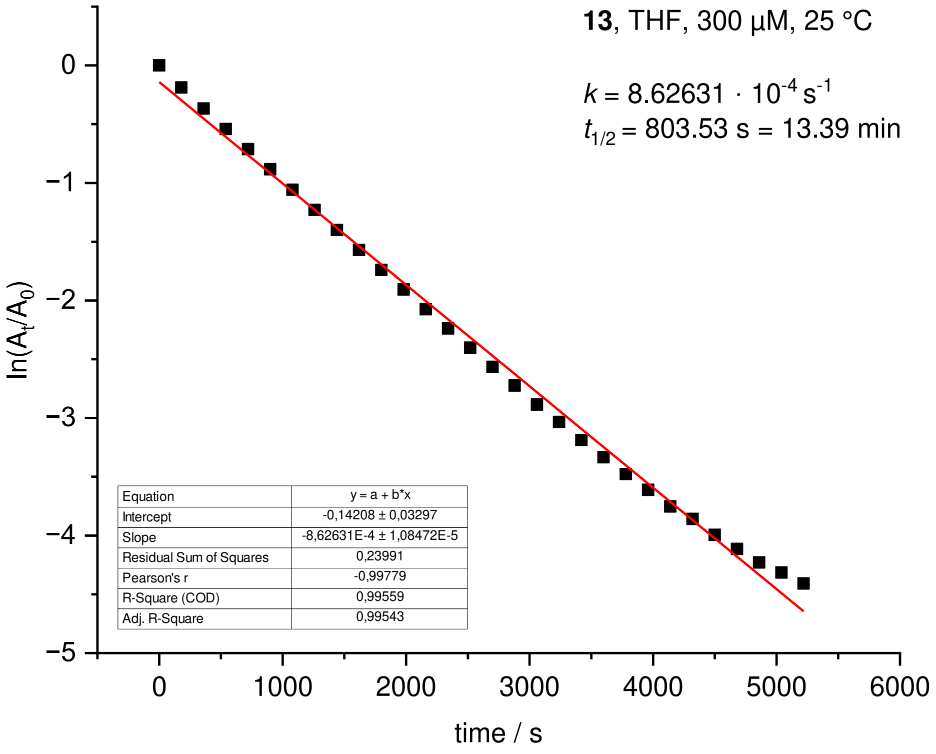

Figure S50: Determination of the thermal half-life $t_{1/2}$ of compound **13** (DMSO, 300 µM, 298 K) after irradiation with 385 nm ($k = 8.62631 \cdot 10^{-4}\ s^{-1}$, $t_{1/2}$ = 13.39 min).

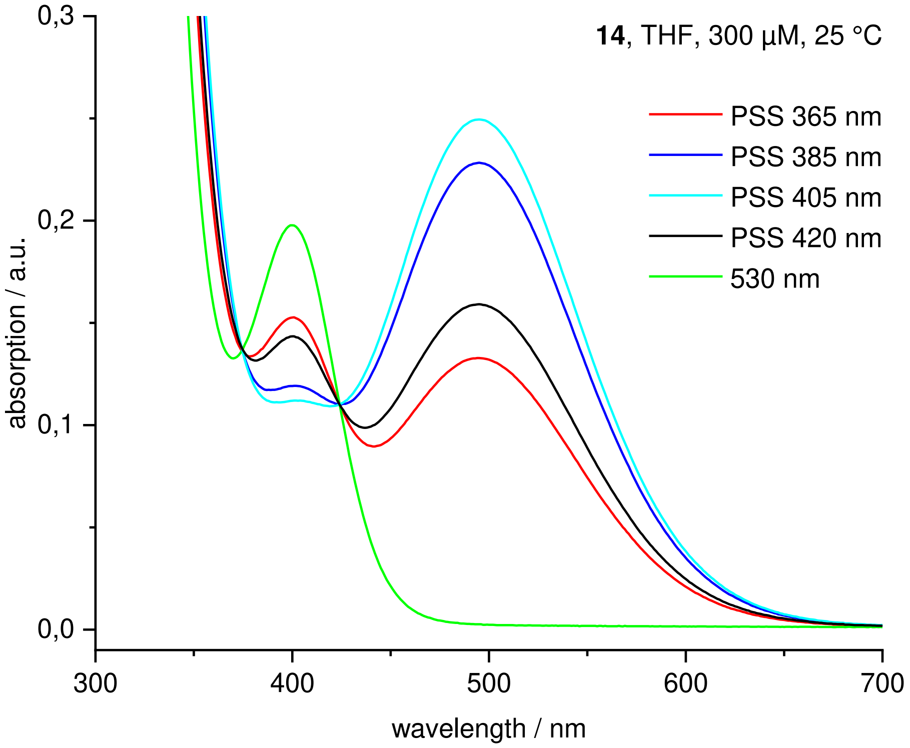

Figure S51: UV/vis spectra (THF, 300 µM, 298 K) of compound **14** after irradiation with 365 – 530 nm for 30 s at 298 K (λ_{max} (*Z*) = 400 nm, λ_{max} (*E*) = 495 nm).

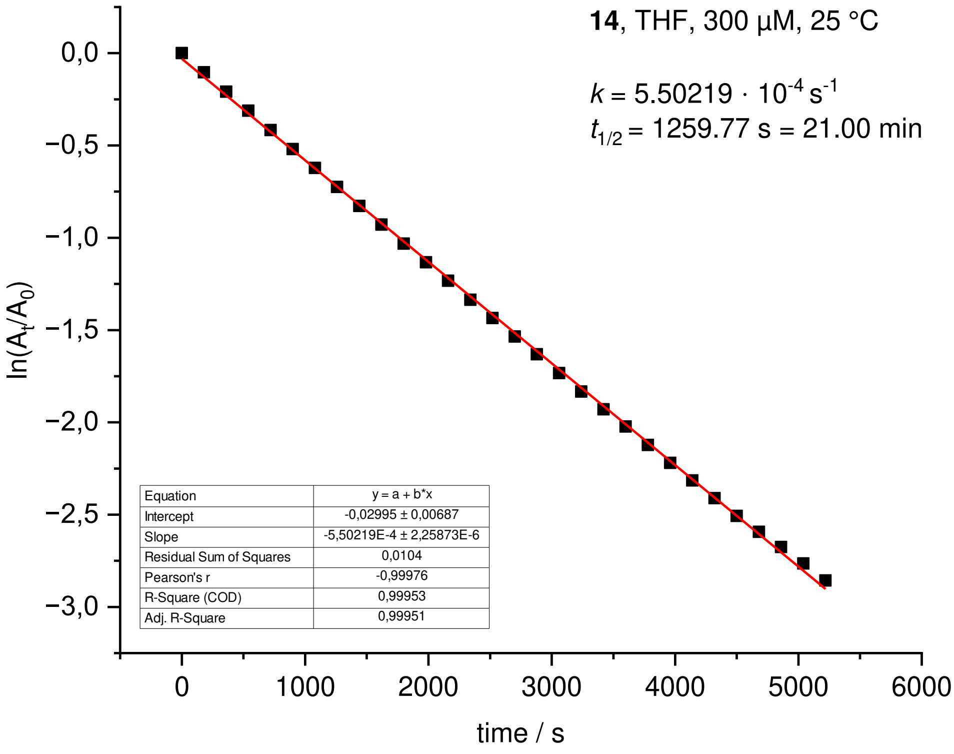

Figure S52: Determination of the thermal half-life $t_{1/2}$ of compound **14** (THF, 300 µM, 298 K) after irradiation with 405 nm (k = 5.50219 · 10^{-4} s^{-1}, $t_{1/2}$ = 21.00 min).

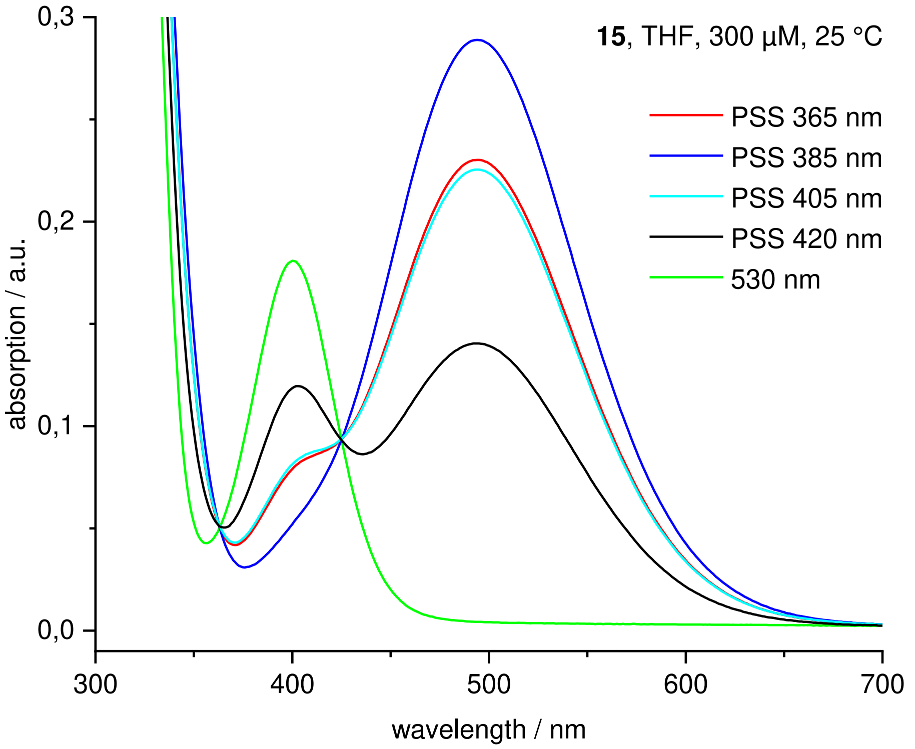

Figure S53: UV/vis spectra (THF, 300 µM, 298 K) of compound **15** after irradiation with 365 – 530 nm for 30 s at 298 K (λ_{max} (*Z*) = 400 nm, λ_{max} (*E*) = 494 nm).

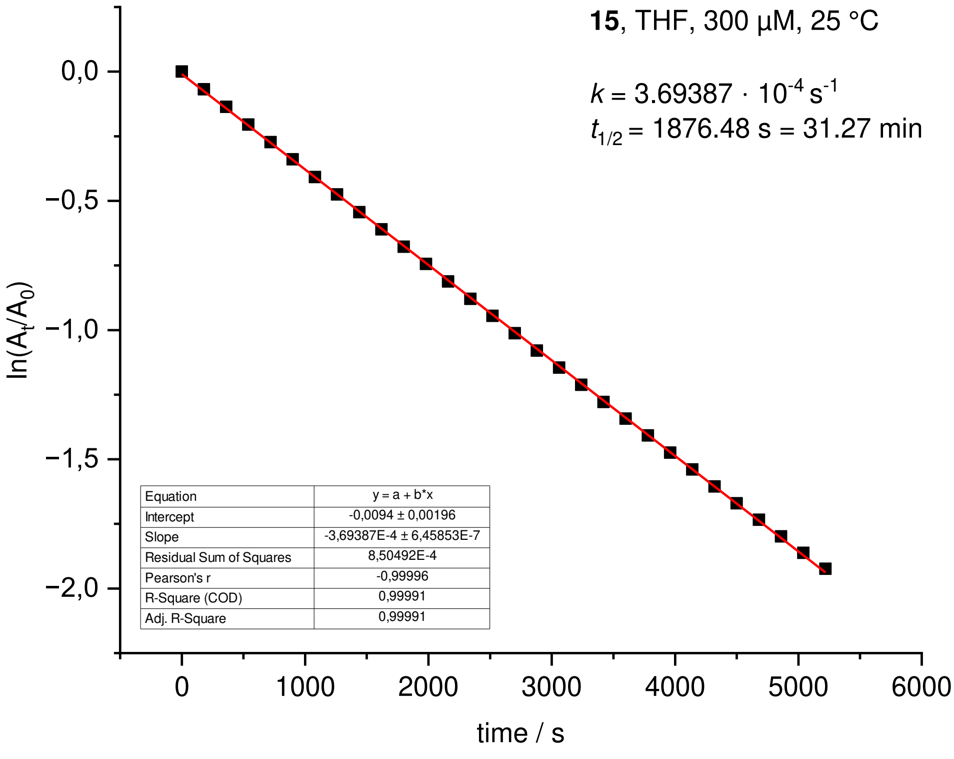

Figure S54: Determination of the thermal half-life $t_{1/2}$ of compound **15** (THF, 300 µM, 298 K) after irradiation with 385 nm ($k = 3.69387 \cdot 10^{-4}\ s^{-1}$, $t_{1/2} = 31.27$ min).

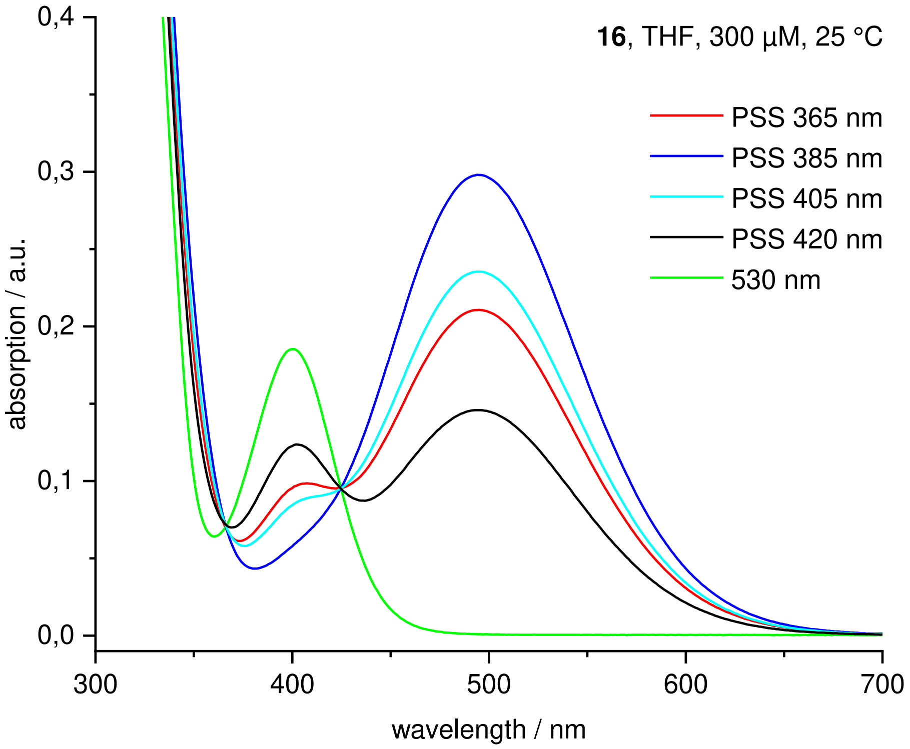

Figure S55: UV/vis spectra (THF, 300 µM, 298 K) of compound **16** after irradiation with 365 – 530 nm for 30 s at 298 K (λ_{max} (*Z*) = 400 nm, λ_{max} (*E*) = 495 nm).

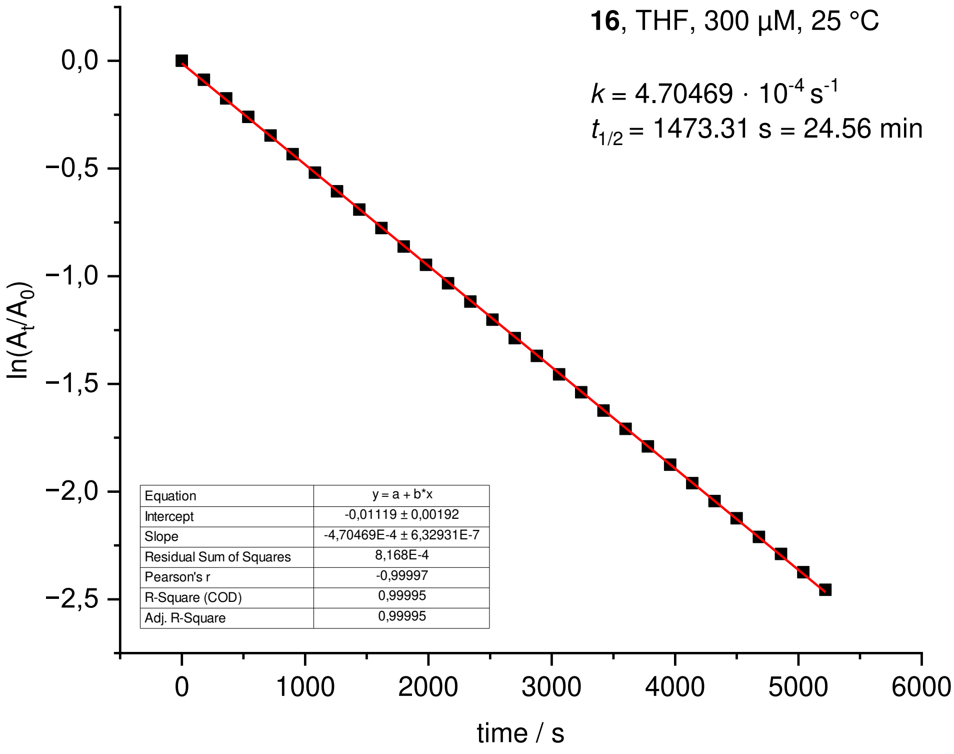

Figure S56: Determination of the thermal half-life $t_{1/2}$ of compound **16** (THF, 300 µM, 298 K) after irradiation with 385 nm ($k = 4.70469 \cdot 10^{-4}$ s^{-1}, $t_{1/2}$ = 24.56 min).

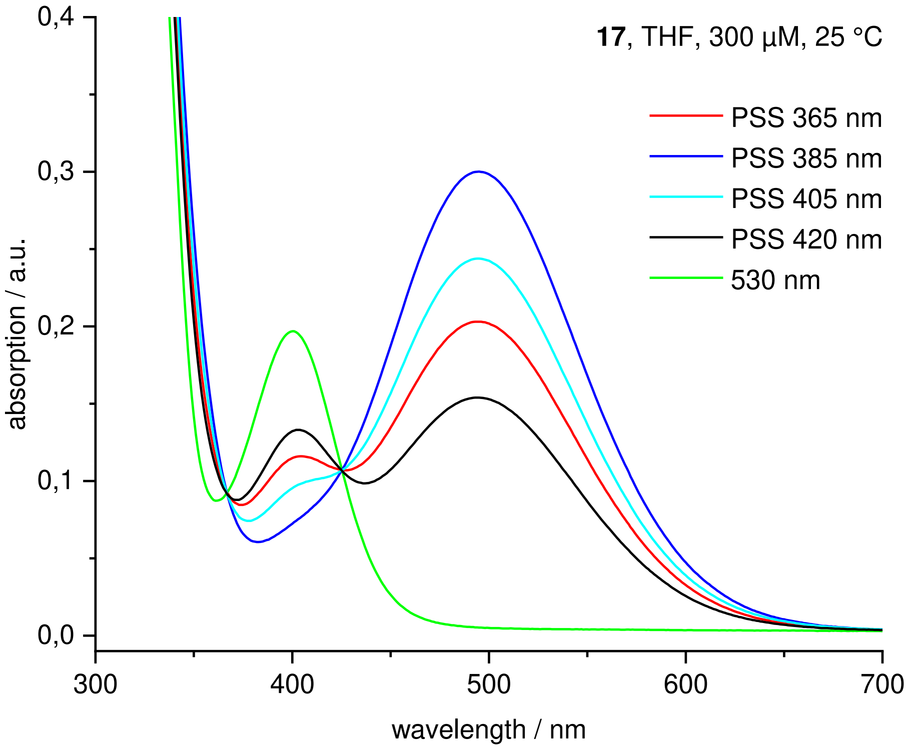

Figure S57: UV/vis spectra (THF, 300 µM, 298 K) of compound **17** after irradiation with 365 – 530 nm for 30 s at 298 K (λ_{max} (Z) = 400 nm, λ_{max} (E) = 495 nm).

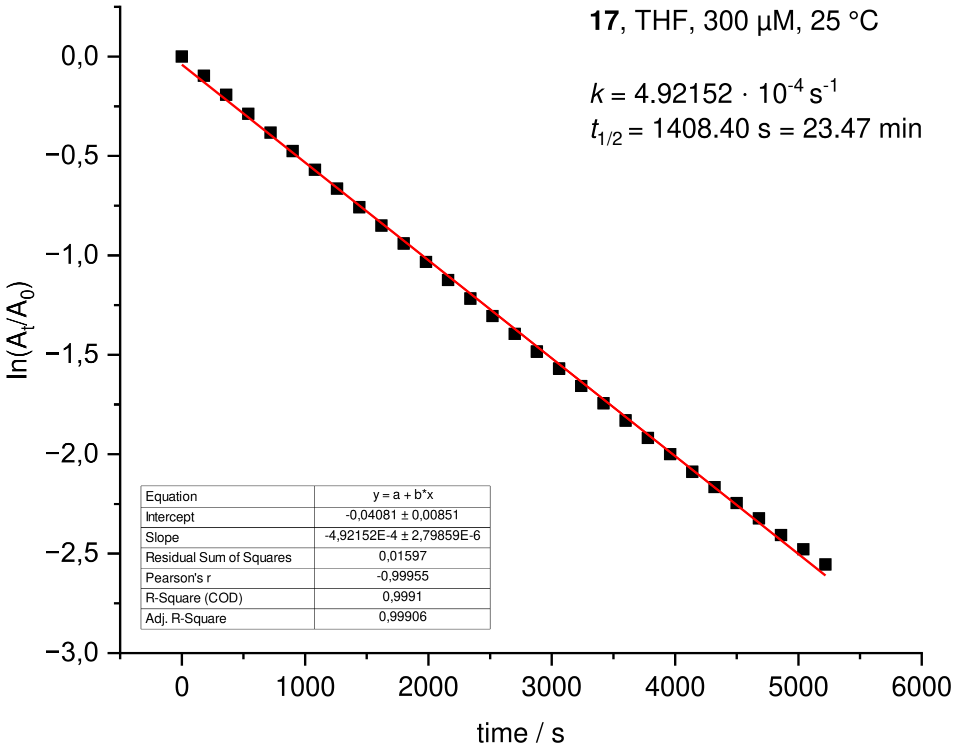

Figure S58: Determination of the thermal half-life $t_{1/2}$ of compound **17** (THF, 300 µM, 298 K) after irradiation with 385 nm (k = 4.92152 · 10^{-4} s^{-1}, $t_{1/2}$ = 23.47 min).

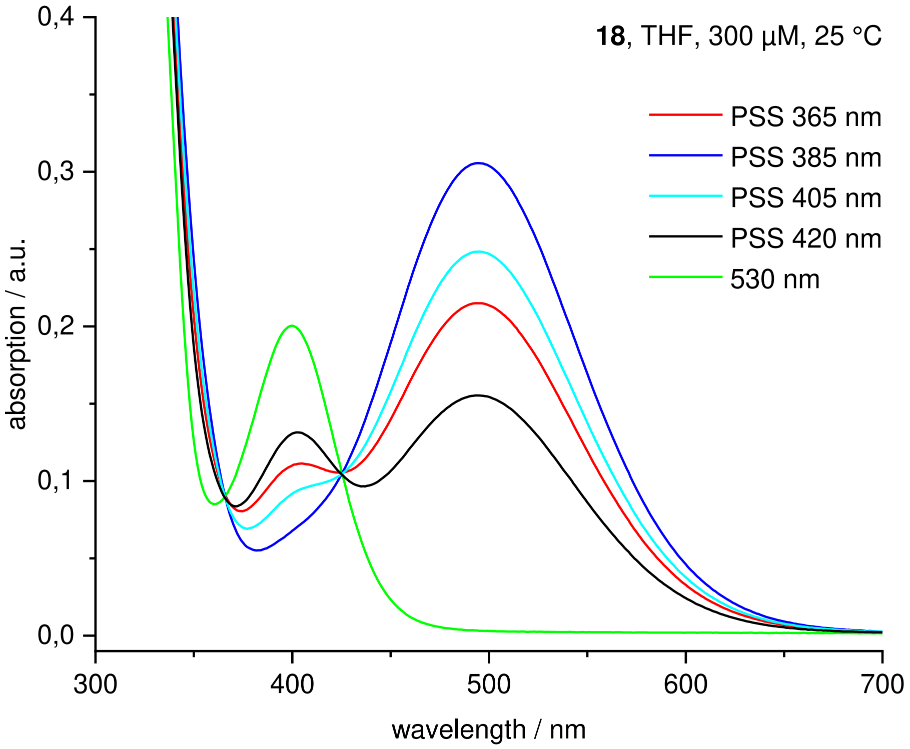

Figure S59: UV/vis spectra (THF, 300 µM, 298 K) of compound **18** after irradiation with 365 – 530 nm for 30 s at 298 K (λ_{max} (*Z*) = 400 nm, λ_{max} (*E*) = 495 nm).

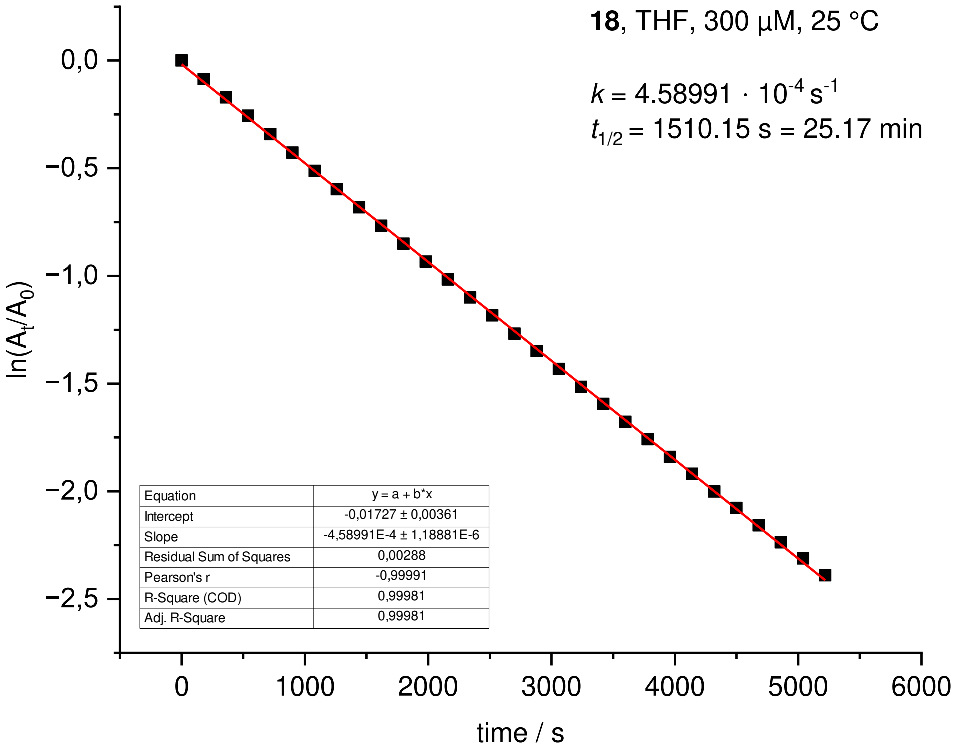

Figure S60: Determination of the thermal half-life $t_{1/2}$ of compound **18** (THF, 300 µM, 298 K) after irradiation with 385 nm (k = 4.58991 · 10^{-4} s^{-1}, $t_{1/2}$ = 25.17 min).

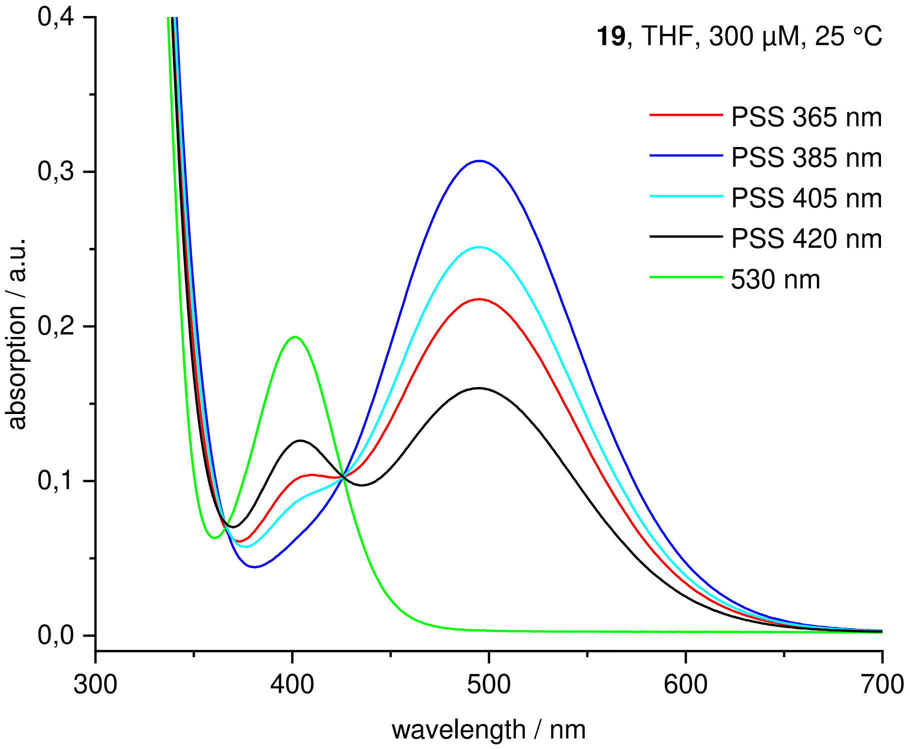

Figure S61: UV/vis spectra (THF, 300 µM, 298 K) of compound **19** after irradiation with 365 – 530 nm for 30 s at 298 K (λ_{max} (*Z*) = 401 nm, λ_{max} (*E*) = 495 nm).

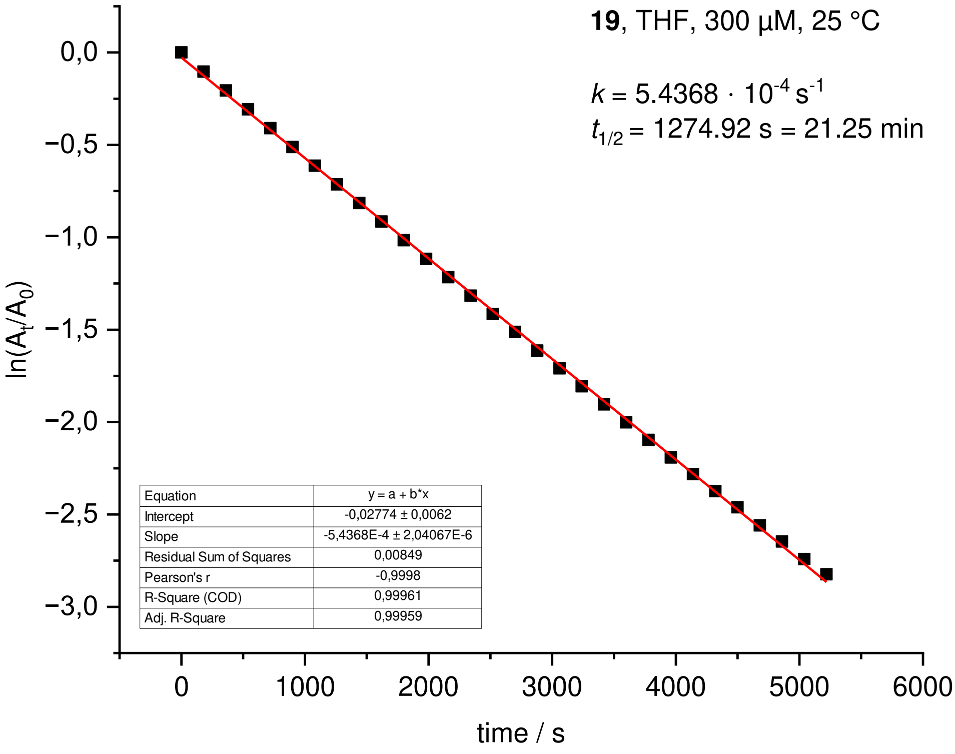

Equation	y = a + b*x
Intercept	-0,02774 ± 0,0062
Slope	-5,4368E-4 ± 2,04067E-6
Residual Sum of Squares	0,00849
Pearson's r	-0,9998
R-Square (COD)	0,99961
Adj. R-Square	0,99959

Figure S62: Determination of the thermal half-life $t_{1/2}$ of compound **19** (THF, 300 µM, 298 K) after irradiation with 385 nm (k = 5.4368 · 10^{-4} s^{-1}, $t_{1/2}$ = 21.25 min).

S 4.2 Photostationary states

To suppress undesired *E* → *Z* re-isomerization due to thermal relaxation, 1.00 mg of each compounds **5**, **6**, **8** and **15** – **19** were dissolved in THF-d_8 and irradiated for 30 s at 273 K with the corresponding optimum wavelength for obtaining the photostationary state (see S 4.1). The following NMR spectra were also measured at 273 K. Due to the precipitation of the respective *E* isomers in THF-d_8, 1.00 mg of each compounds **9** – **14** were dissolved in DMSO-d_6 and irradiated for 30 s at 298 K with the optimum wavelengths for obtaining the photostationary state (see chapter S 4.1). The corresponding NMR spectra were measured at 298 K.

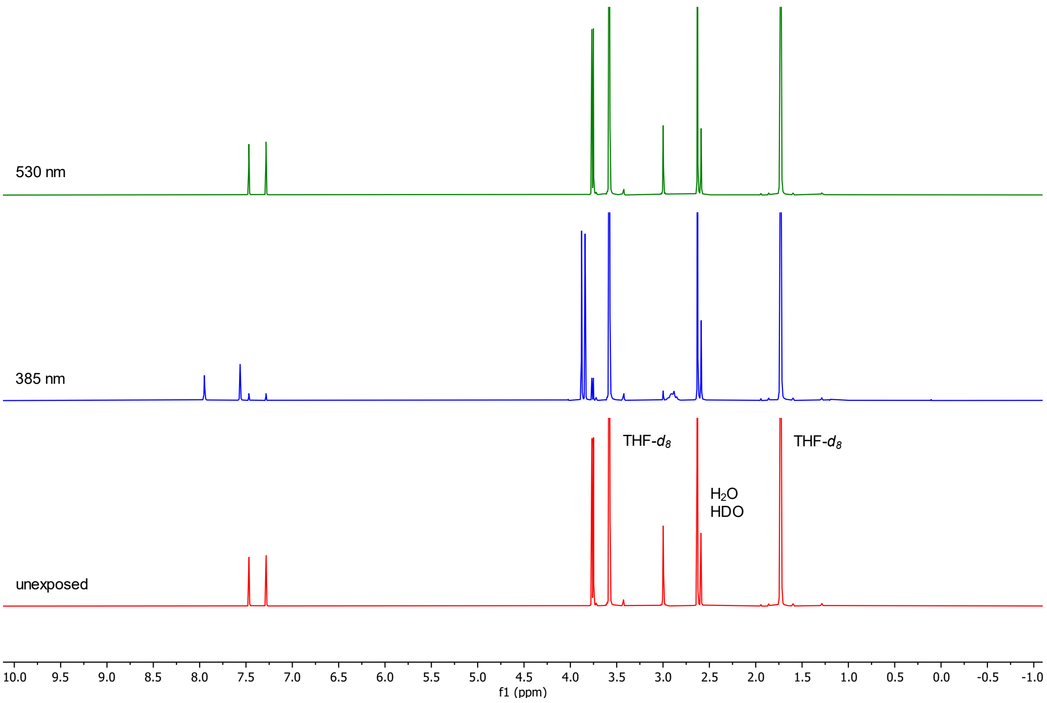

Figure S63: ^{1}H NMR spectra (500 MHz, THF-*d*8, 273 K) of compound **5** (red), after irradiation for 30 s at 273 K with 385 nm (blue) and 530 nm (green).

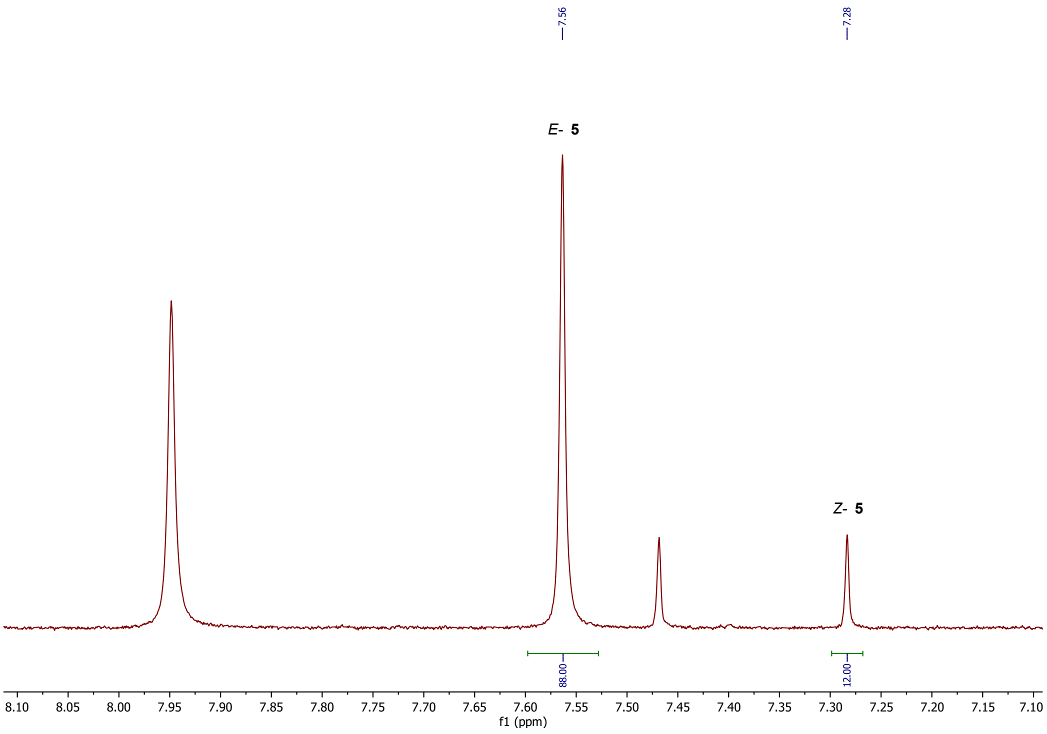

Figure S64: Excerpt of the ^{1}H NMR spectrum (500 MHz, THF-*d*8, 273 K) of the photostationary state of compound **5** after irradiation with 385 nm for 30 s at 273 K and integration of the corresponding signals of the *Z* and *E* isomers (*Z*/*E*, 12:88).

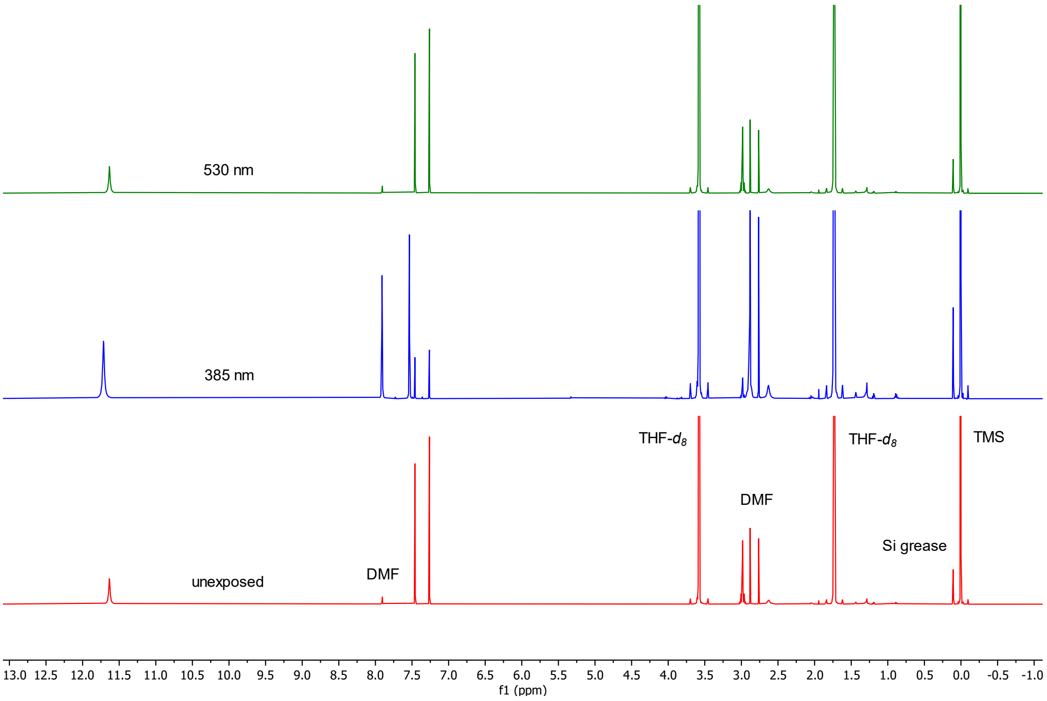

Figure S65: ^{1}H NMR spectra (600 MHz, THF-d_8, 273 K) of compound **6** (red), after irradiation for 30 s at 273 K with 385 nm (blue) and 530 nm (green).

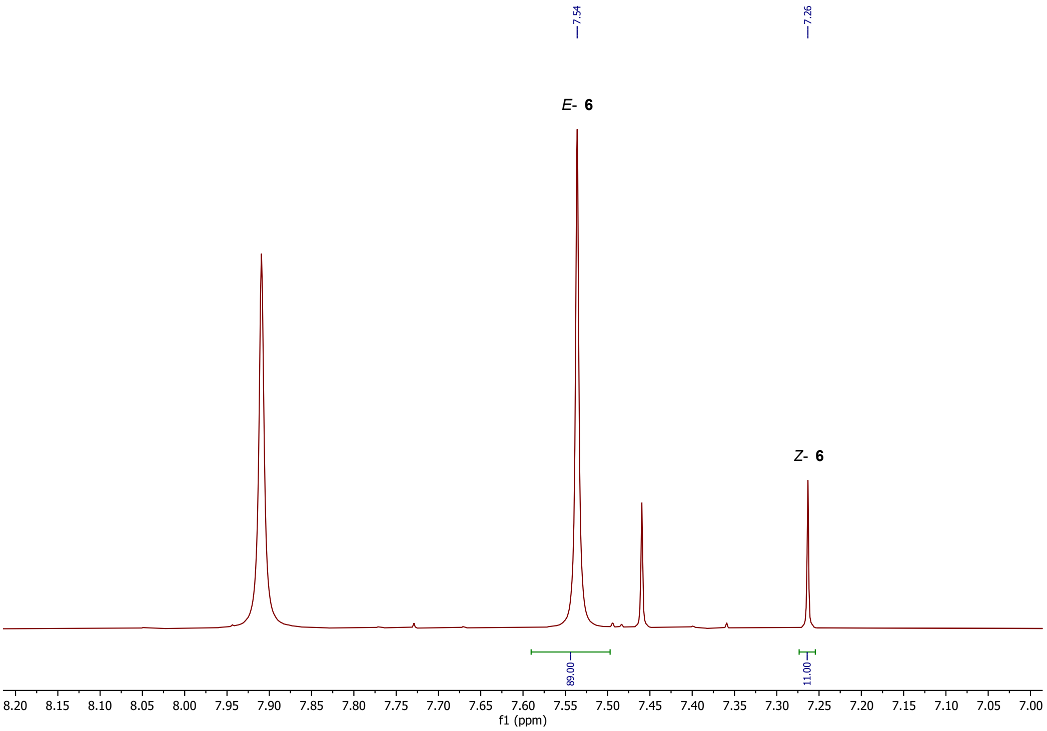

Figure S66: Excerpt of the ^{1}H NMR spectrum (600 MHz, THF-d_8, 273 K) of the photostationary state of compound **6** after irradiation with 385 nm for 30 s at 273 K and integration of the corresponding signals of the *Z* and *E* isomers (*Z*/*E*, 11:89).

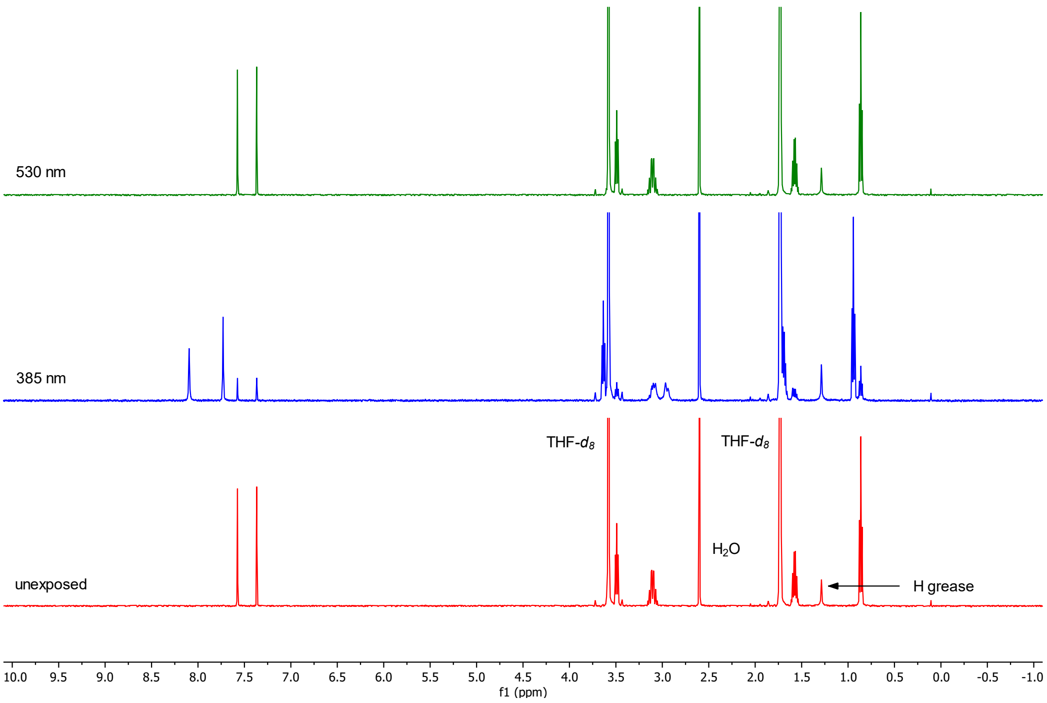

Figure S67: ^{1}H NMR spectra (500 MHz, THF-d_8, 273 K) of compound **8** (red), after irradiation for 30 s at 273 K with 385 nm (blue) and 530 nm (green).

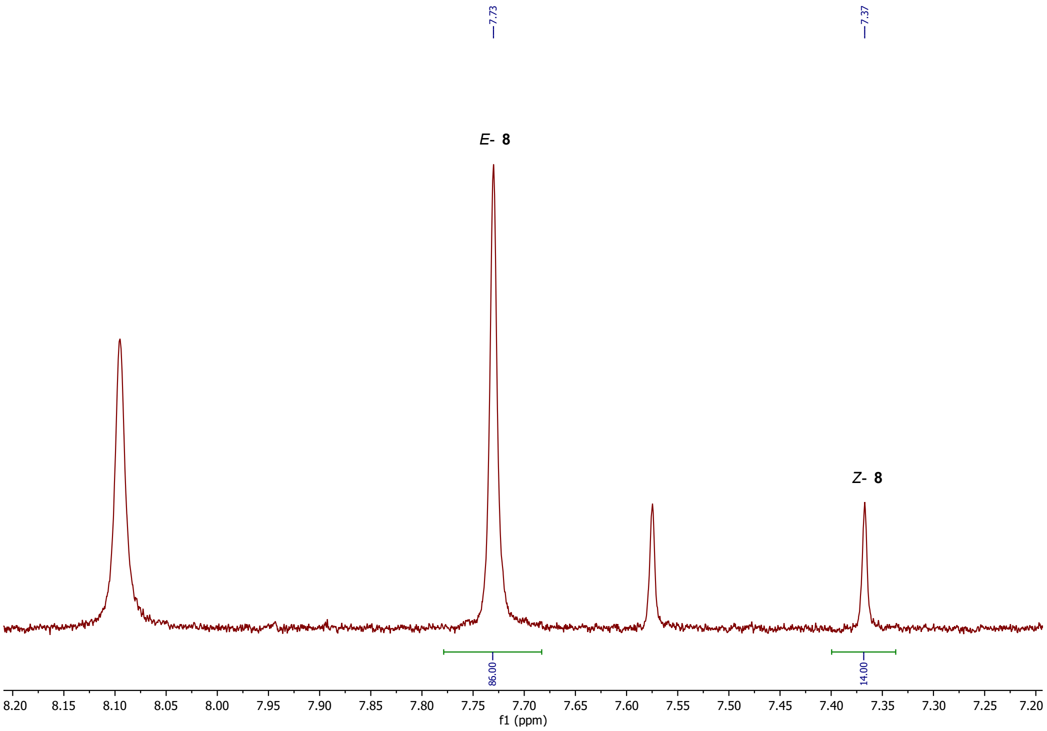

Figure S68: Excerpt of the ^{1}H NMR spectrum (500 MHz, THF-d_8, 273 K) of the photostationary state of compound **8** after irradiation with 385 nm for 30 s at 273 K and integration of the corresponding signals of the *Z* and *E* isomers (*Z*/*E*, 14:86).

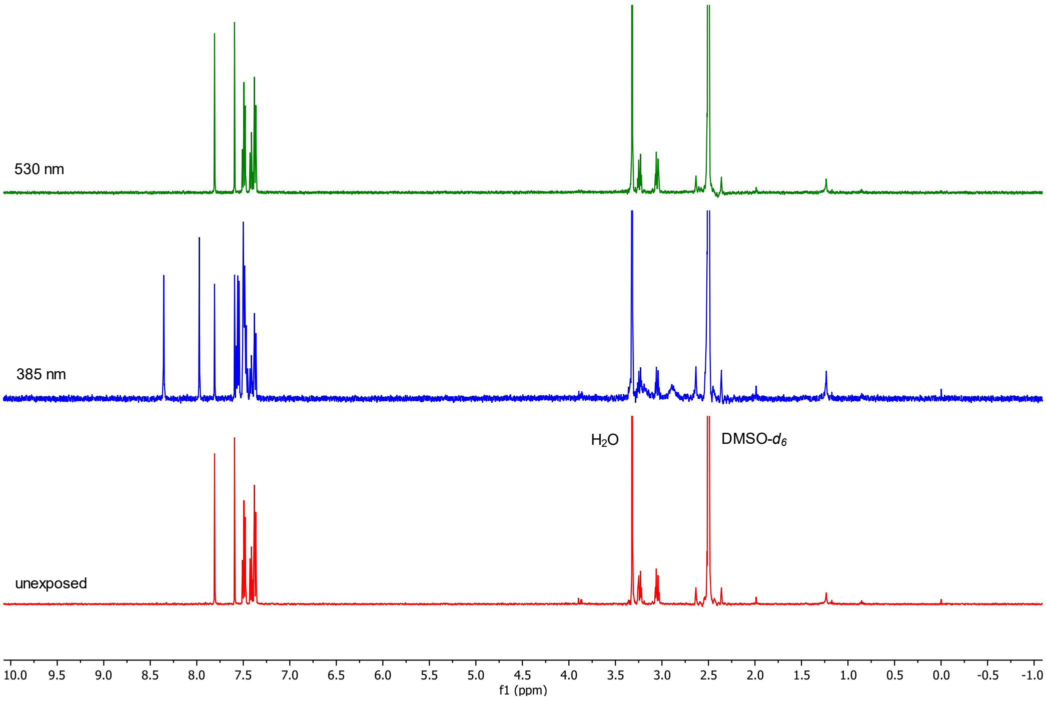

Figure S69: ^{1}H NMR spectra (500 MHz, DMSO-*d*$_6$, 298 K) of compound **9** (red), after irradiation for 30 s at 298 K with 385 nm (blue) and 530 nm (green).

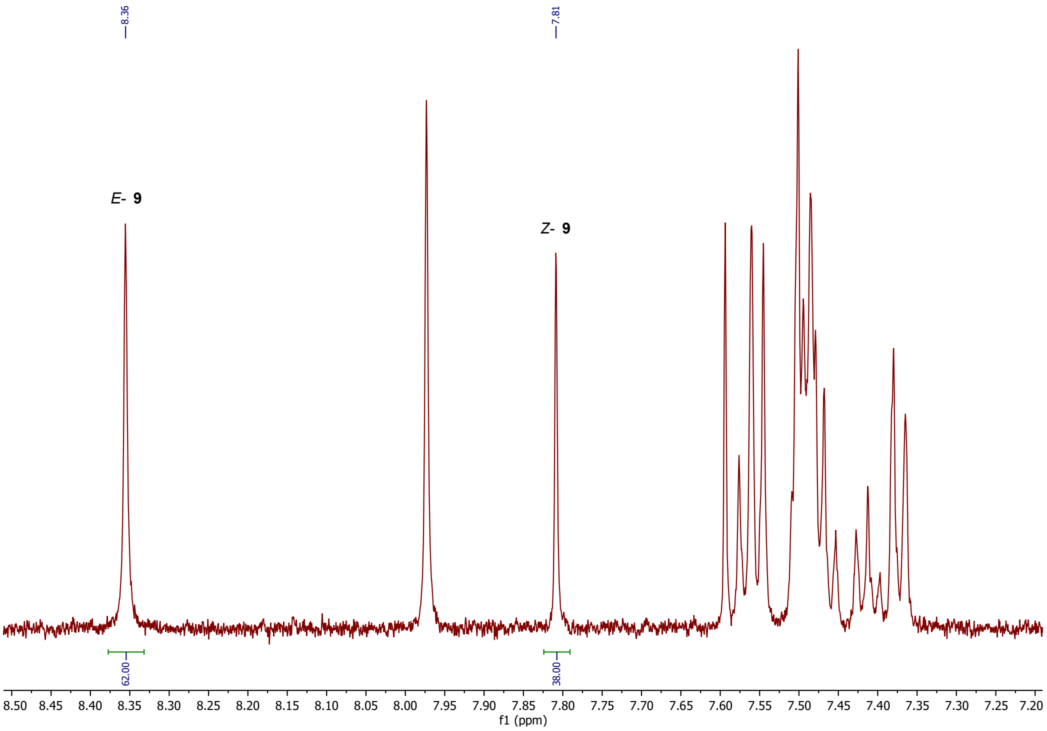

Figure S70: Excerpt of the ^{1}H NMR spectrum (500 MHz, DMSO-*d*$_6$, 298 K) of the photostationary state of compound **9** after irradiation with 385 nm for 30 s at 298 K and integration of the corresponding signals of the *Z* and *E* isomers (*Z*/*E*, 38:62).

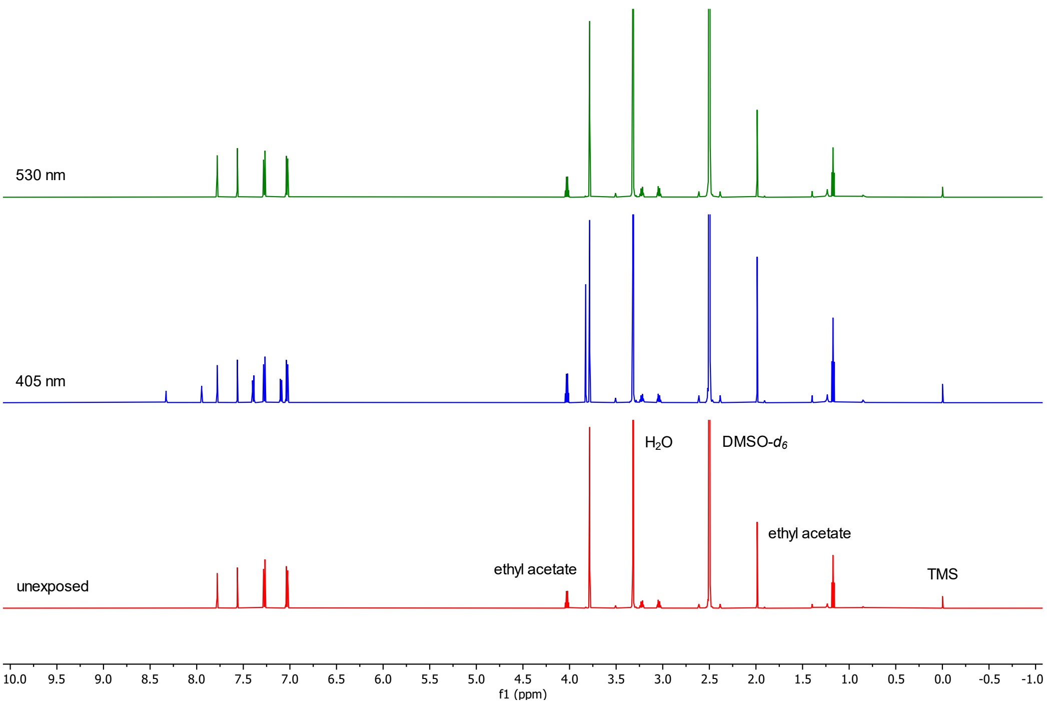

Figure S71: ^{1}H NMR spectra (600 MHz, DMSO-*d*6, 298 K) of compound **10** (red), after irradiation for 30 s at 298 K with 405 nm (blue) and 530 nm (green).

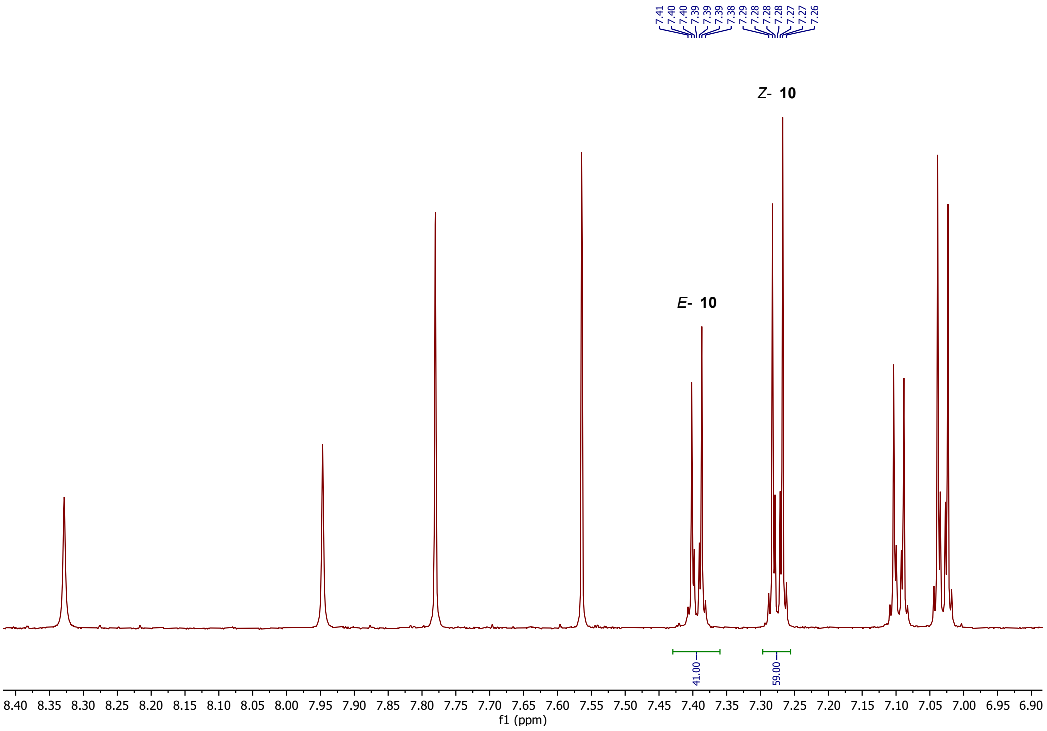

Figure S72: Excerpt of the ^{1}H NMR spectrum (600 MHz, DMSO-*d*6, 298 K) of the photostationary state of compound **10** after irradiation with 405 nm for 30 s at 298 K and integration of the corresponding signals of the *Z* and *E* isomers (*Z*/*E*, 59:41).

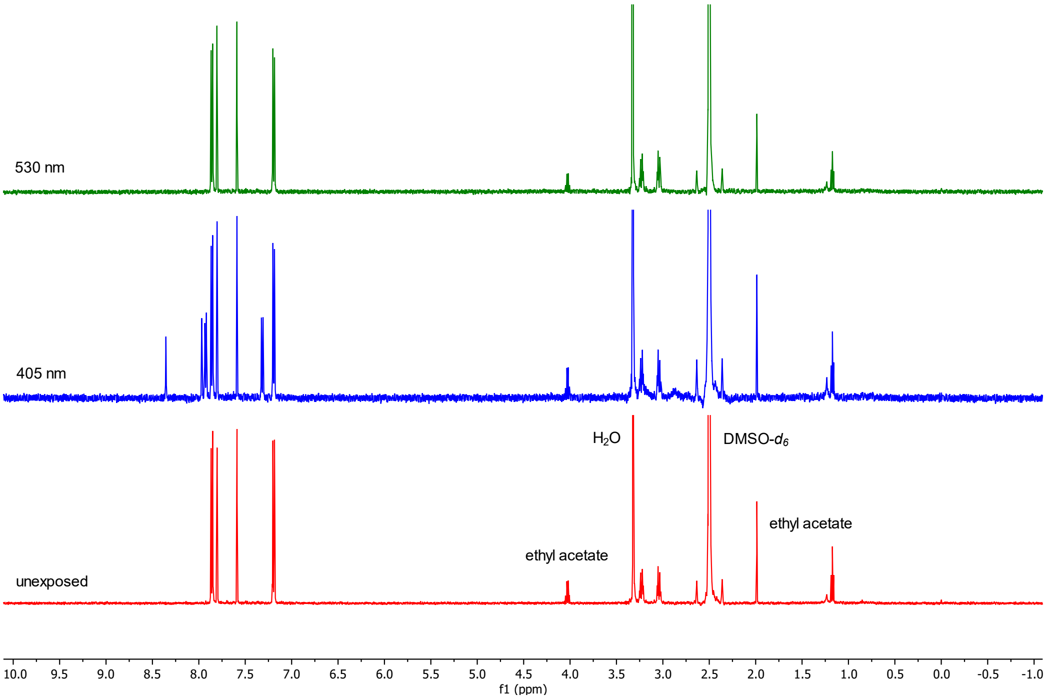

Figure S73: ^{1}H NMR spectra (500 MHz, DMSO-d_6, 298 K) of compound **11** (red), after irradiation for 30 s at 298 K with 405 nm (blue) and 530 nm (green).

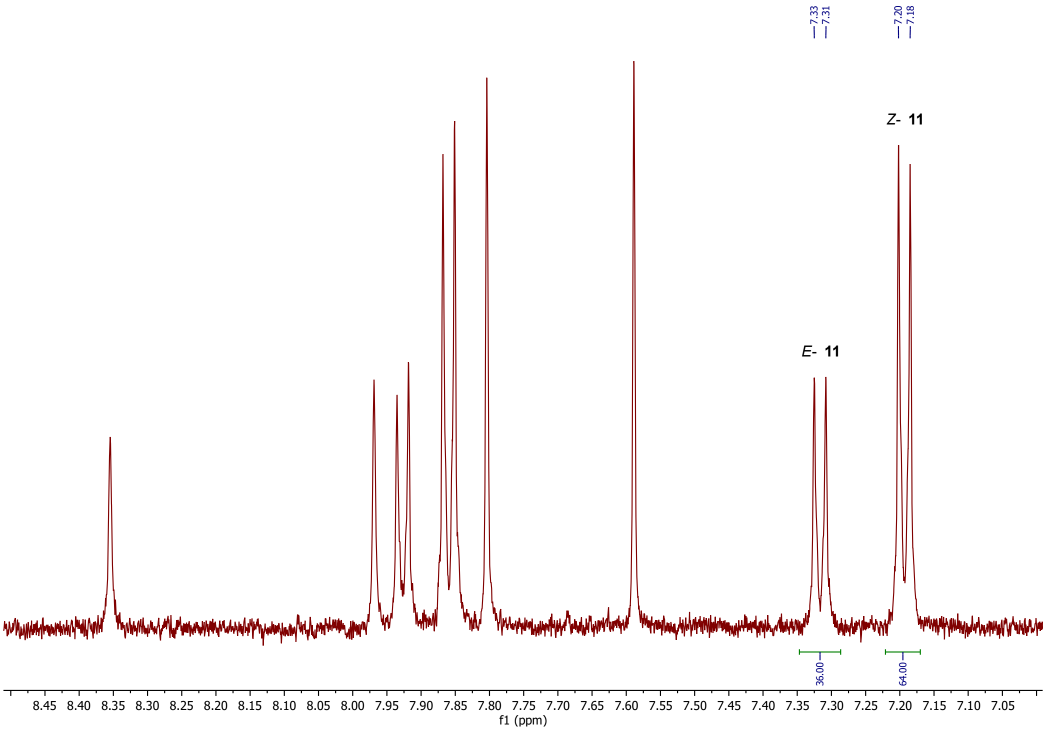

Figure S74: Excerpt of the ^{1}H NMR spectrum (500 MHz, DMSO-d_6, 298 K) of the photostationary state of compound **11** after irradiation with 405 nm for 30 s at 298 K and integration of the corresponding signals of the *Z* and *E* isomers (*Z*/*E*, 64:36).

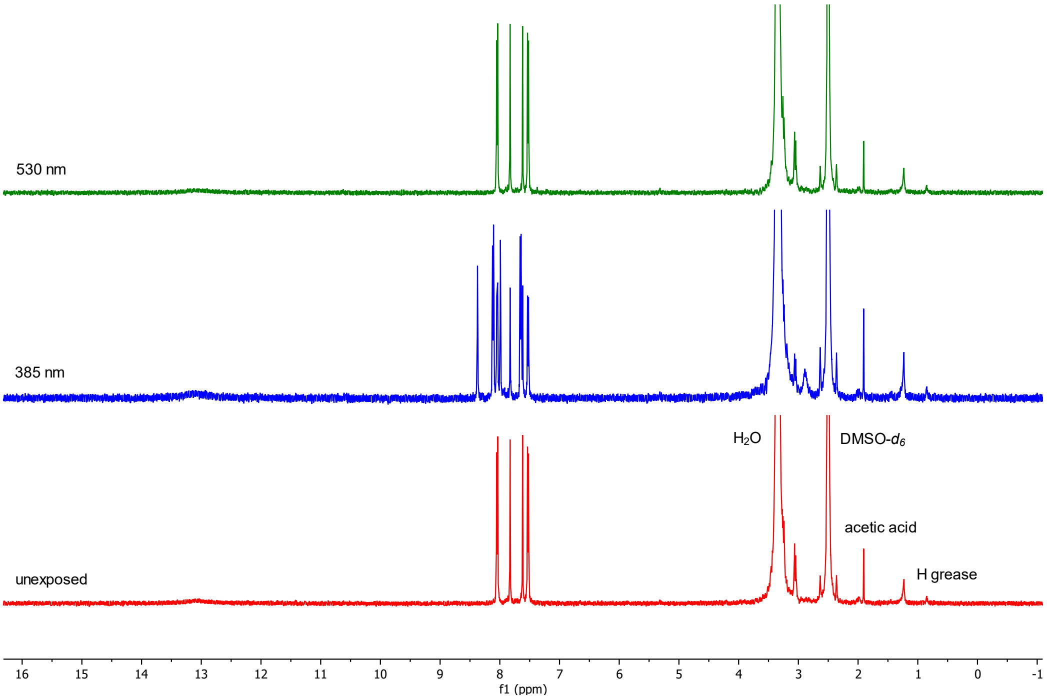

Figure S75: ^{1}H NMR spectra (500 MHz, DMSO-d_6, 298 K) of compound **12** (red), after irradiation for 30 s at 298 K with 385 nm (blue) and 530 nm (green).

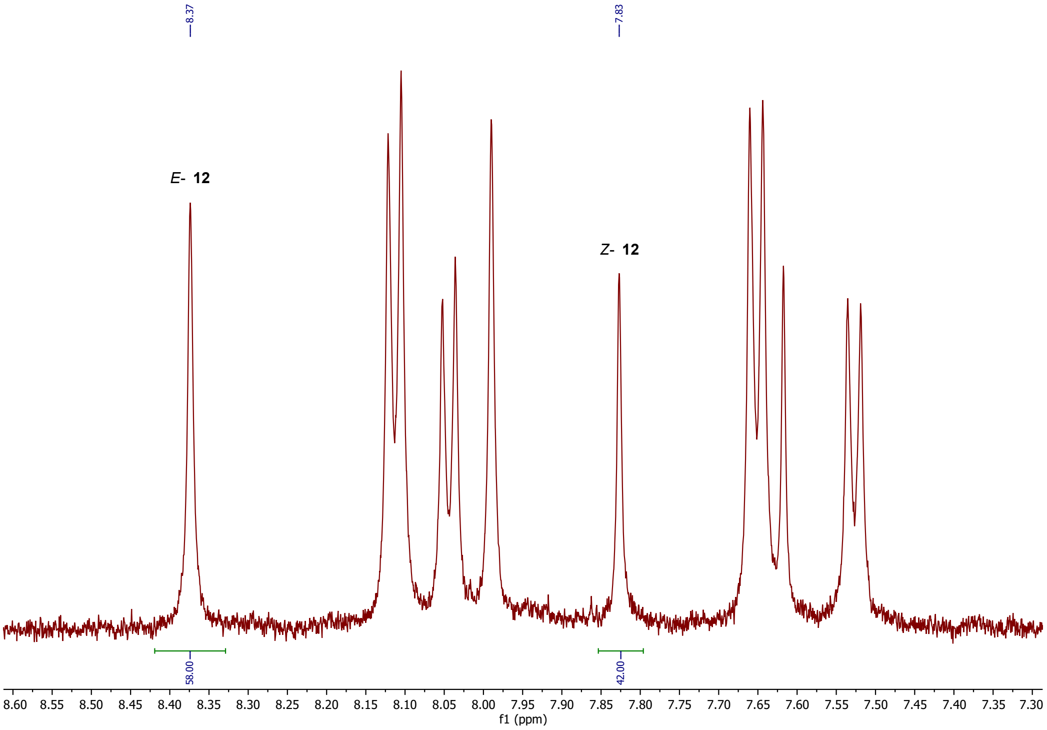

Figure S76: Excerpt of the ^{1}H NMR spectrum (500 MHz, DMSO-d_6, 298 K) of the photostationary state of compound **12** after irradiation with 385 nm for 30 s at 298 K and integration of the corresponding signals of the *Z* and *E* isomers (*Z*/*E*, 42:58).

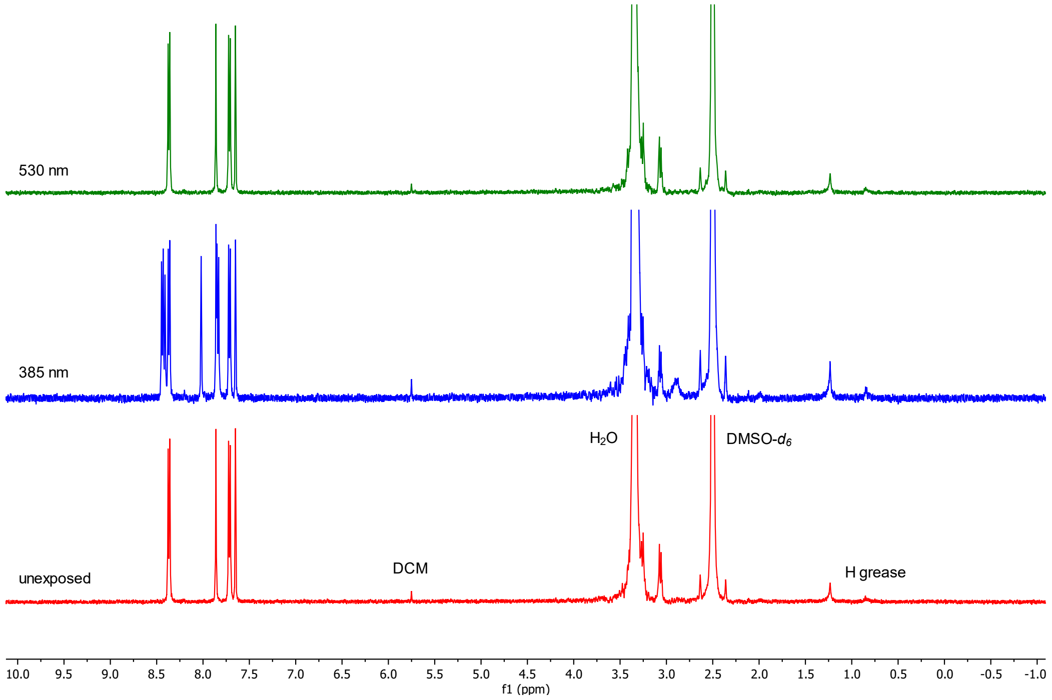

Figure S77: ^{1}H NMR spectra (500 MHz, DMSO-d_6, 298 K) of compound **13** (red), after irradiation for 30 s at 298 K with 385 nm (blue) and 530 nm (green).

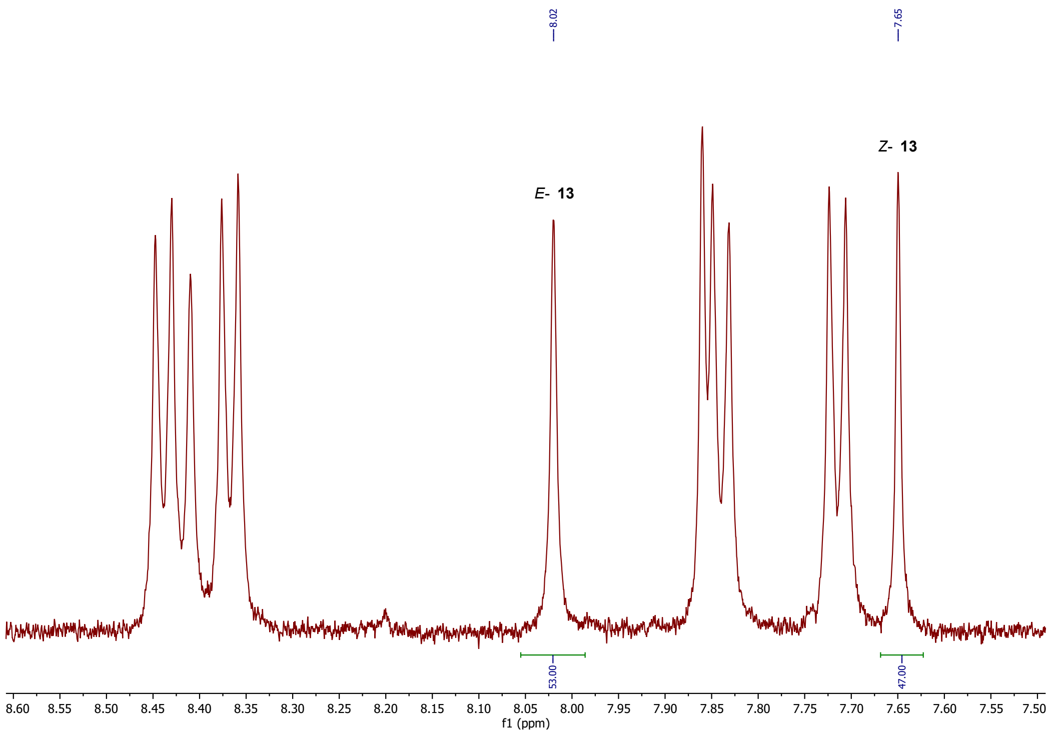

Figure S78: Excerpt of the ^{1}H NMR spectrum (500 MHz, DMSO-d_6, 298 K) of the photostationary state of compound **13** after irradiation with 385 nm for 30 s at 298 K and integration of the corresponding signals of the *Z* and *E* isomers (*Z*/*E*, 47:53).

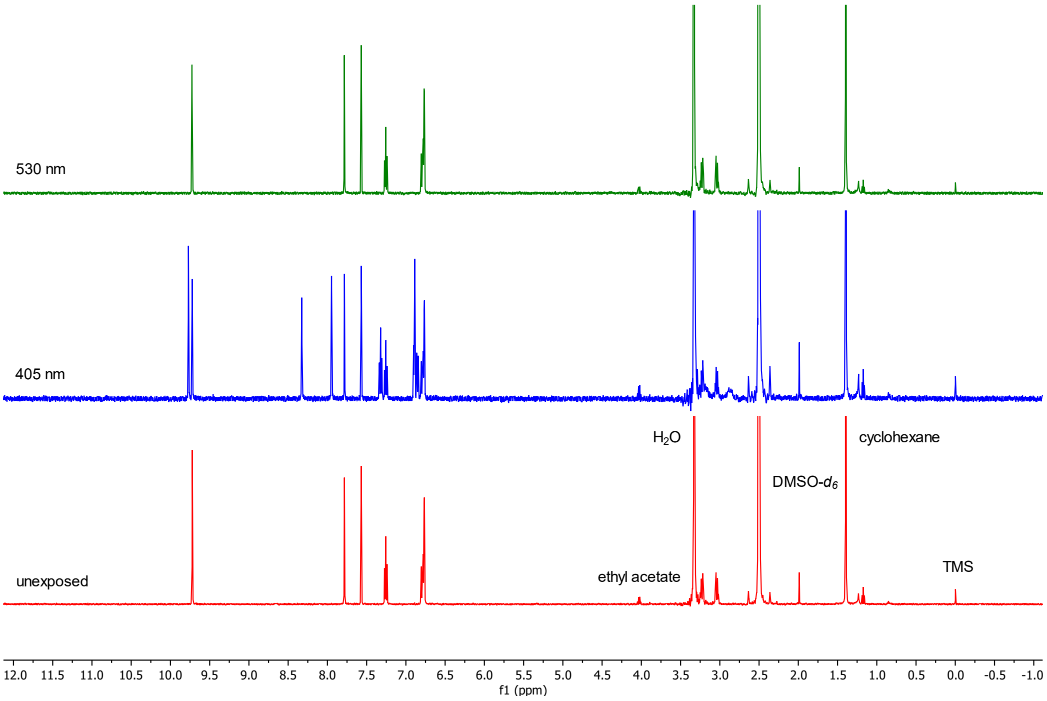

Figure S79: ^{1}H NMR spectra (500 MHz, DMSO-*d*$_6$, 298 K) of compound **14** (red), after irradiation for 30 s at 298 K with 405 nm (blue) and 530 nm (green).

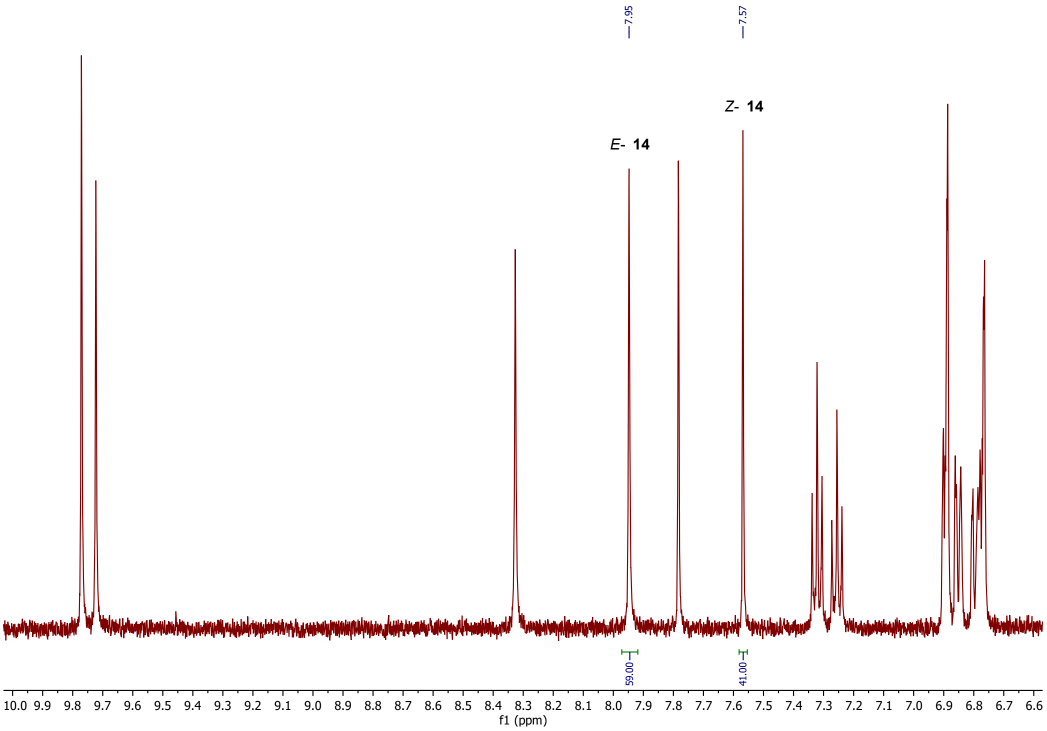

Figure S80: Excerpt of the ^{1}H NMR spectrum (500 MHz, DMSO-*d*$_6$, 298 K) of the photostationary state of compound **14** after irradiation with 405 nm for 30 s at 298 K and integration of the corresponding signals of the *Z* and *E* isomers (*Z*/*E*, 41:59).

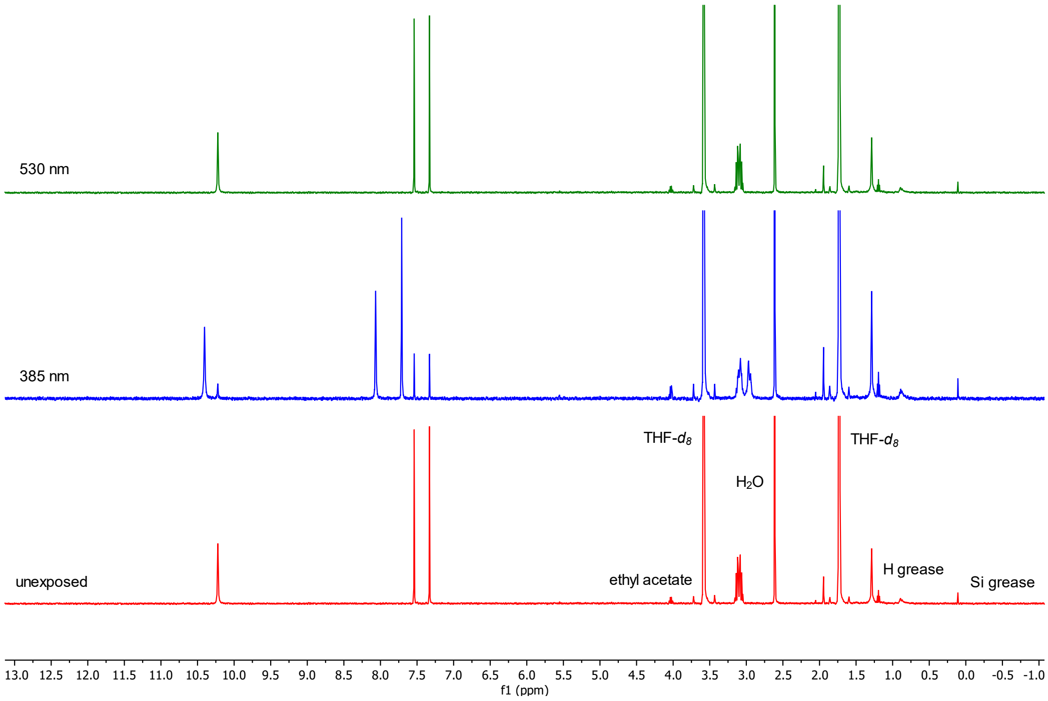

Figure S81: ^{1}H NMR spectra (500 MHz, THF-*d*8, 273 K) of compound **15** (red), after irradiation for 30 s at 273 K with 385 nm (blue) and 530 nm (green).

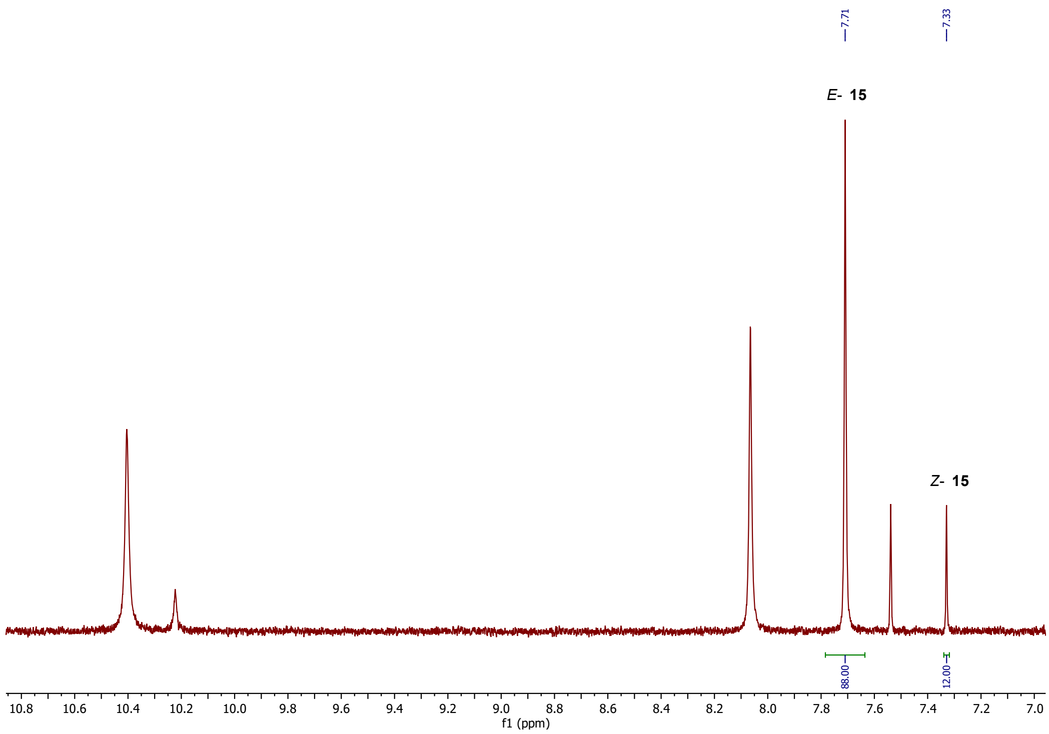

Figure S82: Excerpt of the ^{1}H NMR spectrum (500 MHz, THF-*d*8, 273 K) of the photostationary state of compound **15** after irradiation with 385 nm for 30 s at 273 K and integration of the corresponding signals of the *Z* and *E* isomers (*Z*/*E*, 12:88).

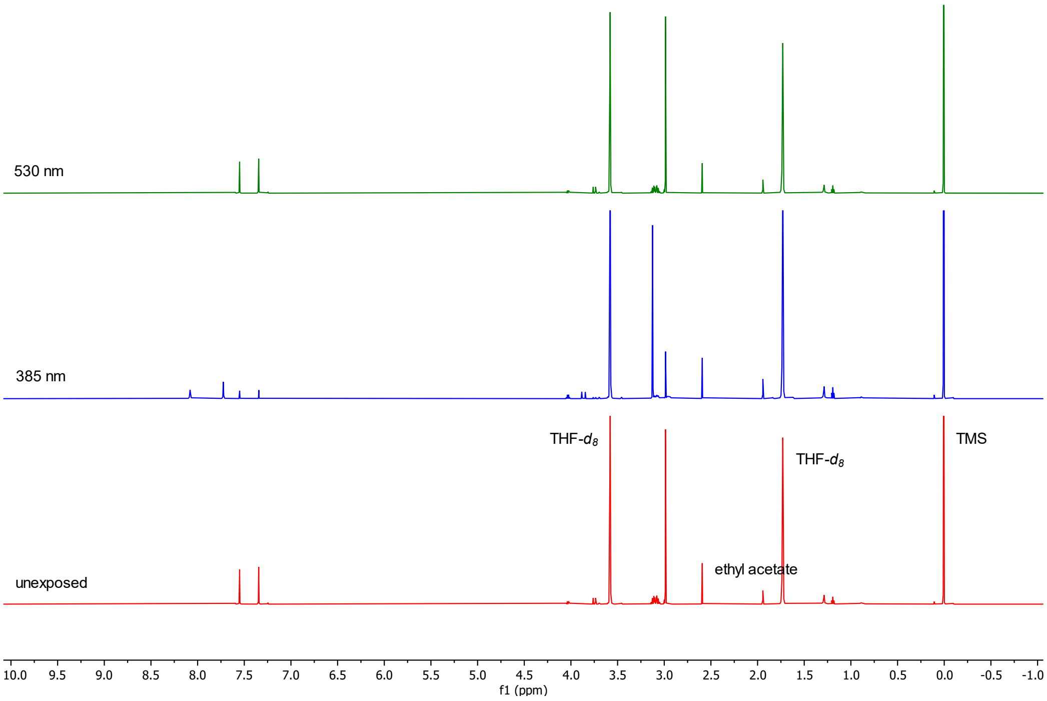

Figure S83: ^{1}H NMR spectra (600 MHz, THF-d_8, 273 K) of compound **16** (red), after irradiation for 30 s at 273 K with 385 nm (blue) and 530 nm (green).

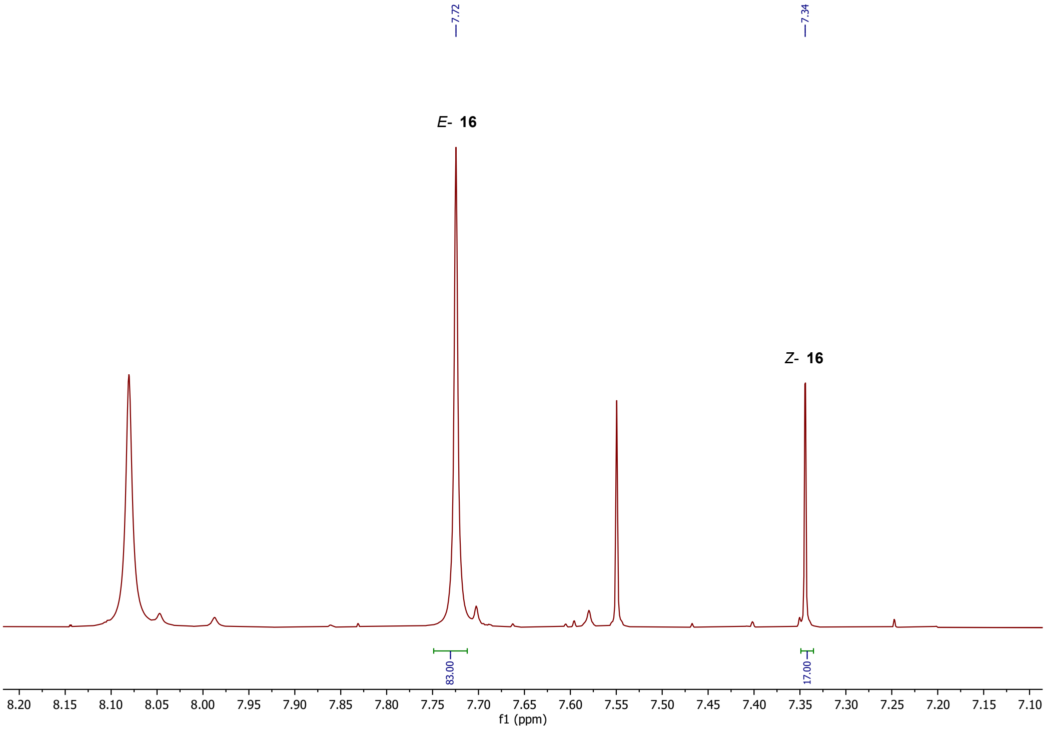

Figure S84: Excerpt of the ^{1}H NMR spectrum (600 MHz, THF-d_8, 273 K) of the photostationary state of compound **16** after irradiation with 385 nm for 30 s at 273 K and integration of the corresponding signals of the *Z* and *E* isomers (*Z*/*E*, 17:83).

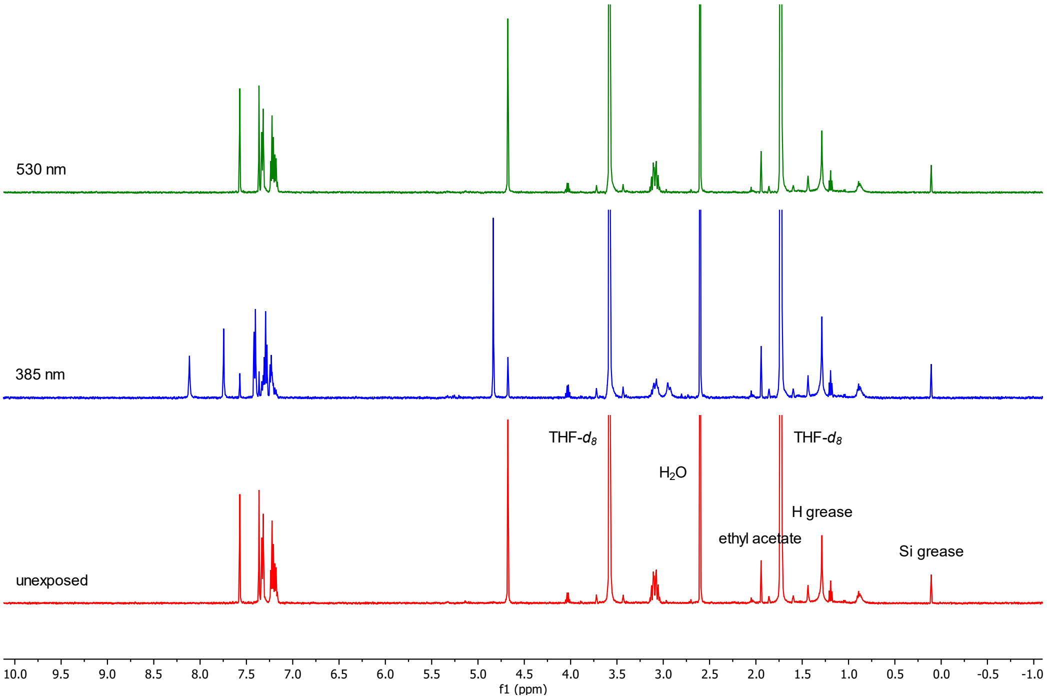

Figure S85: ^{1}H NMR spectra (500 MHz, THF-d_8, 273 K) of compound **17** (red), after irradiation for 30 s at 273 K with 385 nm (blue) and 530 nm (green).

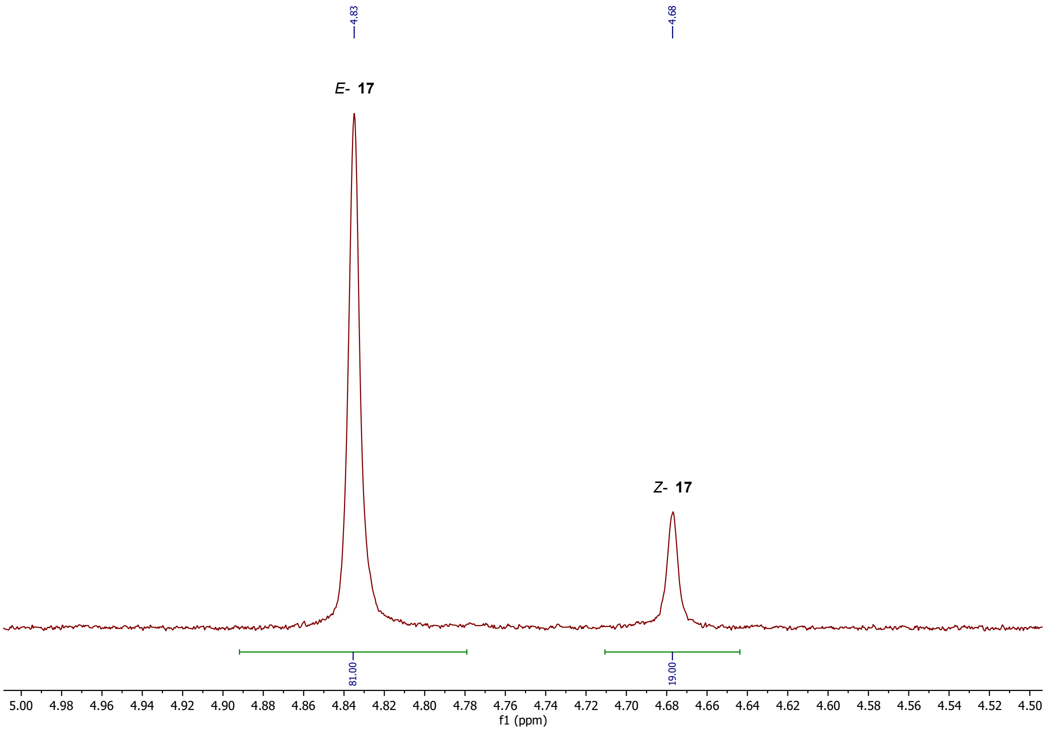

Figure S86: Excerpt of the ^{1}H NMR spectrum (500 MHz, THF-d_8, 273 K) of the photostationary state of compound **17** after irradiation with 385 nm for 30 s at 273 K and integration of the corresponding signals of the *Z* and *E* isomers (*Z*/*E*, 19:81).

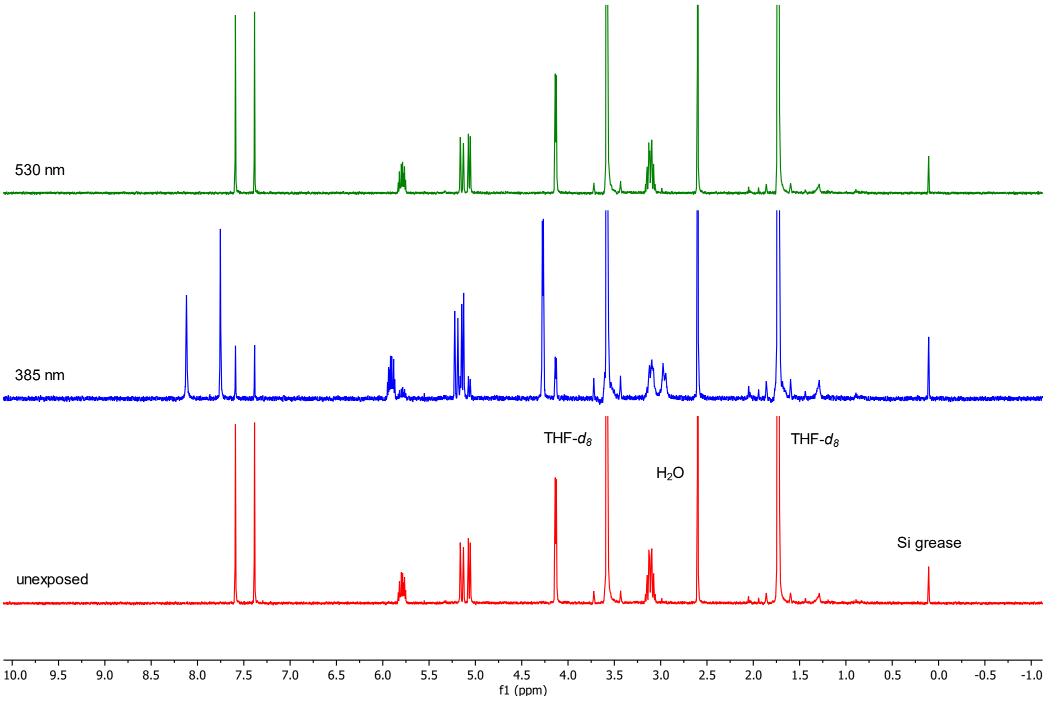

Figure S87: ^{1}H NMR spectra (500 MHz, THF-d_8, 273 K) of compound **18** (red), after irradiation for 30 s at 273 K with 385 nm (blue) and 530 nm (green).

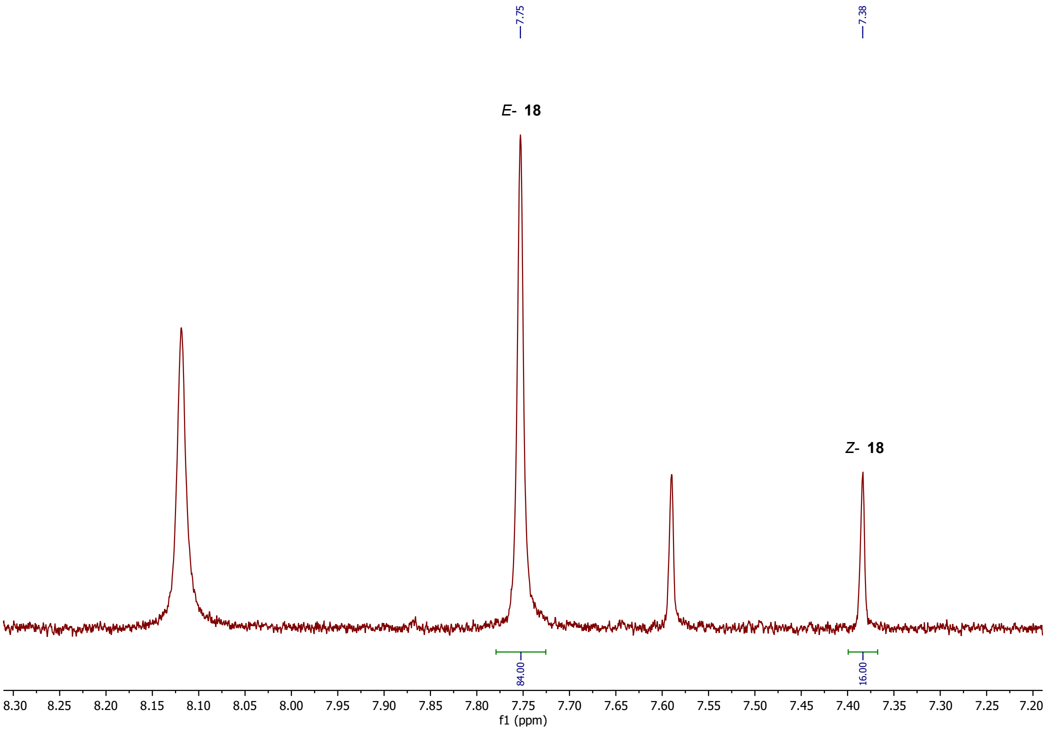

Figure S88: Excerpt of the ^{1}H NMR spectrum (500 MHz, THF-d_8, 273 K) of the photostationary state of compound **18** after irradiation with 385 nm for 30 s at 273 K and integration of the corresponding signals of the *Z* and *E* isomers (*Z*/*E*, 16:84).

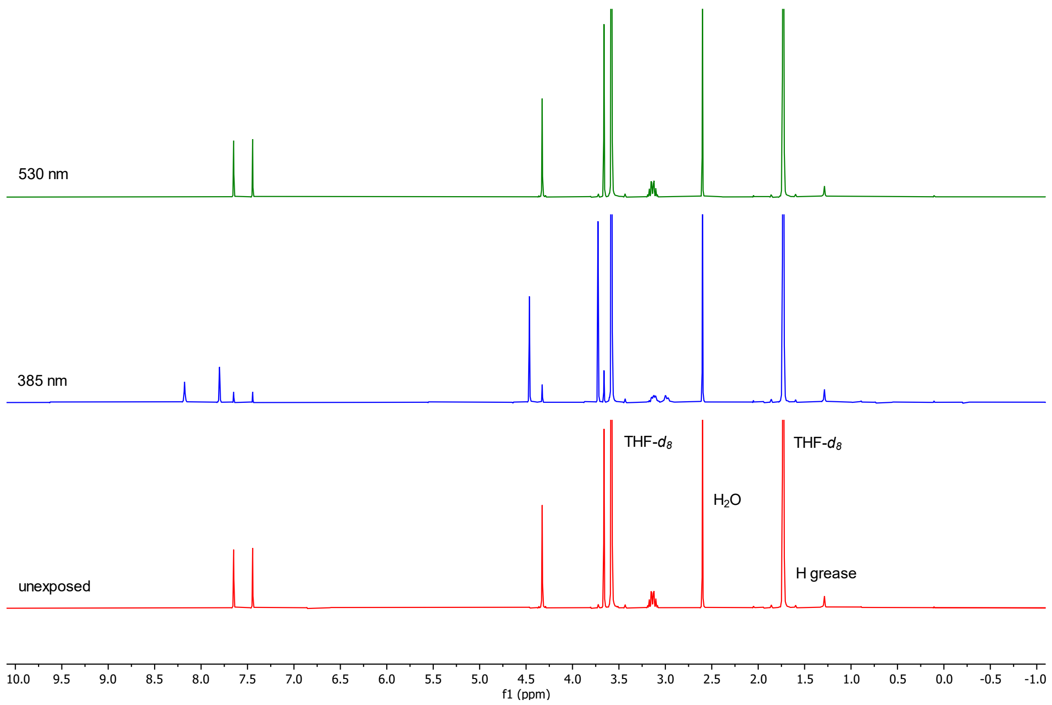

Figure S89: ^{1}H NMR spectra (500 MHz, THF-d_8, 273 K) of compound **19** (red), after irradiation for 30 s at 273 K with 385 nm (blue) and 530 nm (green).

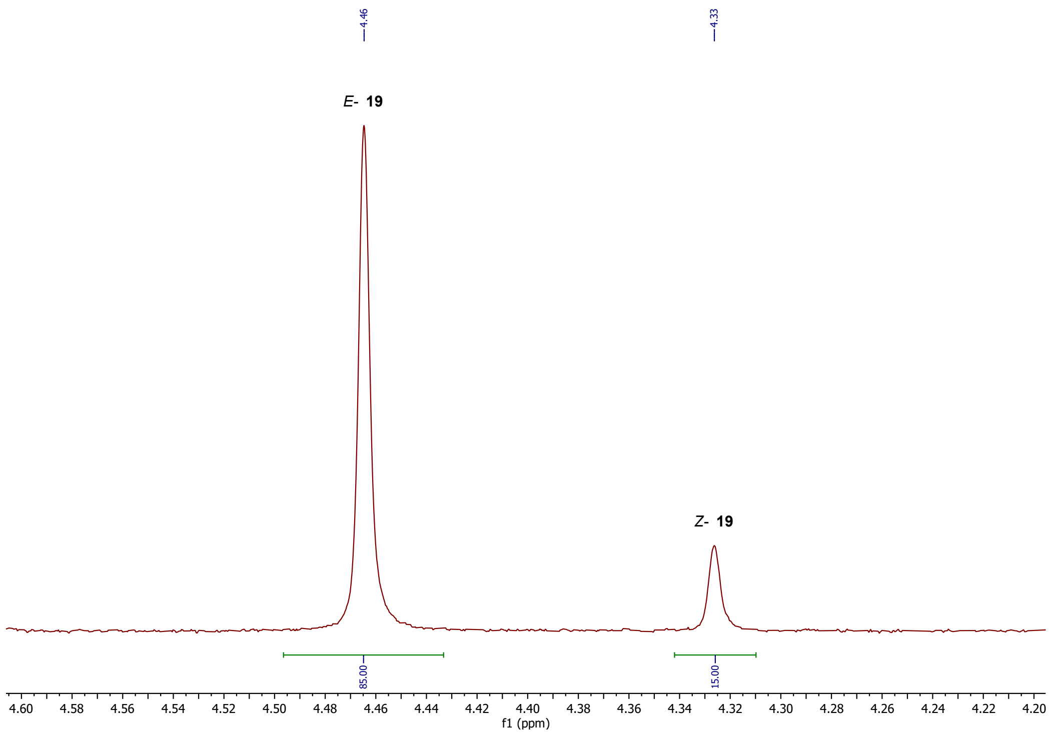

Figure S90: Excerpt of the ^{1}H NMR spectrum (500 MHz, THF-d_8, 273 K) of the photostationary state of compound **19** after irradiation with 385 nm for 30 s at 273 K and integration of the corresponding signals of the *Z* and *E* isomers (*Z*/*E*, 15:85).

S 5 Molecular modeling

S 5.1 Theoretical calculations of imide diazocines 9 and 15

The calculations were performed using the ORCA[3] program package. Geometries were optimized at the PBE/def2-SVP level of theory with Grimme's D4 dispersion correction.[4–8]

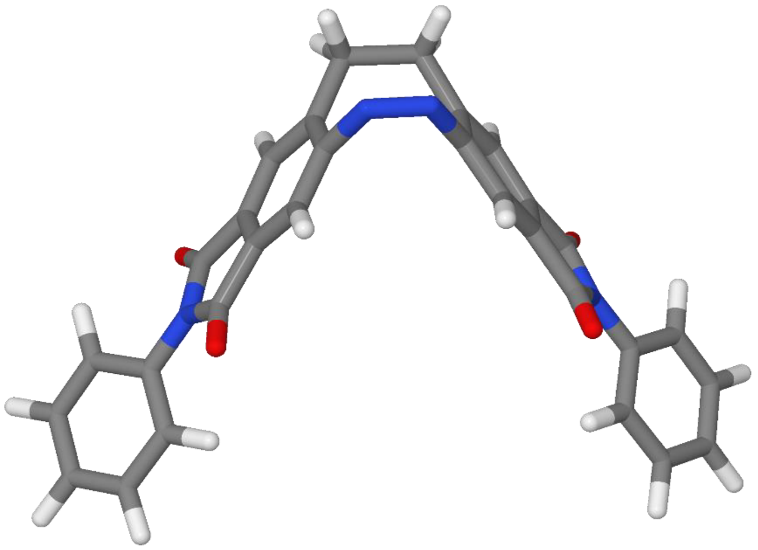

Figure S91: Theoretical structure of the *Z* isomer of compound **9**.

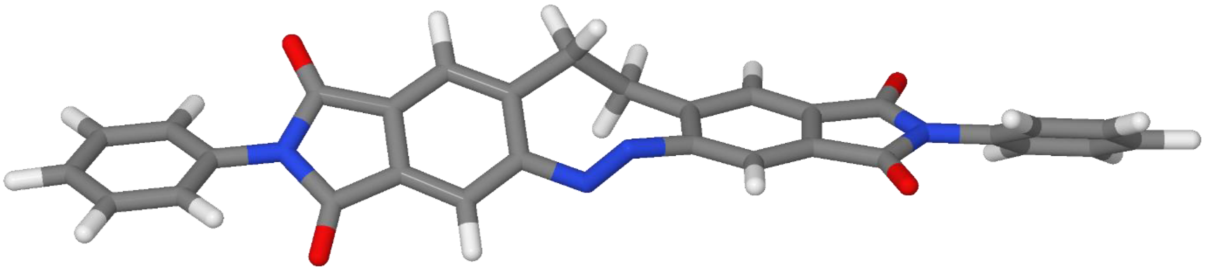

Figure S92: Theoretical structure of the *E* isomer of compound **9**.

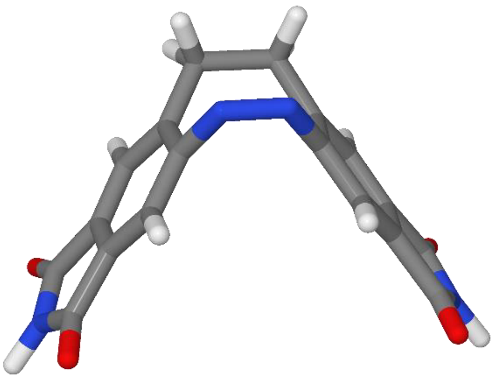

Figure S93: Theoretical structure of the *Z* isomer of compound **15**.

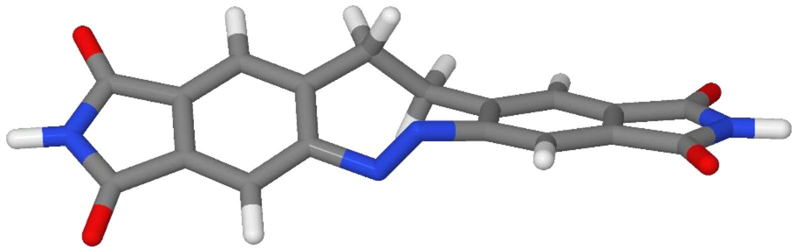

Figure S94: Theoretical structure of the *E* isomer of compound **15**.

S 5.2 Calculations of the motion amplitudes of differently substituted diazocines

During *Z* ⇄ *E* isomerization, the movement of the azo group (-N=N-) is transferred to the molecular scaffold of the diazocine. The rather small motion of the azo group is increased by driving a *Z*-boat → *E*-twist transition. In applications, this movement must in turn be transferred to the environment by suitable substituents. The resulting amplitude of movement depends on the position of the substituents and the distance from the azo group (lever). Figure S95 gives various distances of hydrogen substituents at the parent diazocine, parent ID **15** and phenyl substituted ID **9**.

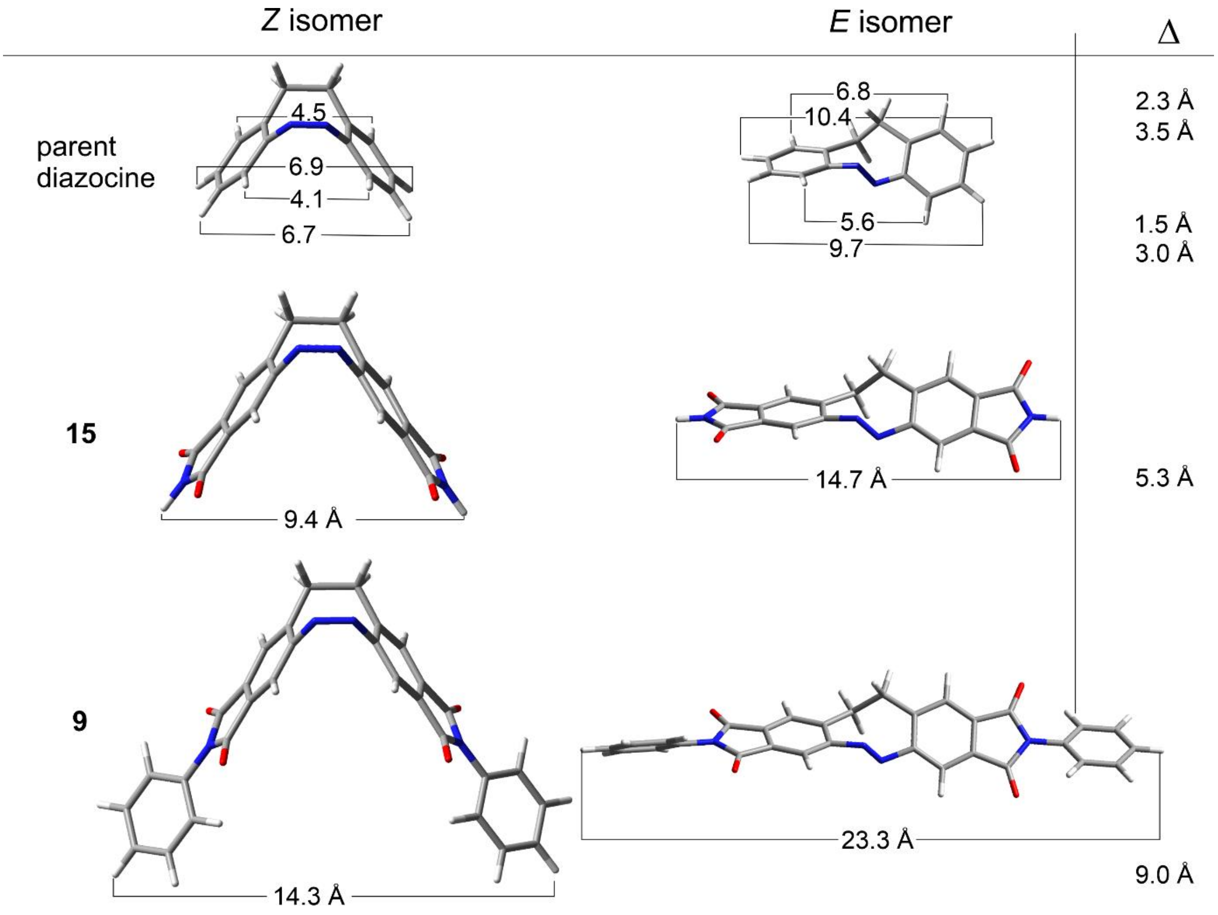

Figure S95: Change of distances (Δ) between hydrogen atoms at different positions upon isomerization in parent diazocine and derivatives **9** and **15**.

S 6 X-ray single crystal structure analysis

Data collections were performed with a XtaLAB Synergy, Dualflex, HyPix diffractometer that is equipped with a micro focus tube. The structures were solved with SHELXT [1] and refined with SHELXL [2] using Least Squares minimization. In all compounds the non-hydrogen atoms were refined anisotropic. The C-H H atoms were positioned with idealized geometry (methyl H atoms allowed to rotate but not to tip) and were refined isotropic with $U_{iso}(H)$ = 1.2 $U_{eq}(C)$ (1.5 for methyl H atoms) using a riding model. The asymmetric unit of compound **9** contains two crystallographically independent dichloromethane solvate molecules that are disordered and that were refined using a split model. There positions are not fully occupied. The asymmetric unit of compound **10** contains additionally one chloroform and one methanol molecule. The position of the chloroform molecule is occupied to only 80 %. The absolute structure was determined and is in agreement with the selected setting (Flack x = 0.038(19) by classical fit to all intensities and 0.021(5) from 3010 selected quotients (Parsons' method)). In the crystal structure of compound **16** the H atoms of both methyl groups are disordered and were refined using a split model with two orientations rotated by 60° relative to each other (AFIX 127). This structure was measured at 220 K, because at lower temperatures the crystal splits into different domains indicating for a destruction of the crystals. The asymmetric unit of compound **17** contains additionally one chloroform solvate molecule that is disordered and occupied to only 80% and that was refined using a split model with restraints. In the molecule one phenyl ring as well as the two N and opposite C atoms are also disordered and were refined using a split model. In compound **18** two crystallographically independent molecules are present. In one of them one of the two allyl groups is disordered and was refined using a split model with restraints (SADI). Selected crystal data and details of the structure determinations can be found in Table S2 and S3 and ORTEP plots are shown in Figures S96 – S102. CCDC-2419450 (**5**), CCDC-2419452 (**8**), CCDC-2419454 (**9**), CCDC-2419456 (**10**), CCDC-2419451 (**16**), CCDC-2419453 (**17**) and CCDC-2419455 (**18**) contain the supplementary crystallographic data for this paper. These data can be obtained free of charge from the Cambridge Crystallographic Data Centre via http://www.ccdc.cam.ac.uk/data_request/cif.

1. Sheldrick, G.M. (2015). Acta Cryst. A71, 3-8.
2. Sheldrick, G.M. (2015). Acta Cryst. C71, 3-8.

Table S2: Selected crystal data and details of the structure determinations for compounds **5**, **8**, **9** and **10**.

	**5**	**8**	**9**	**10**
Formula	$C_{22}H_{20}N_2O_8$	$C_{33}H_{23}Cl_3N_4O_4$	$C_{31.75}H_{20.8}Cl_{3.5}N_4O_4$	$C_{33.8}H_{26.8}Cl_{2.4}N_4O_7$
MW /g mol^{-1}	440.40	549.82	646.40	686.07
Crystal system	triclinic	triclinic	monoclinic	orthorhombic
Space group	*P-1*	P-1	I2/a	$Pna2_1$
a /Å	8.8959(2)	7.94327(10)	29.0406(16)	17.12725(9)
b /Å	10.8197(2)	10.71284(14)	8.0115(3)	27.31323(15)
c /Å	11.9993(2)	14.84117(18)	28.2411(14)	6.79802(4)
α /deg	64.470(2)	93.2678(10)	90	90
β /deg	80.629(2)	95.1647(10)	117.500(7)	90
γ /deg	77.352(2)	100.0313(11)	90	90
V /$Å^3$	1013.81(4)	1235.15(3)	5828.2(6)	3180.12(3)
T /K	100.0(2)	100.0(2)	100.0(2)	100.0(2)
Z	2	2	8	4
D_{calcd} /g cm^{-3}	1.443	1.478	1.473	1.433
μ /mm^{-1}	0.941	3.707	3.653	2.623
2 θ_{max} /deg	159.622	160.07	160.934	160.632
Refl. collected	20734	14827	20080	79776
Refl. unique	4292	5200	6175	6865
R_{int}	0.0171	0.0149	0.0506	0.0352
Refl. [$F_0 > 4\ \sigma(F_0)$]	4226	5030	5646	6815
Parameters	294	327	433	437
R_1 [$F_0 > 4\ \sigma(F_0)$]	0.0324	0.0338	0.0876	0.0468
wR_2 (all data)	0.0836	0.0854	0.2340	0.1293
GOF	1.055	1.056	1.113	1.060
$\Delta\rho_{max;\ min}$ /e $Å^{-3}$	0.33/-0.21	0.51/-0.76	1.27/-0.51	1.12/-0.45

Table S3: Selected crystal data and details of the structure determinations for compounds **16**, **17** and **18**.

	**16**	**17**	**18**
Formula	$C_{20}H_{14}N_4O_4$	$C_{33}H_{23}Cl_3N_4O_4$	$C_{24}H_{18}N_4O_4$
MW /g mol^{-1}	374.35	645.90	426.42
Crystal system	monoclinic	monoclinic	triclinic
Space group	$P2_1/c$	$P2_1/c$	P-1
a /Å	16.3660(5)	11.3990(4)	10.44890(14)
b /Å	8.0137(2)	21.3927(6)	13.56011(16)
c /Å	13.7112(4)	13.0033(5)	16.50284(17)
α /deg	90	90	109.0565(11)
β /deg	106.779(3)	112.014(4)	106.1166(12)
γ /deg	90	90	97.7776(11)
V /Å^3	1721.68(8)	2939.73(19)	2056.46(5)
T /K	220.0(2)	100.0(2)	100.0(2)
Z	4	4	4
D_{calcd} /g cm^{-3}	1.434	1.459	1.377
μ /mm^{-1}	0.736	3.213	0.793
2 θ_{max} /deg	160.194	160.91	160.77
Refl. collected	11137	20646	32245
Refl. unique	3653	6183	8645
R_{int}	0.0253	0.0316	0.0187
Refl. [$F_0 > 4\ \sigma(F_0)$]	3218	4992	7971
Parameters	256	508	595
R_1 [$F_0 > 4\ \sigma(F_0)$]	0.0439	0.0570	0.0380
wR_2 (all data)	0.1293	0.1644	0.1018
GOF	1.057	1.042	1.038
$\Delta\rho_{max;\ min}$ /e Å^{-3}	0.18/-0.18	0.29/-0.48	0.40/-0.26

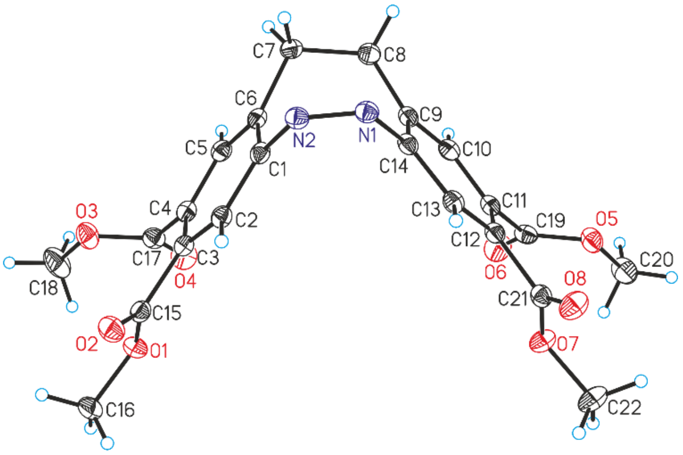

Figure S96. Crystal structure of compound **5** with labeling and displacement ellipsoids drawn at the 50% probability level.

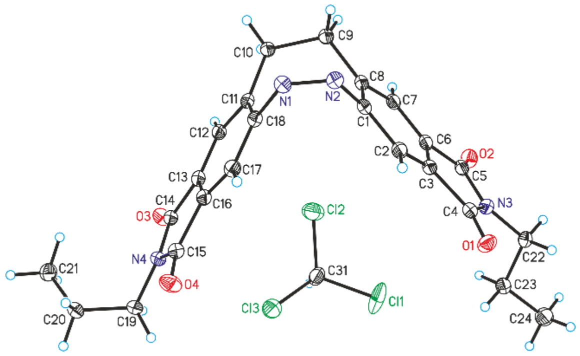

Figure S97. Crystal structure of compound **8** with labeling and displacement ellipsoids drawn at the 50% probability level.

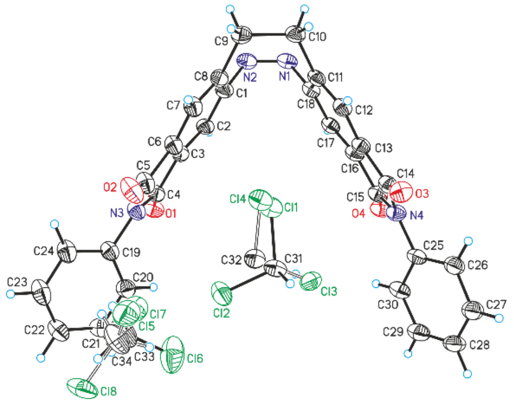

Figure S98. Crystal structure of compound **9** with labeling and displacement ellipsoids drawn at the 50% probability level.

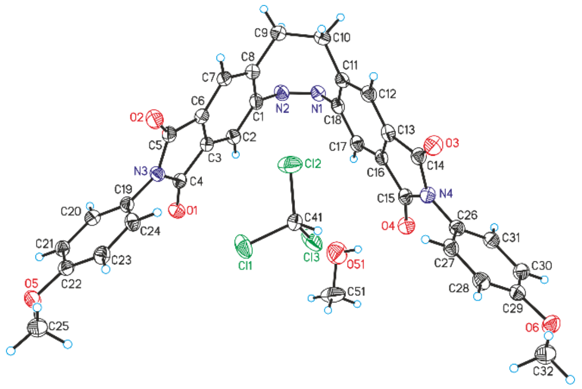

Figure S99. Crystal structure of compound **10** with labeling and displacement ellipsoids drawn at the 50% probability level.

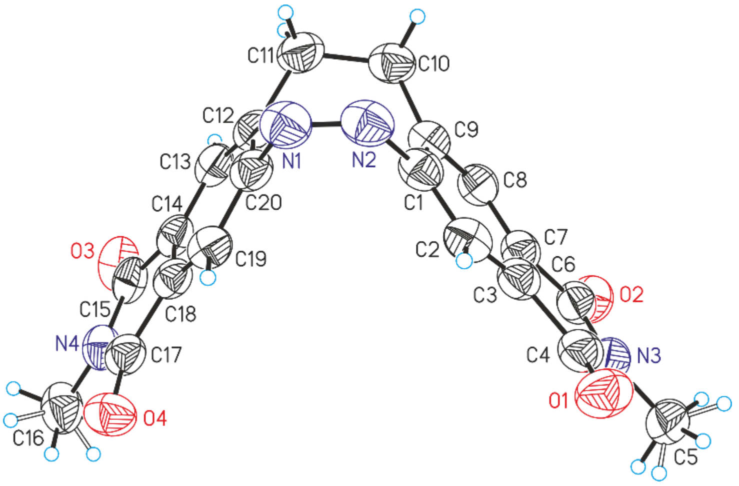

Figure S100. Crystal structure of compound **16** with labeling and displacement ellipsoids drawn at the 50% probability level. The disorder of the methyl H atoms is shown with full and open bonds.

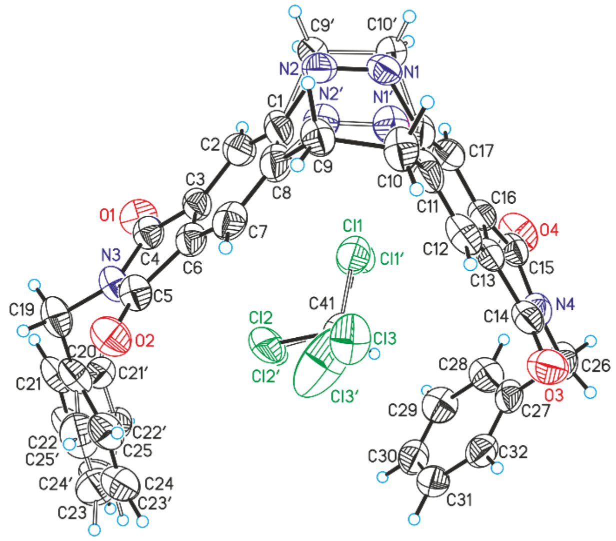

Figure S101. Crystal structure of compound **17** with labeling and displacement ellipsoids drawn at the 50% probability level. The disorder is shown with full and open bonds.

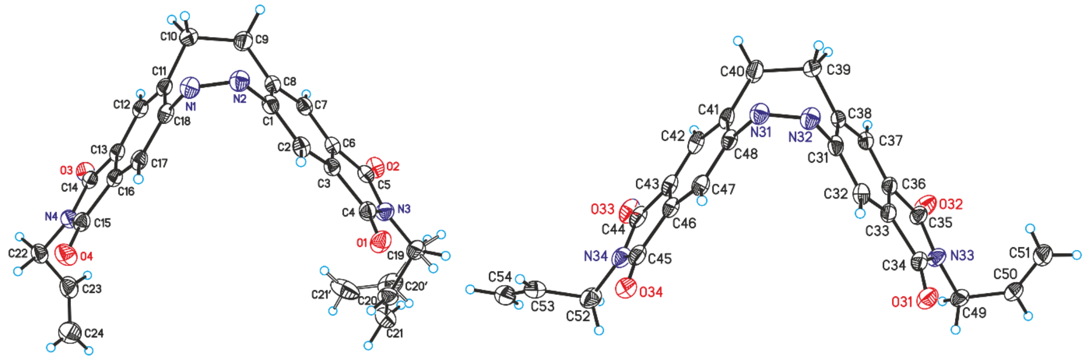

Figure S102. Crystal structure of the two crystallographically independent molecules in compound **18** with labeling and displacement ellipsoids drawn at the 50% probability level. The disordering of the allyl group is shown with full and open bonds.

Crystal growth

Single crystals of compound **5** were obtained by gas phase diffusion using cyclohexane and ethyl acetate. Single crystals of compounds **8** – **10** and **16** – **18** were obtained by gas phase diffusion using chloroform and methanol.

Structure descriptions

Compounds **8**, **9**, **10** and **17** form clathrates, which is not the case for compounds **5**, **16** and **18**. All structural motifs seem to be driven by the goal, to fill the opening of the diazocine molecules and this can be achieved by inclusion of solvents or by self-interpenetrating of two neighbored molecules. The only exception is compound **16**, which could be due to the fact that it is the smallest derivative and can pack tightly even without solvent and/or that this molecule has the smallest opening, which is too small at least for the chloroform used for its crystallization. The packing of the molecules in this compound is, typical for many organic compounds, a bit confusing and characterized by the densest packing (Figure S103). There is also no sign of π-π-interactions.

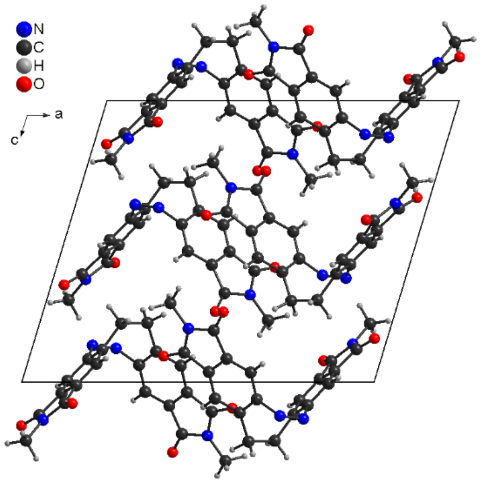

Figure S103. Crystal structure of compound **16** with view along the crystallographic *b*-axis direction.

Compounds **5** and **18** also crystallize without solvent but in these structures a closed-packing is achieved by self-interpenetration of two neighbored molecules forming dimer-like units (Figure S104). In compound **5** these units are located on centers of inversion, whereas in compound **18** they are formed by each two crystallographically independent molecules.

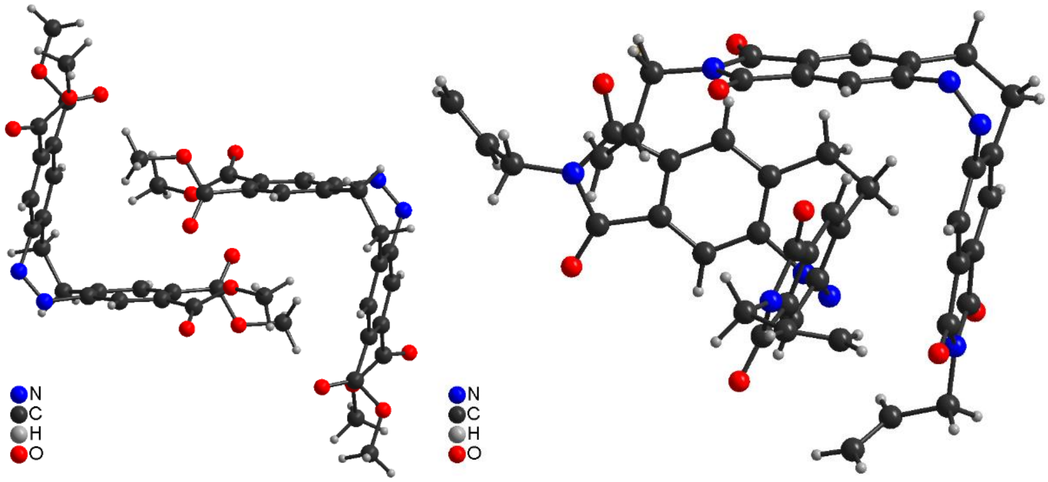

Figure S104. Crystal structure of compound **5** (left) and compound **18** (right) showing the dimer-like units formed by self-interpenetration.

The packing of the molecules in compounds **5** and **18** is very similar, because in both compounds the dimeric units are arranged in columns that proceed along the *b*-axis direction (Figure S105). In compound **5** these columns consists of centrosymmetric units, whereas in compound **18** each two dimeric units are related by centers of inversion.

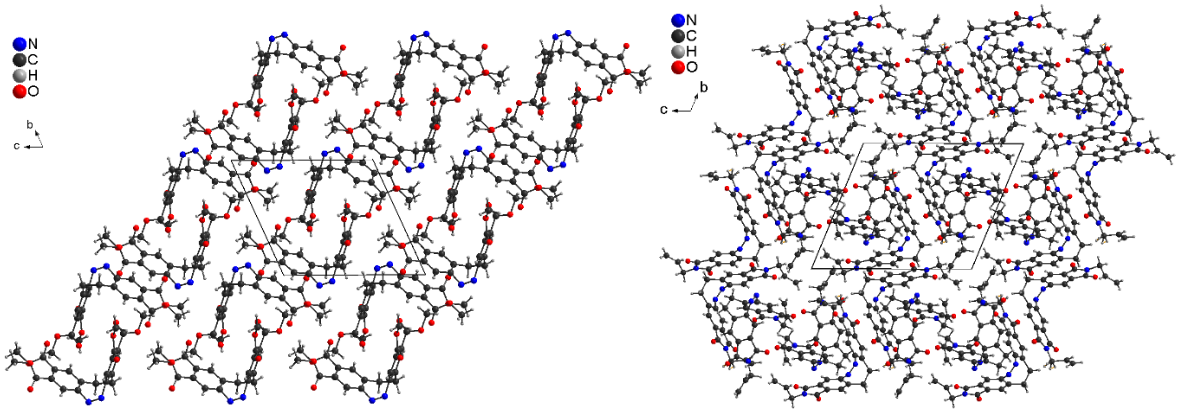

Figure S105. Crystal structure of compound **5** (left) and of compound **18** (right) with view along the *a*-axis direction.

In all remaining compounds the opening of the diazocine derivatives is filled by solvate molecules. In compound **17** one chloroform molecule that is disordered in two

orientations is exactly located in the opening of the host molecule (Figure S101). In the crystal structure the molecules are arranged into layers that are stacked along the crystallographic *c*-axis direction. By this arrangement channels are formed, in which the chloroform solvate molecules are located (Figure S106). The ring planes between neighbored molecules are parallel, indicating for *π*-*π*-interactions.

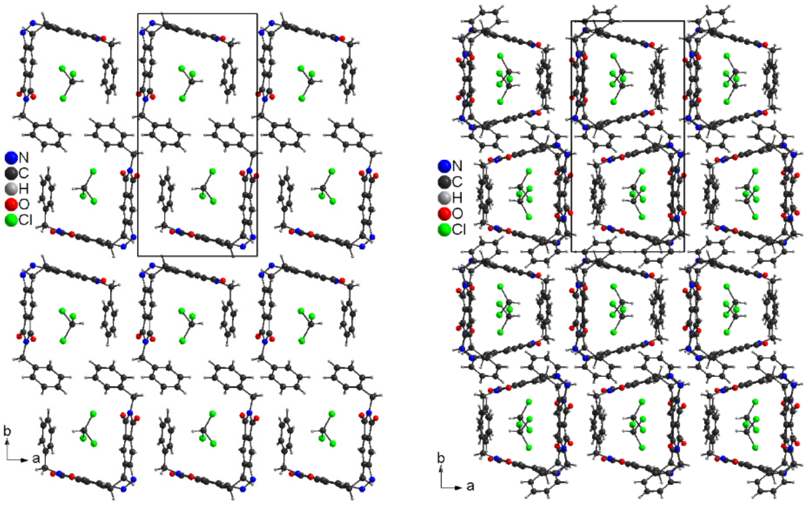

Figure S106. Crystal structure of compound **17** with view along the crystallographic *c*-axis.

In compound **9** disordered dichloromethane molecules are embedded. Only one of the two crystallographically independent solvate molecules are located exactly in the opening of the molecules. The second solvate molecule is slightly shifted because a channel structure is formed. The channels proceed along the *b*-axis and neighboring channels are connected by *π*-*π*-interactions (Figure S107).

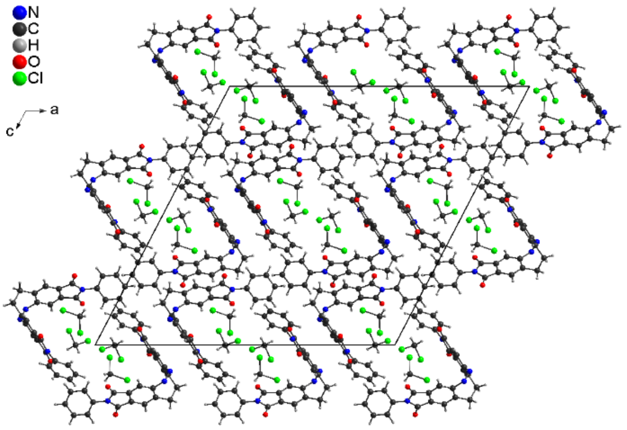

Figure S107. Crystal structure of compound **9** with view along the *b*-axis direction.

In compound **8** one chloroform solvate molecule is located in the opening of the diazocine molecule. As in compound **17** the molecules are arranged in layers. The layers are stacked in a way that channels are formed, in which the solvate molecules are located (Figure S108). As in compounds **10** and **17** the ring planes of neighboring molecules are parallel, indicating for by *π*-*π*-interactions.

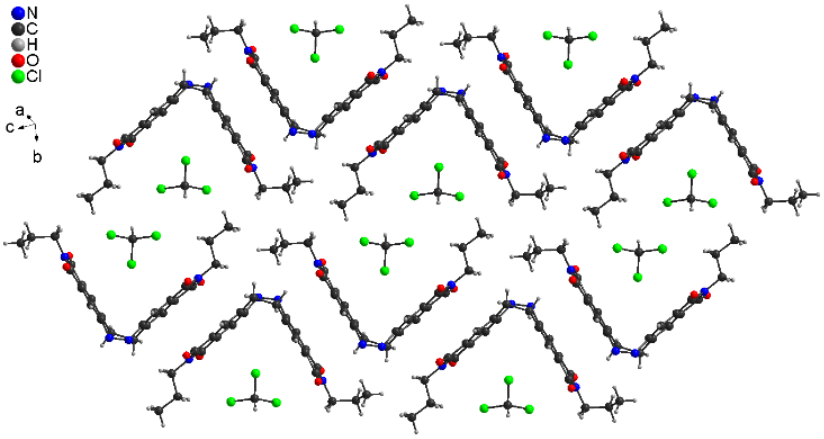

Figure S108. Crystal structure of compound **8** with view along the channels.

Finally, in compound **10**, both chloroform and methanol are embedded in the opening of the diazocine molecules. The host molecules are packed in a way that channels along the *c*-axis are formed, in which the solvate molecules are located (Figure S109). The methanol guest molecule is connected to the diazocine host molecule *via* intermolecular O-H···O hydrogen bonding.

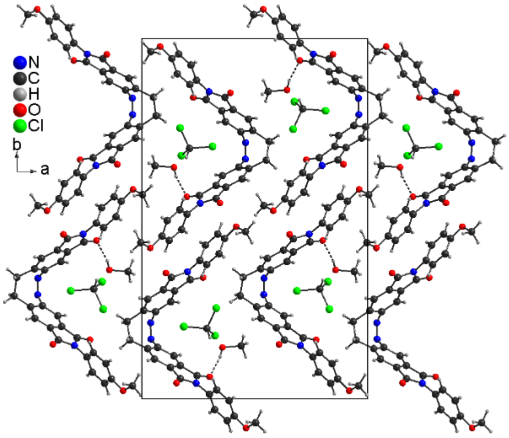

Figure S109. Crystal structure of compound **10** with view along the crystallographic *c*-axis. Intermolecular O-H···O hydrogen bonding is shown as dashed lines.

S 7 Precipitation of the *E* isomer of imide diazocine 9

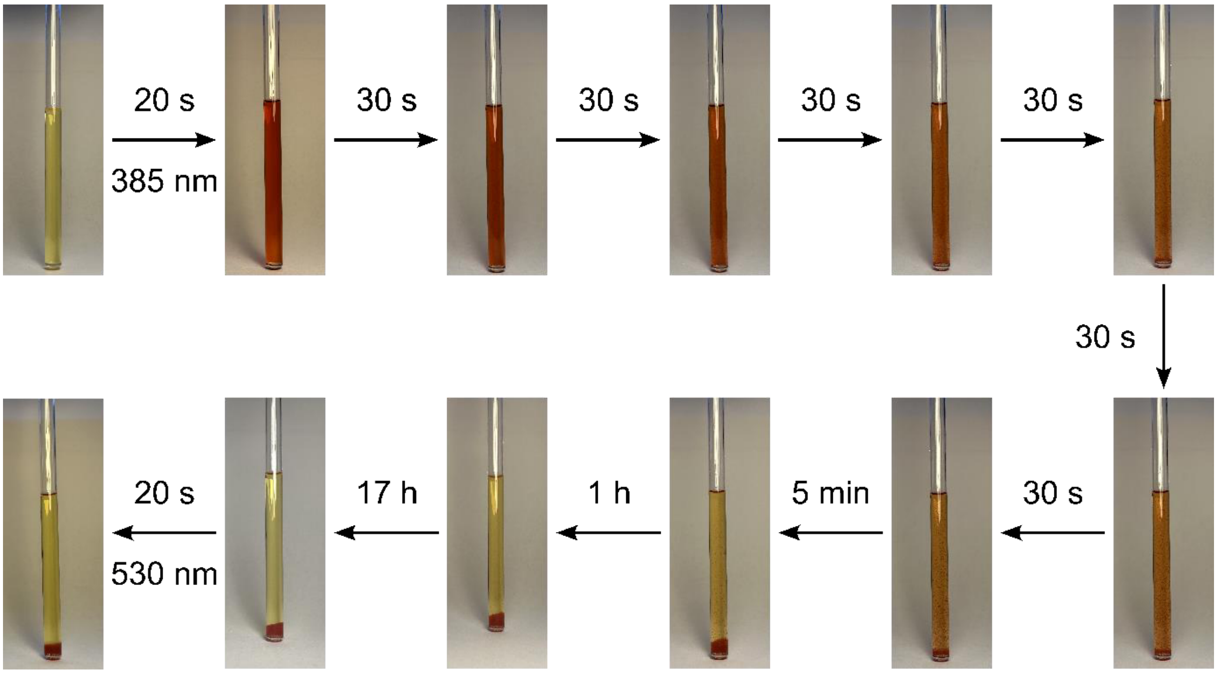

Figure S110: Time course of the precipitation of the red *E* isomer of ID **9** (2.00 mg in 550 µL THF) after irradiation with 385 nm for 20 s at 298 K. Irradiation with 530 nm for 20 s at 298 K does not redissolve the red solid.

S 8 References

[1] G. R. Fulmer, A. J. M. Miller, N. H. Sherden, H. E. Gottlieb, A. Nudelman, B. M. Stoltz, J. E. Bercaw, K. I. Goldberg, *Organometallics* **2010**, *29*, 2176–2179.

[2] M. Hao, T. Liu, Y. Xiao, L.-K. Ma, G. Zhang, C. Zhong, Z. Chen, Z. Luo, X. Lu, H. Yan, L. Wang, C. Yang, *Chem. Mater.* **2019**, *31*, 1752–1760.

[3] F. Neese, F. Wennmohs, U. Becker, C. Riplinger, *J. Chem. Phys.* **2020**, *152*, 224108.

[4] E. Caldeweyher, S. Ehlert, A. Hansen, H. Neugebauer, S. Spicher, C. Bannwarth, S. Grimme, *J. Chem. Phys.* **2019**, *150*, 154122.

[5] E. Caldeweyher, C. Bannwarth, S. Grimme, *J. Chem. Phys.* **2017**, *147*, 34112.

[6] F. Weigend, *Phys. Chem. Chem. Phys.* **2006**, *8*, 1057–1065.

[7] F. Weigend, R. Ahlrichs, *Phys. Chem. Chem. Phys.* **2005**, *7*, 3297–3305.

[8] J. P. Perdew, K. Burke, M. Ernzerhof, *Phys. Rev. Lett.* **1997**, *78*, 1396.
